# Stable and ordered amide frameworks synthesised under reversible conditions which facilitate error checking

**DOI:** 10.1038/s41467-017-01423-5

**Published:** 2017-10-24

**Authors:** David Stewart, Dmytro Antypov, Matthew S. Dyer, Michael J. Pitcher, Alexandros P. Katsoulidis, Philip A. Chater, Frédéric Blanc, Matthew J. Rosseinsky

**Affiliations:** 1 Department of Chemistry, University of Liverpool, Liverpool, L69 7ZD UK; 2 Diamond Light Source, Harwell Campus, Didcot, Oxfordshire OX11 0DE UK; 3 Stephenson Institute for Renewable Energy, University of Liverpool, Liverpool, L69 7ZD UK

## Abstract

Covalent organic frameworks (COFs) are network polymers with long-range positional order whose properties can be tuned using the isoreticular chemistry approach. Making COFs from strong bonds is challenging because irreversible rapid formation of the network produces amorphous materials with locked-in disorder. Reversibility in bond formation is essential to generate ordered networks, as it allows the error-checking that permits the network to crystallise, and so candidate network-forming chemistries such as amide that are irreversible under conventional low temperature bond-forming conditions have been underexplored. Here we show that we can prepare two- and three-dimensional covalent amide frameworks (CAFs) by devitrification of amorphous polyamide network polymers using high-temperature and high-pressure reaction conditions. In this way we have accessed reversible amide bond formation that allows crystalline order to develop. This strategy permits the direct synthesis of practically irreversible ordered amide networks that are stable thermally and under both strong acidic and basic hydrolytic conditions.

## Abstract (web summary)

Irreversible bond formation in covalent organic frameworks leads to amorphous materials. Here, the authors show that crystalline and ultra-stable covalent amide frameworks can be accessed by devitrification of amorphous polyamide networks.

## Introduction

Assembling crystalline frameworks based on strong covalent bonds between molecular units is challenging because bond formation is local whereas crystalline order requires positional coherence over many nanometres. Although the synthesis of amorphous framework materials such as porous polymers^1^ is more straightforward, crystalline materials are desirable because they have well-defined homogeneous environments which are easier to characterise and understand, thus enhancing the ability to design materials for specific applications.

For a covalent network to be crystalline^2^ it is essential that all the molecular units adopt a conformation which, when repeated in the bulk, results in long-range positional order. The variability in bond lengths, angles and dihedral angles both within the molecular units and between them can introduce non-ideal conformations and/or connectivity^3, 4^ whose additive effect can result in the formation of amorphous materials^5^ rather than the desired crystalline ones.

Spatially-correlated covalent bond formation is challenging to achieve in non-reversible reaction conditions because once a unit is added in a non-ideal position, it is locked in place. Crystalline frameworks such as zeolites, metal organic frameworks (MOFs) and covalent organic frameworks (COFs) are typically synthesised under conditions that permit error-checking^6^ through repeated making and breaking of the network-forming bonds enhancing the role of thermodynamic control of crystal growth. If a non-ideal conformation is incorporated into the network, it can be removed and then replaced by another unit which may have the correct conformation/connectivity, thus increasing the likelihood that molecular units will achieve their crystalline positions.

Amide bonds are highly stable and are considered practically irreversible under typical ambient conditions (rate constant for hydrolysis of amides^7^ are of the order of 10^−11^ s^−1^), including in acidic or basic aqueous solutions^8^, and are thermally robust^9^. Furthermore, amides are formed from cheap highly abundant components which display extensive structural and chemical diversity. As such amides present a promising candidate for use as network-forming bonds in COFs if reaction conditions that allow crystalline networks to be formed reversibly from bonds which are strong enough to be non-labile under ambient conditions can be found. Typically amides are prepared in non-equilibrium processes *e.g*. by activation of carboxylic acids through conversion to acid chlorides followed by reaction with amines, or continuous removal of the condensate throughout the reaction in order to drive it to completion. Both of these methods purposefully minimise the reverse reaction with the aim of maximising yield, however this reversibility is essential for the formation of crystalline frameworks. The aim of this work therefore, is to identify reaction conditions which will enable the synthesis of stable covalent amide frameworks. The post-synthetic oxidation of two two-dimensional crystalline imine frameworks, formed from reversible bonds, to form amide frameworks has been reported recently^10^; however, this approach is markedly different to our direct route.

Here we show two amorphous polyamide networks that were synthesised using a typical acid chloride route. Polyamide(1,3,5-tricarboxybenzene-net-*trans*-1,4-cyclohexanediamine) (PATnC) consists of a combination of tri- and di-functional constitutional units derived from trimesic acid (TMA) and *trans-*1,4-cyclohexyldiamine (CHDA). Polyamide(4,4′,4″,4”’-tetra(carboxyphenyl)methane-net-*trans-*1,4-cyclohexanediamine) (PATCnC) consists of tetra- and di-functional constitutional units derived from 4,4′,4″,4‴-methanetetrayltetrabenzoic acid (MTAB) and CHDA. The combinations of tri- plus di-functional and tetra- plus di-functional units were chosen as these combinations could potentially form regular 2D hexagonal and 3D four-coordinate nets, respectively^11, 12^. Both of these materials were formed under non-reversible conditions, precluding error-checking thus resulting in amorphous materials as expected^13^. To overcome the reversibility problem, we have borrowed conceptually from the synthesis of crystalline silicas^14^. Amorphous silica is highly stable at ambient conditions and the problem faced is much the same, i.e., each added unit must locate its ideal crystalline conformation. The synthesis of crystalline silica forces these strong Si–O bonds to form reversibly by the application of harsh conditions^15^, namely high temperatures, high pressures and a strongly hydrolytic environment. A related approach has been used by Unterlass and co-workers^16, 17^ in the synthesis of ordered linear polyimides, although in these non-framework systems the strong covalent bonds do not produce crystallinity. By using conditions which are far from ambient and particularly harsh in terms of COF synthesis, we have been able to harness the reversibility of amide bond formation and thus have synthesised two- and three-dimensional amide-based COFs directly.

## Results

### Synthesis of CAF-1 by devitrification

**Fig. 1:**
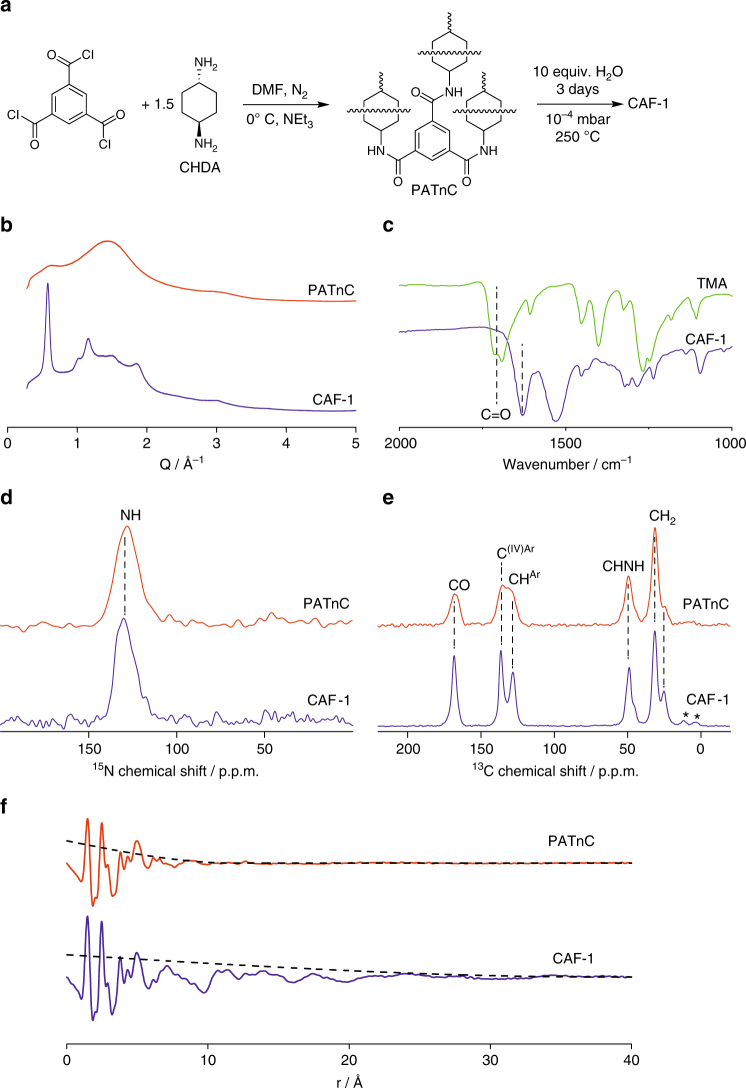
Covalent Amide Framework 1 (CAF-1). **a** Synthetic pathway for CAF-1 via the synthesis of amorphous polyamide(1,3,5-tricarboxybenzene-net-*trans*-1,4-cyclohexanediamine) (PATnC) followed by an optimised devitrification procedure. **b** Synchrotron powder X-ray diffraction (PXRD) patterns of PATnC and CAF-1. **c** IR spectra of trimesic acid (TMA) and CAF-1 indicating the continued presence of the amide bond. The C = O stretch is indicated in the figure. **d**, **e** ^15^N and ^13^C CP MAS NMR spectra of PATnC and CAF-1 obtained at 9.4 T with the corresponding spectral assignments (Supplementary Table 1), asterisks in **e** denote spinning side-bands. **f**, Pair distribution function (PDF) data of PATnC and CAF-1. A spherical correction function, *f*_sphere_(*r*), used to estimate the coherence length is shown as a dashed line

PATnC was dried by evacuation at 100 °C to remove all water and was loaded into a Pyrex tube with 10 molar equivalents of water which was then sealed at 10^−4^ mbar. The tube was then placed in a Parr pressure reactor containing water, and the reactor sealed and heated at 250 °C in an oven for 3 days (Fig. 1a, Supplementary Methods). The use of the water-containing Parr reactor is essential to balance the pressure generated inside the Pyrex tube at reaction temperature (Supplementary Fig. 1).

After cooling the solid was washed repeatedly with water and methanol resulting in a quantitative yield. ^1^H NMR spectra of the filtrate showed no organic components and indicates that the network does not degrade under synthesis or washing conditions. The PXRD pattern of the resultant solid (Fig. 1b) showed that the network had devitrified, developing long-range order demonstrated by the observation of Bragg peaks, from this hydrothermal treatment and we term this material covalent amide framework 1 (CAF-1). The IR spectrum of CAF-1 (Fig. 1c,) showed a shift in the carbonyl stretch from 1689 cm^−1^ in TMA to 1628 cm^−1^ in CAF-1 indicating the presence of an amide^18^ (Supplementary Fig. 3 for full spectrum and Supplementary Fig. 4 for comparison of C=O stretch region). Furthermore, CHN analysis of CAF-1 found an empirical formula of C_18_H_21_N_3_O_3_.*x*H_2_O where *x* = 1.20 to 1.65 (Supplementary Table 4) which conforms to the expected 1:1.5 molar ratio of TMA and CHDA components required to form a hexagonal net. If a sample of dry PATnC is subjected to this process without the presence of water then there is no observable change in the amorphous polymer (Supplementary Fig. 5). This confirms that it is the action of the water which is generating the crystalline order within the material. It is likely that the water is repeatedly breaking amide bonds which then reform through a solid state polymerization^19^ (SSP) mechanism and in the process allows the network to equilibrate into the ordered structure observed.

In order to confirm that CAF-1 is a polyamide network, ^15^N cross polarisation (CP) magic angle spinning (MAS) NMR spectra of PATnC and CAF-1 were acquired (Fig. 1d) and reveal that a single ^15^N peak at 128–130 p.p.m. is present, unequivocally demonstrating that the network is formed from amide bonds^18^. In addition, the ^13^C CP MAS NMR spectra of PATnC and CAF-1 (Fig. 1e) showed all the expected resonances (Supplementary Table 1), the apparent multiplicity of the ***C***HNH carbon around 49 p.p.m. arises from residual ^13^C–^14^N dipolar coupling not averaged out by MAS^20^ while the two *C*H_2_ signals observed around 30 p.p.m. results from structural disorder (see below and Supplementary Fig. 22). It is also worth pointing out that the NMR resonances observed for both PATnC and CAF-1 NMR spectra appear at the same chemical shift confirming the same chemical structure, but that the lines of CAF-1 are narrower than in PATnC (for example, the CONH ^13^C line width is 380 Hz in CAF-1 and 730 Hz in PATnC, Supplementary Table 1) consistent with devitrification and the improvement in crystallinity observed in the PXRD data (Fig. 1b).

Pair distribution function (PDF) data were collected for PATnC and CAF-1 (Fig. 1e, Supplementary Figs. 16 and 17). In order to access an approximate coherence length for the two phases a spherical correction function, *f*_sphere_(*r*)^21^, was fit to the data to model a correlated sphere of atoms surrounded by completely disordered density and a spherical diameter extracted; values of 12 Å (1 repeat unit—in a single hexagonal sheet the translation distance of one TMA to another is 13 Å) and 38 Å (3 repeat units) were determined for PATnC and CAF-1, respectively. Although *f*_sphere_(*r*) assumes that the order will be isotropic, which is unlikely to be the case, it still demonstrates the increase in ordering length-scale in CAF-1 upon devitrification of PATnC. An estimation of the volume-weighted column height determined from the integral breadth of the CAF-1 Bragg peaks gave a crystallite size of 75.55(8) Å.

Two additional routes to CAF-1 were also developed beginning with a mixture of molecular starting materials or a salt of those same starting materials being sealed in tubes under the same conditions as those used to synthesise CAF-1 from PATnC (Supplementary Note 1). In both cases this resulted in the formation of CAF-1. It is likely that SSP occurs in situ to form an amorphous polymer and then devitrification takes place in the same manner as for the synthesis from PATnC. The reaction parameters in the PATnC-based route were optimised including the reaction time and temperature, and the amount of water added, the different routes to CAF-1 were also compared (Supplementary Note 2 and Supplementary Fig. 9–12). It was found that synthesis from the molecular starting materials results in the most crystalline material, however it also results in the lowest yield. Unless otherwise stated the data presented herein is from the PATnC-based route to CAF-1 as this allows direct control of the reaction parameters in the devitrification process.

### Synthesis of CAF-2 by devitrification

**Fig. 2:**
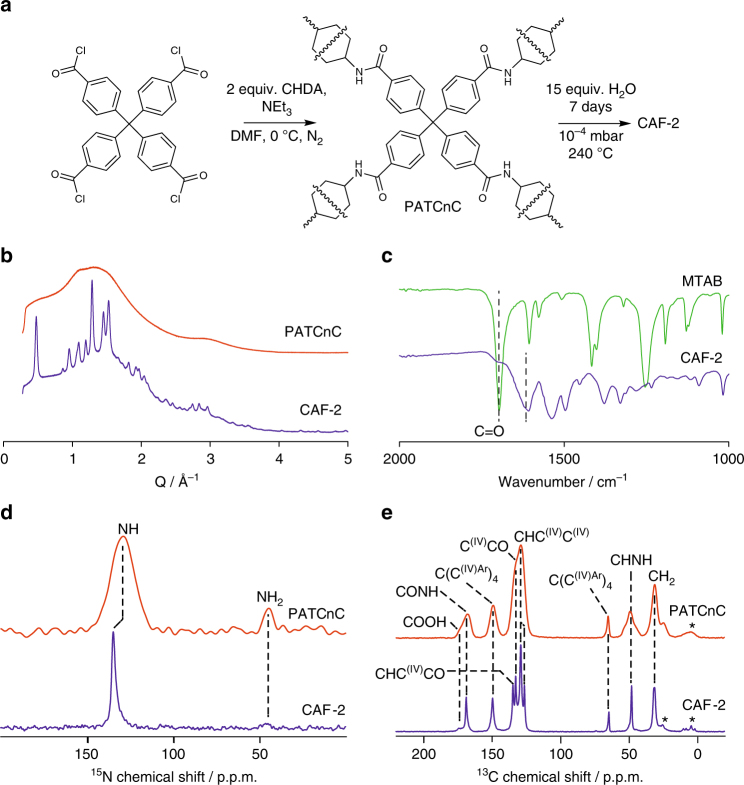
Covalent Amide Framework 2 (CAF-2). **a** Synthetic pathway for CAF-2 via the synthesis of polyamide(4,4′,4″,4”’-tetra(carboxyphenyl)methane-net-*trans-*1,4-cyclohexanediamine) (PATCnC) from 4,4′,4″,4”’-methanetetraaryltetrabenzoyl chloride and *trans*-1,4-cyclohexyldiamine (CHDA), followed by its devitrification. **b** Synchrotron PXRD patterns of PATCnC and CAF-2. **c** IR traces of 4,4′,4″,4‴-methanetetrayltetrabenzoic acid (MTAB) and CAF-2 which indicates the continued presence of the amide bond. The presence of the C = O stretches are indicated in the figure. **d**, **e** ^15^N and ^13^C CP MAS NMR spectra of PATCnC and CAF-2 obtained at 9.4T with the corresponding spectral assignments (Supplementary Table 1), asterisks in **e** denote spinning side-bands

PATCnC was dried under vacuum at 120 °C to remove all water and was then subjected to the devitrification method as described for CAF-1 (at 240 °C for 7 days with 15 molar equivalents of water, cooling at 0.1 °C min^−1^) to yield Covalent Amide Framework 2 (CAF-2). PXRD analysis of the three-dimensional CAF-2 clearly shows the development of long-range order after devitrification with enhanced crystallinity and a volume-weighted mean column height of 13.6(1) nm compared to that of two-dimensional CAF-1 of 7.55(8) nm (Fig. 2b). The IR data show a shift of the carbonyl stretch from 1699 cm^−1^ in MTAB to 1610 cm^−1^ in CAF-2 indicating the presence of the amide bonds (Fig. 2c, Supplementary Fig. 13–15). CHN analysis (Supplementary Methods) of CAF-2 shows a reasonable agreement to the empirical formula C_41_H_40_N_4_O_4_.*x*H_2_O where *x* = 11.55 corresponding to the 1:2 molar ratio of MTAB to CHDA units required for a four-connected net.

The presence of the amide bonds in CAF-2 was also conclusively proven by the observation of a strong ^15^N CP MAS NMR signal at 129–135 p.p.m. (Supplementary Table 1) typical of amides^18^ (Fig. 2d). A weaker ^15^N peak is also detected in the ^15^N CP MAS spectrum of both PATCnC and CAF-2, and is assigned to an amine nitrogen, suggesting that there are unreacted amine groups still present in the devitrified material; it is difficult to determine whether these are free diamine molecules trapped in the pores or if this resonance is associated with end groups of the polymer. ^13^C CP MAS NMR spectra of PATCnC and CAF-2 showed all their anticipated carbons (Fig. 2e, and Supplementary Table 1), although an additional broad resonance is observed at 174 p.p.m. and assigned to a carboxylic acid carbon, the difference in shift (5 p.p.m.) between this resonance and the carbonyl amide carbon (169 p.p.m.) being typical^18^. This indicates that there are either unreacted MTAB molecules trapped in the pores of CAF-2 or that there is a significant population of COOH end groups. Finally, the NMR lines of CAF-2 are much narrower than for PATCnC (CONH ^13^C line width of 120 vs. 660 Hz for CAF-2 and PATCnC, respectively, Supplementary Table 1), consistent with enhanced crystallinity upon devitrification shown by PXRD (Fig. 2b).

The MAS NMR and PDF data both indicate that the amorphous PATnC and PATCnC are compositionally homogeneous (both organic units are in the same local bonding environments for both materials), indicating that transport of the building blocks themselves is not needed to achieve crystallinity—their distribution in the amorphous material is already homogeneous. The lack of crystalline order arises from disorder in the relative orientations of trigonal or tetrahedral linker moieties. At high temperatures, this can be reduced by the breaking and reforming of the amide bonds in the presence of water, allowing the development of coherent orientations which produce the ordered structures of CAF-1 and CAF-2.

### Structural Analysis of CAF-1

**Fig. 3:**
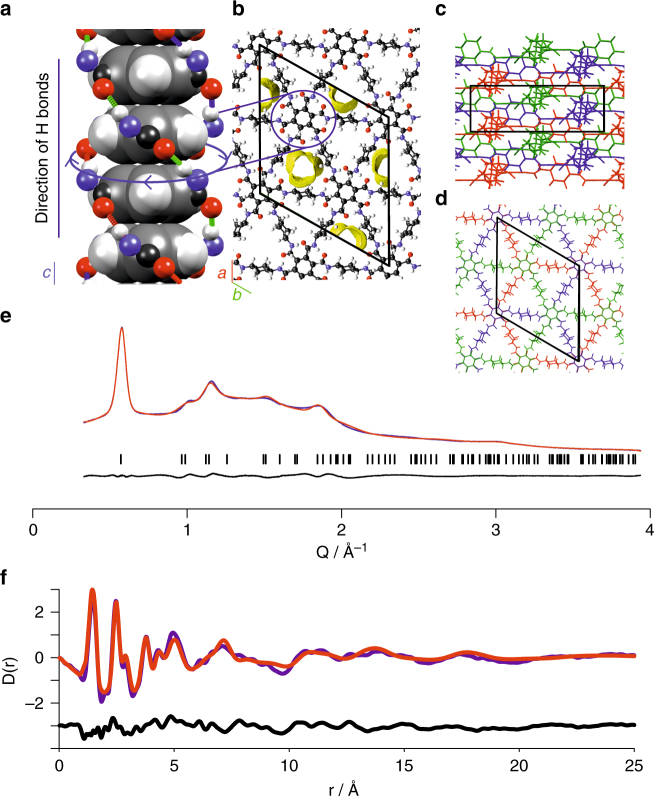
Structural model of CAF-1. **a** A single TMA stack showing one possibility for the arrangement of the H-bond triple helix, Van der Waals radii are used to depict atomic positions within the benzene ring, showing typical π-π stacking. The blue arrows indicate the handedness of the triple helix which is formed by three independent chains of H bonds, shown in red, blue and green, while the direction of the H bonds is considered to be from the N-H proton towards the C=O oxygen. **b** The Connolly surface for a 1.5 Å probe in the pores of the computed structure of CAF-1 are shown in yellow and the unit cell is overlaid. **c**, **d** schematic diagrams of the three independent CAF-1 sheets in a unit cell looking down the a- and c-axis respectively. **e** Rietveld refinement of an ABC stacked structure, observed synchrotron PXRD pattern in blue, calculated in red and difference in black. **f** PDF refinement of the same ABC stacked structure as used for the Rietveld refinement, observed in purple, calculated in red and difference in black

First we fitted the PDF data of PATnC to a single hexagonal sheet (Supplementary Fig. 16) constructed of CHDA and TMA fragments, confirming the hexagonal amide network connectivity. The correlations up to 5 Å in the PDF of PATnC and CAF-1 are identical, demonstrating retention of the local molecular connectivity upon devitrification (Supplementary Fig. 17), which is also supported by the assignment of the ^15^N and ^13^C CP MAS NMR spectra for both CAF-1 and PATnC. Next in silico models of candidate crystal structures for CAF-1 were constructed in which the hexagonal sheets were stacked with various motifs and then relaxed using Density Functional Theory (DFT). PXRD patterns were predicted from these relaxed models for the different stacking motifs and the ABC stacking model which best matched the experimental data was adopted (Supplementary Fig. 18). The orientations of the cyclohexyl and carbonyl units in the calculated model produce two types of triangular channel, one smaller (2.9 Å) and one larger (3.6 Å), with three of each type of channel present in the unit cell. A DFT model (Supplementary Note 3) in space group *R*3 was relaxed containing nine water molecules residing within the energetically favoured larger channels (Supplementary Tables 7–10), and the resulting structure was used to Rietveld fit the observed Bragg data without refining the atomic parameters. The refinement using the DFT model showed a moderately good fit (Fig. 3e) indicating that the unit cell and structure are consistent with the observed data. The refined unit cell dimensions were *a* = 22.021(12) Å and *c* = 6.927(2) Å. The proposed structural model was then refined using the PDF data; the result is shown in Fig. 3f. The data shows a good fit in the region up to 5 Å and a broadly consistent fit thereafter, indicating that the proposed DFT model is in agreement with the observed data.

The proposed ABC stacked structure consists of buckled hexagonal sheets comprising TMA and CHDA units in a 1:1.5 molar ratio covalently linked by amide bonds as confirmed by ^15^N CP MAS NMR, IR and CHN. Three such sheets are stacked in the unit cell (Fig. 3c and d) with each sheet translated by 1/3 of a unit cell compared to those adjacent to it, giving an ABC stacking with rhombohedral symmetry (*R*3, *a* = 22.021(12) Å and *c* = 6.927(2) Å). The buckling of the hexagonal sheets at the cyclohexyl linkers facilitates the formation of columns of TMA units separated by 3.3–3.4 Å, consistent with π-π stacking interactions. The three amide protons of one TMA unit H-bond to the carbonyl oxygens of the adjacent TMA unit in the column, resulting in a triple helix H bonding pattern propagating along the TMA column (Fig. 3a).

The computed structure in space group *R*3 contains only one H bonding pattern, and all cyclohexyl linkers have equivalent orientations. In principle, however, the N-H bonds can be oriented either up or down the column, and the helices can have either clockwise or anti-clockwise handedness, giving rise to four possible H bonding patterns for each TMA column (Fig. 3a, Supplementary Fig. 22). These different H bonding patterns may coexist within CAF-1, giving rise to structural disorder, in line with the small difference in computed energy between these configurations (Supplementary Fig. 22). DFT calculations show that different H bonding patterns also lead to different orientations of the cyclohexyl linkers producing a model with mixed H bonding patterns which shows a splitting of the CH_2_ resonance in predicted ^13^C NMR spectra (Supplementary Fig. 23), as observed experimentally in the ^13^C CP MAS NMR spectrum (Fig. 1e). This is consistent with the presence of multiple H bonding patterns and cyclohexyl group orientations contributing to structural disorder within CAF-1 which is not included in the refined structural model presented and is consistent with the relatively short PDF-derived coherence length and PXRD-derived domain size. Due to the potential for many different vibrational modes in such a complex structure as CAF-1 we have used PDF as a diagnostic tool for crystallinity and for testing structural models only.

### Structural analysis of CAF-2

**Fig. 4:**
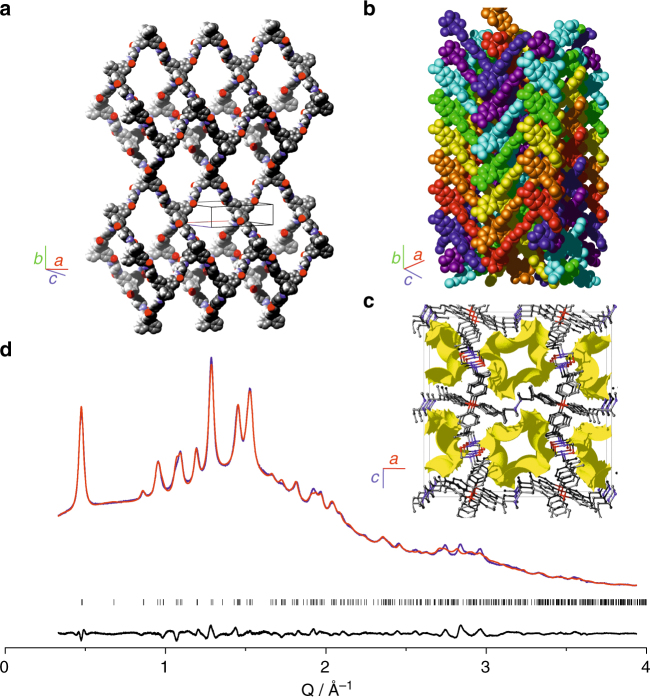
Structural analysis of CAF-2. **a** Structure of a single CAF-2 net, hydrogens omitted for clarity. **b** Representation of the 7-fold interpenetration of the diamondoid nets shown in **a** in CAF-2, with each net coloured differently. **c** View along the *b* axis of CAF-2, showing all nets; a Connolly surface with a 1.7 Å probe is shown in yellow to represent the resulting one-dimensional porosity. Hydrogens are omitted for clarity. **d** Rietveld refinement of CAF-2, observed synchrotron PXRD pattern in blue, calculated in red and difference in black

The structural building blocks of CAF-2 are tetrahedral MTAB-derived units and near-linear CHDA-derived linking units, which are connected directly by amide bonds (as confirmed by ^13^C and ^15^N CP MAS NMR, IR and CHN analysis, see Fig. 4a): this combination is expected to produce a three-dimensional diamondoid net^22^. A structural model for CAF-2 was developed by comparing simulated PXRD patterns of 3D diamondoid nets with different degrees of interpenetration, and then relaxing the most promising candidates by DFT to produce starting models for Rietveld refinement (Supplementary Note 4); simulated annealing of water positions was then followed by a rigid-body Rietveld refinement to produce the final crystallographic model shown in Fig. 4 (Supplementary Note 5), which was confirmed by successful simulation of the ^13^C NMR parameters which match the experimental CP MAS NMR data (Supplementary Fig. 28). The refined structure consists of 7 interpenetrated diamondoid nets, which are stacked along the crystallographic *b* axis with a displacement of 7.58(6) Å, producing a higher density structure (Fig. 4b) with one-dimensional square channels that are populated by a disordered array of water molecules. The amide bonds that connect the framework are oriented towards the centre of these hydrophilic channels (Fig. 4c). This uniaxial stacking produces a small distortion of the bond angles at the tetrahedral MATB unit, which lie in the range 103.4(9)–112.9(5)°.

### Sorption

The CO_2_ isotherms of CAF-1 synthesised at different temperatures and via the different routes described were measured as CAF-1 was found not to adsorb N_2_ at 77 K (Supplementary Methods). Type I isotherms^23^ (Supplementary Fig. 29–32) were displayed suggesting a microporous material with Brunauer–Emmett–Teller (BET) surface areas ranging from 77 to 288 m^2 ^g^−1^ compared to the surface area of PATnC of 125 m^2^ g^−1^ (Table 1). The measured surface area for the optimised synthesis is higher than that predicted based on the model (170 m^2 ^g^−1^ for a CO_2_ probe of 3.3 Å diameter) which could be attributed to the presence of defects within the structure. However there is a significant degree of hysteresis in the measured isotherm which is associated with swelling of the sorbent^23^. The swelling of CAF-1 upon the adsorption of CO_2_ was confirmed by analysing the PXRD pattern of CAF-1 with different amounts of CO_2_ adsorbed (Supplementary Fig. 33 and 34). This swelling helps explain how CAF-1 is able to accommodate more CO_2_ than predicted by the rigid model. Furthermore it was found that reaction temperature has a major effect on the porosity of CAF-1 with higher reaction temperatures being associated with higher porosities (Table 1). In contrast the starting material used to make CAF-1 has little effect on porosity. Similarly CAF-2 displays a Type I isotherm (Supplementary Fig. 31) indicating a microporous material with a BET surface area of 354 m^2^ g^-1^ compared to the surface area of PATCnC of 226 m^2^ g^−1^. Like CAF-1, the isotherm of CAF-2 also shows significant hysteresis suggesting swelling of the network.

**Table 1:** Surface area measurements

Material	Synthesis temperature (°C)	Starting material	BET surface area (m^2 ^g^−1^)	Pore volume (cm^3^ g^−1^)	Synthesis yield %
CAF-1	190	PATnC	77	0.033	87
CAF-1	250	PATnC	256	0.167	97
CAF-1	290	PATnC	288	0.232	32
CAF-1	250	Salt	246	0.169	88
CAF-1	250	Mixture	246	0.164	76
PATnC	0	Acid Chloride	125	0.081	93
CAF-2	240	PATCnC	354	0.245	97
PATCnC	0	Acid Chloride	226	0.169	67

Surface areas were calculated from CO_2_ isotherms at 195 K between p/p^0^ = 0.0 to 0.5. Pore volumes were calculated from the total volume of adsorbed gas once the sample had reached saturation. Example isotherms for each material can be found in Supplementary Fig. 29–32

### Stability of amide frameworks

**Fig. 5:**
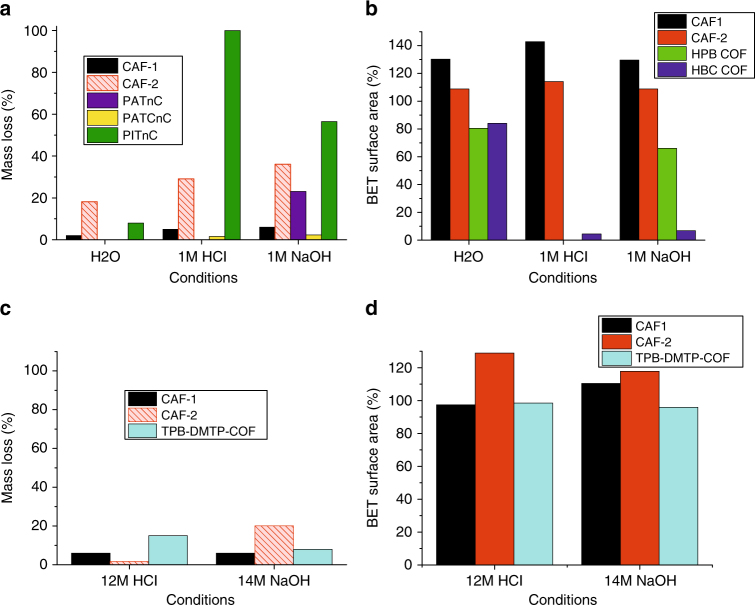
Hydrolytic Stability Testing. **a** Mass losses of amide networks CAF-1, CAF-2, PATnC, PATCnC and imine network PITnC after suspension in water, 1 M HCl_(aq)_ and 1 M NaOH_(aq)_ for 24 h at 100 °C^25^ (Supplementary Note 3): due to particle size it was not possible to filter CAF-2 and thus centrifuging/decanting was used for the washing procedure, resulting in mechanical losses. The values shown for CAF-2 are thus an upper limit for mass loss here and in **c** below. **b** Comparison of the residual BET surface areas (defined as a percentage of the as-made surface area) of CAF-1 and CAF-2 after suspension in water, 1 M HCl_(aq)_ and 1 M NaOH_(aq)_ for 24 h at 100 °C with HPB COF and HBC COF^25^, the most stable previously reported COFs under these conditions. **c**, **d** Comparison of the mass losses and residual BET surface areas of CAF-1 and CAF-2 after suspension in 12 M HCl_(aq)_ and 14 M NaOH_(aq)_ for 1 week at room temperature with those reported for TPB-DMTP-COF^24^, the most stable COF previously reported under these conditions (Supplementary Note 4). The mass losses shown for CAF-2 are an upper bound, as discussed for **a** above

The hydrolytic stability of CAF-1 and CAF-2 was investigated by suspending them in water, 1 M HCl or 1 M NaOH for 24 h at 100 °C (Supplementary Note 6); or by suspension in 12 M HCl or 14 M NaOH for 1 week at room temperature^24^ (Supplementary Note 7), these conditions were chosen to directly compare CAF-1 and CAF-2 with state-of-the-art stable imine COFs in the literature: these imine materials are highly chemically tailored to enhance their stability over that found for typical imines. The stability was assessed by a combination of mass loss, residual BET surface area, PXRD and changes in CHN microanalysis. Under all conditions CAF-1 and CAF-2 retained their PXRD patterns. In boiling conditions both CAF-1 and CAF-2 outperform the current most stable imine COFs^25^ (HPB COF and HBC COF, Fig. 5b) in terms of residual BET surface area. CAF-1 and PATnC are considerably more stable than the amorphous imine, PITnC synthesised as a typical two-dimensional imine analogue of the amides studied here, using the same organic cores but with imine rather than amide network-forming bonds and without chemical tailoring of imine stability (Supplementary Fig. 35, Fig. 5a): for example, PITnC dissolves completely in 1 M HCl. The small particle size of CAF-2 required centrifugation and decanting rather than filtration during workup, introducing mechanical losses not incurred for other materials. The mass losses reported for CAF-2 are thus upper limits. CAF-2 still shows smaller mass losses than the general imine PITnC, reinforcing the extra stability offered by the amide bond. Residual BET surface area, PXRD and CHN microanalysis, all also indicate that CAF-2 is stable under these conditions.

Under concentrated acid or base conditions, CAF-1 outperforms the current most stable imine COF in the literature^24^ (TPB-DMTP-COF, Fig. 5c and d), CAF-2 also affords the good stability expected for amides, outperforming TPB-DMTP-COF under acid conditions but not under basic conditions. CAF-1 and CAF-2, have superior residual BET surface area performance to imine COFs post-synthetically modified to amides^10^ (Supplementary Fig. 42). The analytical, surface area, mass loss and diffraction stability data demonstrates the stability of amide as a network-forming bond. A range of stabilities are to be expected for CAFs, much like other families of COF such as imines^24, 25^ (Supplementary Data 1), dependent on the specific chemistries and structures of the networks in question. CAF-1 is more stable than the most stable tailored imine COFs yet reported^24–29^, despite being based on simple organic constituents.

The thermal stability of CAF-1 was assessed by thermogravimetric analysis (TGA) in flowing air for up to 3 days isothermally at 100, 150 and 200 °C (Supplementary Fig. 44). The samples were then analysed with PXRD (Supplementary Fig. 45) and CHN (Supplementary Table 14) to assess structural and chemical stability. TGA traces at 100 and 150 °C show no weight loss after an initial rapid loss of adsorbed water. However, the TGA trace at 200 °C showed a steady weight loss after the initial loss of water with the sample losing 2.6 % of its dry mass in 3 days. The PXRD patterns showed no noticeable change after isothermal treatment at 100, 150 and 200 °C. CHN analysis shows that CAF-1 is highly stable in an oxygen containing atmosphere up to 150 °C after which point it starts to slowly degrade. Standard ramping TGA analysis shows both CAF-1 and CAF-2 are stable under an air atmosphere up to 300 °C (Supplementary Fig. 48 and 53) which is comparable to the best current examples of imine networks^28^.

## Discussion

The assembly of crystalline networks from irreversibly-formed strong bonds, exemplified here by amide, is desirable due to their stability but challenging because of the resulting difficulties in error-checking during synthesis that hinder the development of the required long-range positional order. Amides are an important materials class which have wide use throughout society and industry (e.g., Kevlar, Nylon), reflecting their thermal and hydrolytic stability coupled with structural and chemical diversity.

Using a devitrification approach, borrowed conceptually from the synthesis of crystalline silicas, we accessed reversible formation of the amide bond under strongly hydrolytic high temperature and pressure conditions that transformed an amorphous precursor network into the two-dimensional porous covalent amide framework CAF-1. The synthesis of a highly crystalline three-dimensional COF, CAF-2, demonstrates the potential of the approach to generate a range of covalent amide frameworks where the entire spatial order is conferred by the strong covalent bond arrangement. Both materials display the hydrolytic and thermal stability expected for amides, with CAF-1 being the most stable COF yet reported. These results suggest that other stable network-forming chemistries could yield crystalline systems if reversible conditions^30^ that permit the required error-checking can be identified.

## Methods

### Synthesis of PATnC

A solution of trimesoyl chloride (6.40 g, 24.11 mmol) in dry DMF (30 mL) was added drop wise to a solution of *trans*-1,4-cyclohexanediamine (4.50 g, 39.42 mmol) and triethyl amine (12 mL, 79.06 mmol) in dry DMF (500 mL) at 0 °C with strong stirring over the course of 1 h under N_2_. The suspension was allowed to warm to room temperature and stirred overnight followed by quenching with water (500 mL). The solids were filtered, dried *in vacuo*, ground into a coarse powder and then ball milled (15 min forward, 10 min rest, 15 min reverse, 350 rpm, 2 repetitions) into a fine powder. This powder was suspended in water (250 mL) and stirred overnight. The solid was filtered and the filtrate found to be pH neutral. The solid was dried by filtration and then activated at 110 °C under vacuum (10^−1^ mbar) overnight to yield PATnC as a fine very pale yellow powder (7.36 g, 22.5 mmol, 93.2 %).

### Synthesis of CAF-1 via PATnC

A Pyrex tube (OD 9.7 mm, ID 5.3 mm) was charged with dry PATnC (100 mg, 0.31 mmol, dried under 10^−3^ mbar at 120 °C overnight) and water (50 μL, 2.78 mmol). The water was frozen by submerging the bottom of the tube in liquid N_2_ and the tube was evacuated to 10^−4^ mbar and sealed with an approximate length of 10 cm. The tube was placed in a 300 mL Parr pressure reactor with 30 mL of water which was then sealed. The reactor was placed in an oven heating to 250 °C at 10 °Cmin^−1^ where it was held for 3 days followed by cooling at the same rate back to room temperature. The reactor and tube were opened and the solid was washed with DMF (3 × 10 mL), water (3 × 10 mL) and MeOH (3 × 10 mL) then dried by filtration yielding CAF-1 as a very pale yellow powder (99.1 mg, 0.30 mmol, 99.1 %).

### Synthesis of PATCnC

4,4′,4″,4‴-methanetetrayltetrabenzoic acid (4.00 g, 8.06 mmol) was refluxed in thionyl chloride (30 mL, 0.41 mol) at 70 °C for 3 h under N_2_ until the suspension had become a dark red/brown solution. The excess thionyl chloride was removed *in vacuo* yielding 4,4′,4″,4”’-methanetetraaryltetrabenzoyl chloride which was used without further purification. 4,4′,4″,4”’-methanetetraaryltetrabenzoyl chloride was dissolved in dry DMF (60 mL) and added drop wise with strong stirring to a solution of *trans*-1,4-cyclohexanediamine (2.00 g, 17.5 mmol) and triethyl amine (5.00 mL, 32.9 mmol) in dry DMF (750 mL) over 1 h at 0 °C under N_2_. The suspension was allowed to warm to room temperature and was stirred overnight followed by quenching with water (500 mL). The solids were filtered and washed with DMF (3 × 250 mL). The solids were dried overnight *in vacuo*, ground into a fine powder, suspended in water (250 mL) and stirred for 1 h. The solid was filtered and washed with water until the pH of the filtrate was neutral (3 × 250 mL) followed by MeOH (250 mL) and dried by filtration. The solid was activated at 120 °C under vacuum (10^−1^ mabr) overnight to yield PATCnC as a pale yellow powder (3.51×*g*, 5.37 mmol, 66.7 % over two steps).

### Synthesis of CAF-2

A Pyrex tube (OD 9.7 mm, ID 5.3 mm) was charged with dry PATCnC (100 mg, 0.15 mmol, dried under 10^−3^ mbar at 120 °C overnight) and water (40 μL, 2.22 mmol). The water was frozen by submerging the bottom of the tube in liquid N_2_ and the tube was evacuated to 10^−4^ mbar and sealed with an approximate length of 10 cm. The tube was placed in a 300 mL Parr pressure reactor with 30 mL of water which was then sealed. The reactor was placed in an oven heating to 240 °C at 10 °C min^−1^ where it was held for 7 days followed by cooling at 0.1 °Cmin^−1^ back to room temperature. The reactor and tube were opened and the solid was washed with DMF (10 × 10 mL), water (10 × 10 mL) and MeOH (10 × 10 mL) then dried by filtration yielding CAF-2 as a grey powder (90.55 mg, 0.14 mmol, 92.5 %).

### Data availability

The data that support the findings of this study are available in the Research Data Catalogue of the University of Liverpool with the identifier doi:10.17638/datacat.liverpool.ac.uk/386

## Electronic supplementary material

Supplementary Information

Description of Additional Supplementary Files

Supplementary Data 1
