# Supplementary material for: Stable and ordered amide frameworks synthesised under reversible conditions which facilitate error checking
Source: Nat Commun. 2017 Oct 24;8:1102. doi: 10.1038/s41467-017-01423-5 (PMC5654755; doi:10.1038/s41467-017-01423-5)
Supplement: Supplementary file 1 — Supplementary Information [file 41467_2017_1423_MOESM1_ESM.pdf]

Supplementary Methods

Synthesis of polyamide(1,3,5-tricarboxybenzene-net-trans-1,4-cyclohexanediamine, **PATnC**

A solution of trimesoyl chloride (6.40 g, 24.11 mmol) in dry DMF (30 mL) was added drop wise to a solution of *trans*-1,4-cyclohexanediamine (4.50 g, 39.42 mmol) and triethyl amine (12 mL, 79.06 mmol) in dry DMF (500 mL) at 0 °C with strong stirring over the course of 1 hour under N_2. The suspension was allowed to warm to room temperature and stirred overnight followed by quenching with water (500 mL). The solids were filtered, dried *in vacuo*, ground into a coarse powder and then ball milled (15 min forward, 10 min rest, 15 min reverse, 350 rpm, 2 repetitions) into a fine powder. This powder was suspended in water (250 mL) and stirred overnight. The solid was filtered and the filtrate found to be pH neutral. The solid was dried by filtration and then activated at 110 °C under vacuum (10^{-1} mbar) overnight to yield **PATnC** as a fine very pale yellow powder (7.36 g, 22.5 mmol, 93.2 %).

IR (ATR, cm^{-1}): 3270, 3072, 2937, 2861, 1706, 1631, 1525; CHN (calcd., found for $C_{18}H_{21}N_3O_3.0.8H_2O$): C (63.25, 63.16), H (6.66, 6.79), N (12.29, 12.52); ^{13}C CP MAS NMR (ppm): 167.3, 135.0, 129.8, 49.2, 31.1, 24.6; ^{15}N CP MAS NMR (ppm): 128.

Synthesis of **CAF-1** *via* **PATnC**

A Pyrex tube (OD 9.7 mm, ID 5.3 mm) was charged with dry **PATnC** (100 mg, 0.31 mmol, dried under 10^{-3} mbar at 120 °C overnight) and water (50 µL, 2.78 mmol). The water was frozen by submerging the bottom of the tube in liquid N_2 and the tube was evacuated to 10^{-4} mbar and sealed with an approximate length of 10 cm. The tube was placed in a 300 mL Parr pressure reactor with 30 mL of water which was then sealed. The reactor was placed in an oven heating to 250 °C at 10 $°Cmin^{-1}$ where it was held for 3 days followed by cooling at the same rate back to room temperature. The reactor and tube were opened and the solid was washed with DMF (3 x 10 mL), water (3 x 10 mL) and MeOH (3 x 10 mL) then dried by filtration yielding **CAF-1** as a very pale yellow powder (99.1 mg, 0.30 mmol, 99.1 %).

IR (ATR): 3232, 3060, 2932, 2858, 1628, 1529 cm^{-1}; CHN (calcd., found for $C_{18}H_{21}N_3O_3.1.30H_2O$): C (61.63, 61.63), H (6.78, 6.70), N (11.98, 11.91); ^{13}C CP MAS NMR (ppm): 167.9, 136.1, 127.8, 48.4, 31.2, 25.1; ^{15}N CP MAS NMR (ppm): 130.

Synthesis of polyamide(4,4',4'',4'''-tetra(carboxyphenyl)methane-net-trans-1,4-cyclohexanediamine), **PATCnC**

4,4',4'',4'''-methanetetrayltetrabenzoic acid (4.00 g, 8.06 mmol) was refluxed in thionyl chloride (30 mL, 0.41 mol) at 70 °C for 3 hours under N_2 until the suspension had become a dark red/brown solution. The excess thionyl chloride was removed *in vacuo* yielding 4,4',4'',4'''-methanetetraaryltetrabenzoyl chloride which was used without further purification. 4,4',4'',4'''-methanetetraaryltetrabenzoyl chloride was dissolved in dry DMF (60 mL) and added drop wise with strong stirring to a solution of *trans*-1,4-cyclohexanediamine (2.00 g, 17.5 mmol) and triethyl amine (5.00 mL, 32.9 mmol) in dry DMF (750mL) over 1 hour at 0 °C under N_2. The suspension was allowed to warm to room temperature and was stirred overnight followed by quenching with water (500 mL). The solids were filtered and washed with DMF (3 x 250 mL). The solids were dried overnight *in vacuo*, ground into a fine powder, suspended in water (250 mL) and stirred for 1 hour. The solid was filtered

and washed with water until the pH of the filtrate was neutral (3 x 250 mL) followed by MeOH (250 mL) and dried by filtration. The solid was activated at 120 °C under vacuum (10^{-1} mabr) overnight to yield **PATCnC** as a pale yellow powder (3.51 g, 5.37 mmol, 66.7 % over two steps).

IR (ATR): 3290, 3051, 2931, 2858, 1706, 1639, 1605, 1528 cm^{-1}; CHN (cald., found for $C_{41}H_{40}N_4O_4.2.70H_2O$); C (70.21, 70.45), H (6.52, 6.00), N (7.99, 7.47); ^{13}C CP MAS NMR (ppm): 172.5, 167.7, 149.0, 132.7, 128.9, 65.1, 49.0, 31.1, 24.7; ^{15}N CP MAS NMR (ppm): 129, 45.

Synthesis of **CAF-2**

A Pyrex tube (OD 9.7 mm, ID 5.3 mm) was charged with dry **PATCnC** (100 mg, 0.15 mmol, dried under 10^{-3} mbar at 120 °C overnight) and water (40 µL, 2.22 mmol). The water was frozen by submerging the bottom of the tube in liquid N_2 and the tube was evacuated to 10^{-4} mbar and sealed with an approximate length of 10 cm. The tube was placed in a 300 mL Parr pressure reactor with 30 mL of water which was then sealed. The reactor was placed in an oven heating to 240 °C at 10 $°Cmin^{-1}$ where it was held for 7 days followed by cooling at 0.1 $°Cmin^{-1}$ back to room temperature. The reactor and tube were opened and the solid was washed with DMF (10 x 10 mL), water (10 x 10 mL) and MeOH (10 x 10 mL) then dried by filtration yielding **CAF-2** as a grey powder (90.55 mg, 0.14 mmol, 92.5 %).

IR (ATR): 3401, 3267, 3059, 2937, 2863, 1610, 1539 cm^{-1}; CHN (calcd., found for $C_{41}H_{40}N_4O_4.11.55H_2O$): C (57.20, 58.01), H (7.39, 6.59), N (6.51, 5.66); ^{13}C CP MAS NMR (ppm): 173.9, 168.6, 149.3, 134.4, 132.5, 128.7, 126.1, 64.4, 47.9, 31.4, 25.4; ^{15}N CP MAS NMR (ppm): 135, 45.

Synthesis of di-(1,3,5-benzenetricarboxylate)-tri-(trans-1,-4-cyclohexanediamonium)-tetrahydrate, **1**

Trimesic acid (2.10 g, 10 mmol) and *trans*-1,4-cyclohexanediamine (1.71 g, 15 mmol) were dissolved in an EtOH/water mixture (1:1 v/v, 100 mL) by refluxing. Once all the solids had dissolved the solution was allowed to cool to RT followed by the addition of isopropyl alcohol (100 mL) resulting in the precipitation of a white crystalline solid. This solid was filtered and recrystallized from the minimum amount of water/EtOH (1:3 v/v) and the solid filtered and washed with ethanol to yield **1** as a white crystalline solid (2.76 g, 3.3 mmol, 66.1 %).

Synthesis of **CAF-1** *via* **1**

A Pyrex tube (OD 9.7 mm, ID 5.3 mm) was charged with dry **1** (127 mg, 0.30 mmol) and water (22 µL, 1.2 mmol). The water was frozen by submerging the bottom of the tube in liquid N_2 and the tube was evacuated to 10^{-4} mbar and sealed with an approximate length of 10 cm. The tube was placed in a 300 mL Parr pressure reactor with 30 mL of water which was then sealed. The reactor was placed in an oven heating to 250 °C at 10 °C min^{-1} where it was held for 3 days followed by cooling at the same rate back to room temperature. The reactor and tube were opened and the solid was washed with DMF (3 x 10 mL), water (3 x 10 mL) and MeOH (3 x 10 mL) then dried by filtration yielding **CAF-1** as a very pale yellow powder (88.1 mg, 0.27 mmol, 89.7 %).

Synthesis of **CAF-1** *via a physical mixture of starting materials*

Trimesic acid (64.0 mg, 0.305 mmol) and *trans*-1,4-cyclohexanediamine (52 mg, 0.458 mmol) were ground together into a homogenous powder in a mortar and pestle. The resultant solid was loaded into a Pyrex tube (OD 9.7 mm, ID 5.3 mm) with water (34 µL, 1.85 mmol). The water was frozen by

submerging the bottom of the tube in liquid N_2 and the tube was evacuated to 10^{-4} mbar and sealed with an approximate length of 10 cm. The tube was placed in a 300 mL Parr pressure reactor with 30 mL of water and sealed. The reactor was placed in an oven heating to 250 °C at 10 °C min^{-1} where it was held for 3 days followed by cooling at the same rate back to room temperature. The reactor and tube were opened and the solid was washed with DMF (3 x 10 mL), water (3 x 10 mL) and MeOH (3 x 10 mL) then dried by filtration yielding **CAF-1** as a very pale yellow powder (75.9 mg, 0.23 mmol, 76.0 %).

Synthesis of Polyimine(Benzene-1,3,5-tricarboxaldehyde-net-trans-1,4-cyclohexyldiamine), **PITnC**

Benzene-1,3,5-tricarbaldehyde (162 mg, 1 mmol) and and *tans*-1,4-cyclohexanediamine (194 mg, 1.7 mmol) were dissolved in 1,4-dioxane (25 mL) and acetic $acid_{(aq)}$ (3M, 2 mL) was added causing an immediate white precipitate to form. The suspension was heated to 101 °C and refluxed for 3 hours. The reaction was monitored by TLC and was ended one all the aldehyde had been consumed. The suspension was allowed to cool and the solids were filtered, washed with 1,4-dioxane (50 mL) followed by THF (50 mL) and dried *in vacuo* overnight. The solid was activated at 100 °C at 10^{-3} mbar for 3 days to yield the desolvated **PITnC** as a white solid (0.26 g, 0.93 mmol, 93 %).

IR (ATR): 3404, 2931, 2857, 1700, 1641, 1600, 1489, 1380 cm^{-1}; CHN (calcd., found for $C_{18}H_{21}N_3.1.1H_2O$): C (72.26, 71.80), H (7.82, 7.93), N (14.04, 14.06).

Solid state NMR spectroscopy. All solid-state NMR spectra were measured on a 9.4 T Bruker Avance III HD NMR spectrometer equipped with a 4 mm HXY triple resonance MAS probe (in double resonance mode) operating at a ^{1}H Larmor frequency of 400.13 MHz and with the X channel tuned to ^{13}C or ^{15}N at 100.63 and 40.55 MHz, respectively. All NMR spectra were obtained with cross polarization (CP) and at Magic Angle Spinning (MAS) frequencies of 12.5 kHz and 8 kHz for ^{13}C and ^{15}N, respectively. ^{1}H pulses and SPINAL-64 heteronuclear decoupling[1] were performed at a radio-frequency (rf) field amplitude of 83 kHz. ^{13}C CP MAS experiments were obtained with contact times of 2 ms and with a ^{13}C rf field of 62.5 kHz, while the ^{1}H rf field amplitude was ramped to obtain maximum signal at a ^{1}H rf field of approximately 60 kHz (as optimized on the sample directly). ^{15}N CP MAS experiments were obtained with contact times of 2 ms and with a ^{15}N rf field of 42 kHz, while the ^{1}H rf field amplitude was ramped to obtain maximum signal at a ^{1}H rf field of approximately 50 kHz (optimized on glycine). ^{13}C and ^{15}N chemical shifts (± 0.5 and ± 0.2 ppm for the amorphous PATnC/PATCnC and crystalline **CAF-1**/**CAF-2**, respectively) were externally referenced to the CH group of adamantane at 29.45 ppm[22] and the nitrogen resonance of glycine at 33.4 ppm[3]. All samples were packed in air.

NMR spectroscopy. Solution phase ^{1}H NMR spectroscopy was carried out using a Bruker AVANCE-400 MHz NMR spectrometer using the residual protonated solvent resonance as an internal standard.

Surface Area Measurements. **CAF-1** was found not to absorb N_2 at 77 K. The lack of measured N_2 porosity is likely due to restricted diffusion which is a well-known problem for microporous materials with pore widths <0.45 nm[4] (**CAF-1** pore width <0.36 nm). As such all materials surface areas were analyzed using CO_2 at 195 K to aid comparison. This hypothesis is supported by the observation that acquisition of CO_2 isotherms at 195 K took around 1 week to complete for **CAF-1** samples. Surface area measurements were performed using a Micrometrics 3Flex Surface Characterization Analyzer with CO_2 as a probe between 0.5 and 1000 mbar at 195 K maintained by a Sumitomo CH-104 Cryostat. Samples

of approximately 100 mg were activated at between 100 and 120 °C overnight at < 10^{-4} mbar prior to analysis.

Thermal analysis. Thermal Gravimetric Analysis (TGA) was carried out on a TA Instruments Q600 between 25 and 800 °C with a 10 °Cmin^{-1} scan rate and a gas flow (N_2 or Air) of 100 mLmin^{-1}.

CHN microanalysis. Carbon, Nitrogen and Hydrogen content were determined by microanalytical procedures using a Thermo Flash EA1112 CHNS-O Analyser.

Synchrotron PXRD. Synchrotron X-ray powder diffraction data were collected on the I11 beam line at Diamond Light Source, Oxfordshire[5] in 0.7 mm diameter borosilicate capillaries at 298 K. Data were collected using the Position Sensitive Detector (PSD, Mythen-2) in order to minimise data collection time and thus minimise exposure to the X-ray beam and any resulting beam damage.

Synchrotron PDF. Room temperature pair distribution function (PDF) data were collected on the I15 beamline at Diamond Light Source, Oxfordshire, at a wavelength of 0.1620 Å. Samples were sealed inside 0.7 mm diameter borosilicate capillaries. The scattering data ($0.5 \leq Q \leq 20$ Å^{-1}) were processed into PDF data using the program GudrunX[6]. PDF data were analysed using the computer program Topas[7]. The crystal structure refinement was performed using a starting model produced by DFT methods. The unit cell and atomic parameters were refined; dampening was used to prevent large atomic movements in the early stages of the refinement. The $D(r)$ PDF data was scaled as a function of distance, r, using an exponential function to model the effect of finite instrumental Q resolution[8] and a spherical correction function, $f_{\text{sphere}}(r)$, to model a finite ordered sphere with a refined radius[9].

In situ crystallographic studies of CO_2 adsorption on **CAF-1.** In order to confirm that **CAF-1** swells upon the adsorption of CO_2 a sample of **CAF-1** was loaded into a gas cell at the I11 beam of Diamond Light Source and activated at 393 K under dynamic vacuum. The sample was then cooled under vacuum to 195 K and a pattern measured. Next the sample was dosed with approximately 0.010 bar of CO_2, the pressure allowed to equilibrate and another pattern collected. This process was repeated for 0.018, 0.071, 0.342, 0.746 and 1.529 bar giving an approximate adsorption isotherm with a PXRD pattern collected at each point. The collected PXRD patterns were then subjected to a Pawley fitting using the DFT-determined **CAF-1** structure to extract lattice parameters for each point on the isotherm.

Lab PXRD. Lab X-ray powder diffraction data were collected on a Bruker D8 Advance powder X-ray diffractometer at 298 K in transmission geometry with Cu Kα_1 radiation (λ = 1.54060 Å). Samples were ground into fine powders and loaded into 0.7 mm internal diameter borosilicate capillaries. Typical scans were collected as step scans over 1 h between 3 and 50 °2θ.

CAF-1 *relative crystallinity quantification method*. Approximately 8 mg of **CAF-1** and 2 mg of adamantane were ground together into a fine homogeneous powder for each sample to be measured. 0.7 mm internal diameter borosilicate capillaries were loaded with these powder samples and measured on a lab X-ray diffractometer using Cu Kα_1 radiation for 1 hour between 3 and 35 °2θ. The maximum intensity of the **CAF-1** 2-10 reflection was divided by the amount of **CAF-1** used to make the sample in mmols to give a value in counts/mmol thus normalising the value to the amount of **CAF-1** used. The maximum intensity of the 4 adamantane reflections were divided by the amount of adamantane used to make the sample in mmols to give a value in counts/mmol for each reflection thus normalising the values to the amount of adamantane used. The value for the **CAF-1** 2-10

reflection in counts/mmol was then divided by the values for the 101, 111, 211 and 202 adamantane reflections in counts/mmol to give a relative crystallinity for each adamantane refection for each sample, giving four series, the 101, 111, 211 and 202 series. The four series of relative crystallinity experiments showed the same trends indicating that the method is internally consistent. Each sample was synthesized in triplicate and the analysis conducted separately for each repeat to ensure reproducibility. This method was used to assess the relative crystallinity of **CAF-1** samples produced via different methods and under different reaction conditions.

FT-IR spectroscopy. Infrared spectra were collected from finely ground powdered samples between 4000 and 650 cm^{-1} using a Perkin Elmer Spectrum 100 instrument in Attenuated Total Reflectance (ATR) mode with a resolution of 4 cm^{-1}, 4 scans were collected per sample after the collection of a background.

Computational Details. Plane-wave based density functional theory (DFT) calculations were performed using the VASP[10] and CASTEP[11] programmes.

Unit cell parameters and atomic positions were optimised using VASP with the optB86b-vdW density functional[12] which provides a more accurate treatment of van der Waals interactions than standard semi-local functionals. Core electrons were treated using the projector augmented wave method[13]. A plane-wave cutoff energy of 520 eV was used, and *k*-point grids were chosen such that the number of *k*-points in any direction multiplied by the length of cell in that direction was greater than 40 Å. Forces were minimised until they reached less than 0.001 eV/Å. The symmetry of optimised structures was determined using the Findsym programme[14].

NMR parameters were calculated with CASTEP using the GIPAW method[15] using the structures determined using VASP. The PBE functional[16] was used with a plane-wave cutoff energy of 600 eV and pseudopotentials generated on-the-fly by CASTEP. Computed ^{13}C isotropic shielding values σ_{iso} were converted to predicted δ_{iso} values with the equation $\delta_{iso} = 171-1.02\sigma_{iso}$[17], and simulated ^{13}C NMR spectra generated by using each C in the structural model to contribute one Gaussian to the spectrum centred at its calculated δ_{iso}.

Finite temperature Molecular Dynamics simulations were performed using tight binding DFT method using the DFTB+ package[18]. We used Slater-Koster parameter set mio-1-1 developed for organic molecules containing C, H, O and N atoms as well as S, and P[19] and combined it with the Slater-Kirkwood dispersion model that has been demonstrated to reproduce the structure of stacked complexes such DNA base pairs[20]. A unit cell of **CAF-1** containing the total of 297 atoms, including 9 water molecules distributed between the 6 triangular one-dimensional pores, was equilibrated for 20 ps at 300 K followed by a 50 ps production run. Velocity Verlet algorithm with time step of 0.5 fs and Berendsen thermostat with 50 fs time constant were used for these constant NVT simulations.

Supplementary Table 1. CP MAS NMR ^{13}C and ^{15}N chemical shifts and spectral assignments for **PATnC**, **CAF1**, **PATCnC** and **CAF2**.[a]

Compound	^{13}C δ_{iso} (ppm) [b]	^{13}C FWHM (Hz) [c]	^{13}C Assignments	^{15}N δ_{iso} (ppm) [d]	^{15}N FWHM (Hz) [c]	^{15}N Assignments
**PATnC**	167.3	730	***C***O			
	135.0	650	***C***$^{(IV)Ar}$			
	129.8	710	***C***H^{Ar}	128	640	***N***H
	49.2	590	***C***HNH[e]			
	31.1	470	***C***H_2			
	24.6	440				
**CAF1**	167.9	380	***C***O			
	136.1	340	***C***$^{(IV)Ar}$			
	127.8	560	***C***H^{Ar}	130	540	***N***H
	48.4	420	***C***HNH[e]			
	31.2	390	***C***H_2			
	25.1	410				
**PATCnC**	172.5	700	***C***OOH			
	167.7	660	***C***ONH	129	640	***N***H
	149.0	530	C(***C***$^{(IV)Ar}$)$_4$			
	132.7	460	***C***$^{(IV)}$CO			
	128.9	640	***C***HC$^{(IV)}$C$^{(IV)}$			
	65.1	180	***C***(C$^{(IV)Ar}$)$_4$			
	49.0	540	***C***HNH[e]	45	250	***N***H_2
	31.1	470	***C***H_2			
	24.7	490				
**CAF2**	173.9	240	***C***OOH			
	168.6	120	***C***ONH			
	149.3	180	C(***C***$^{(IV)Ar}$)$_4$	135	100	***N***H
	134.4	150	***C***HC$^{(IV)}$CO			
	132.5	180	***C***$^{(IV)}$CO			
	128.7	200	***C***HC$^{(IV)}$C$^{(IV)}$			
	126.1	120				
	64.4	100	***C***(C$^{(IV)Ar}$)$_4$	45	240	***N***H_2
	47.9	90	***C***HNH[e]			
	31.4	220	***C***H_2			
	25.4	250				

[a] ^{13}C and ^{15}N chemical shifts are given at ± 0.5 and ± 0.2 ppm for the amorphous **PATnC**/**PATCnC** and **CAF1**/**CAF2**. [b] ^{13}C chemical shifts are referenced to the CH resonance of adamantane at 29.45 ppm.[2] [c] Full Width at Half Maximum (FWHM) (± 10 Hz) obtained from spectral deconvolution. [d] ^{15}N chemical shifts are referenced to the nitrogen resonance of glycine at 33.4 ppm.[3] [e] The apparent multiplicity of the ***C***HNH carbon arises from residual ^{13}C – ^{14}N dipolar coupling not averaged out by MAS[21].

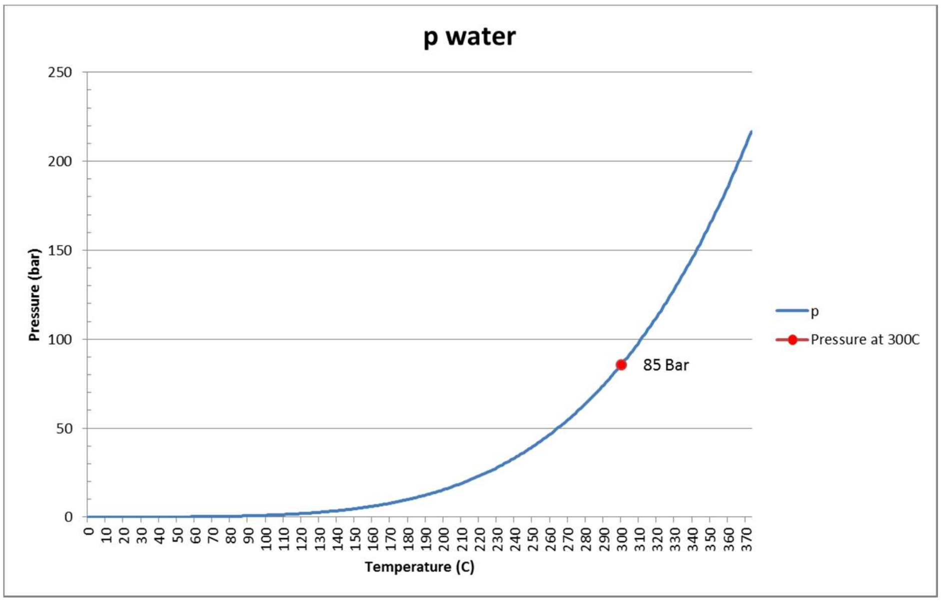

Supplementary Figure 1. Sealing the Pyrex tubes inside a Parr reactor with a small quantity of water (typically 30 mL for a 300 mL reactor) is crucial otherwise the tubes will fail due to the pressures generated by the small quantity of water at the reaction temperatures. A pressure of 85 bar is calculated to be generated at 300 °C for instance which was the upper range of our synthesis exploration. The water inside the reactor but outside the tubes will also generate a similar pressure and therefore by balancing the pressures inside and outside of the tubes failure of the tubes is avoided. It is not essential for this reaction to be carried out in sealed tubes at low initial pressures; it can indeed be achieved by sealing the polymer and water catalyst in a Parr reactor directly. However at the temperatures required to attain reversibility organic molecules are very susceptible to degradation particularly in the presence of air. When attempting to do this reaction directly in a Parr reactor it is very difficult to exclude all of the air and the products are often charred to some extent.

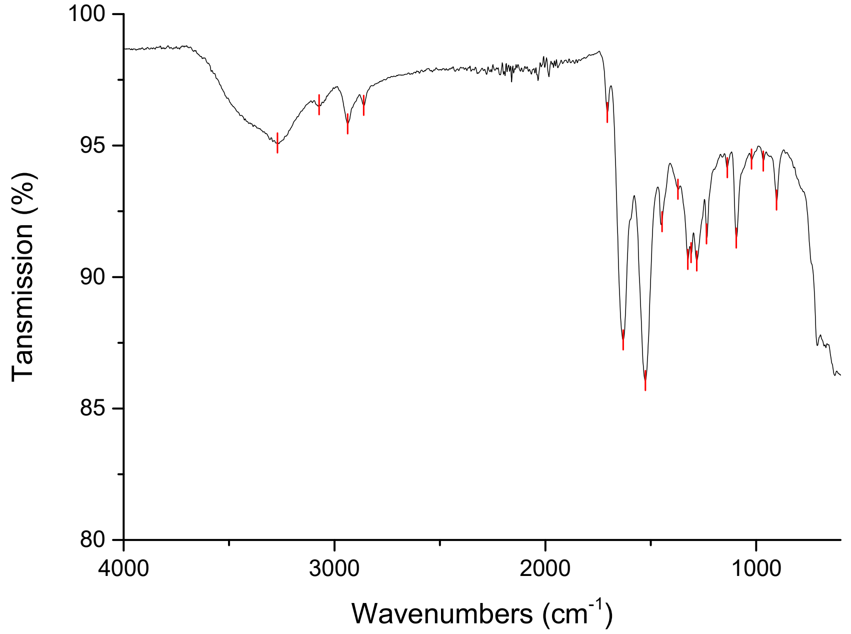

Supplementary Figure 2 FTIR spectrum of **PATnC**

Supplementary Table 2 FTIR peak table of **PATnC**

Peak Centre (cm^{-1})	Transmission (%)	Assignment
3270	95.1	N-H stretch
3073	96.6	C-H stretch
2938	95.8	C-H stretch
2862	96.5	C-H stretch
1707	96.3	C=O (COOH) stretch
1631	87.6	C=O (amide) stretch
1526	86.1	N-H bend
1447	92.1	
1371	93.3	
1325	90.7	
1309	90.9	
1282	90.6	
1236	91.6	
1137	94.2	
1095	91.5	
1022	94.5	
966	94.4	
903	92.9	

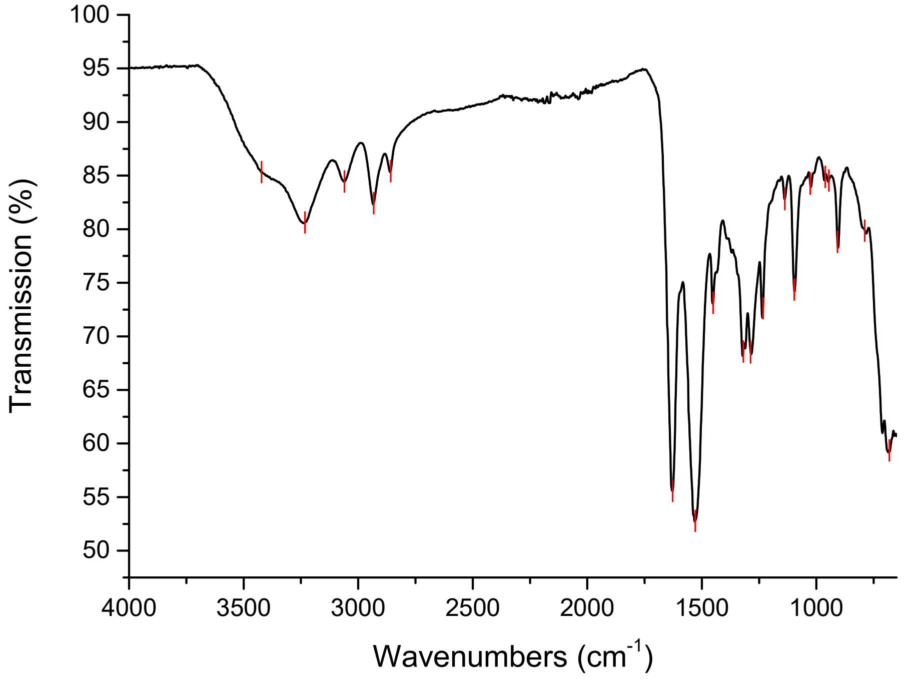

Supplementary Figure 3 FTIR spectrum of **CAF-1**

Supplementary Table 3 FTIR peak table of **CAF-1**

Peak Centre (cm^{-1})	Transmission (%)	Assignment
3422	85.3	N-H stretch
3232	80.6	N-H stretch
3060	84.4	C-H stretch
2932	82.4	C-H stretch
2858	85.4	C-H stretch
1628	55.6	C=O stretch
1529	52.8	C-N stretch
1451	73.1	
1319	68.6	
1287	68.5	
1233	72.6	
1138	82.8	
1097	74.4	
1027	84.2	
962	84.9	
945	84.5	
908	78.8	
789	79.9	
682	59.4	

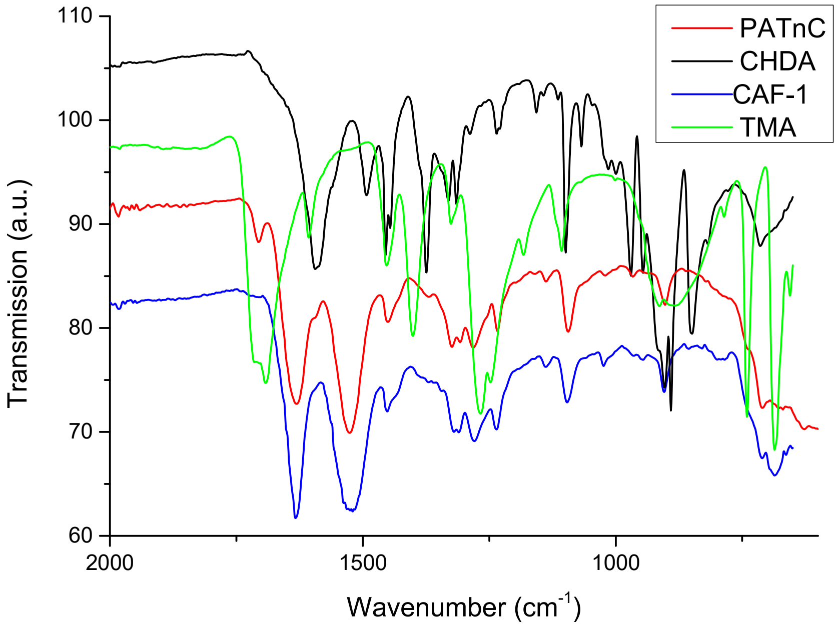

Supplementary Figure 4 Comparison of FTIR C=O stretch region for **CAF-1** and precursors

Supplementary Table 4 CHN analysis of ten **CAF-1** samples[a]

Sample	Measured			water equiv.	Calculated			Max Dif
	C	H	N		C	H	N	
**A**	60.70	6.66	11.69	1.55	60.85	6.84	11.83	0.18
**B**	61.17	6.62	11.74	1.40	61.31	6.80	11.92	0.18
**C**	61.86	6.69	11.88	1.25	61.79	6.77	12.01	0.13
**D**	61.92	6.72	11.97	1.20	61.95	6.67	12.04	0.07
**E**	60.49	6.68	11.78	1.65	60.54	6.86	11.77	0.18
**F**	60.82	6.68	11.66	1.55	60.85	6.84	11.83	0.17
**G**	60.67	6.58	11.67	1.55	60.85	6.84	11.83	0.26
**H**	61.37	6.67	11.80	1.40	61.31	6.80	11.92	0.13
**I**	61.63	6.70	11.91	1.30	61.63	6.78	11.98	0.08
**J**	60.79	6.68	11.72	1.55	60.85	6.84	11.83	0.16

[a] Each entry in the table above is for a separate sample of **CAF-1** synthesised in parallel according to the procedure for the synthesis of **CAF-1** via **PATnC** as described in the methods section (Supplementary Methods).

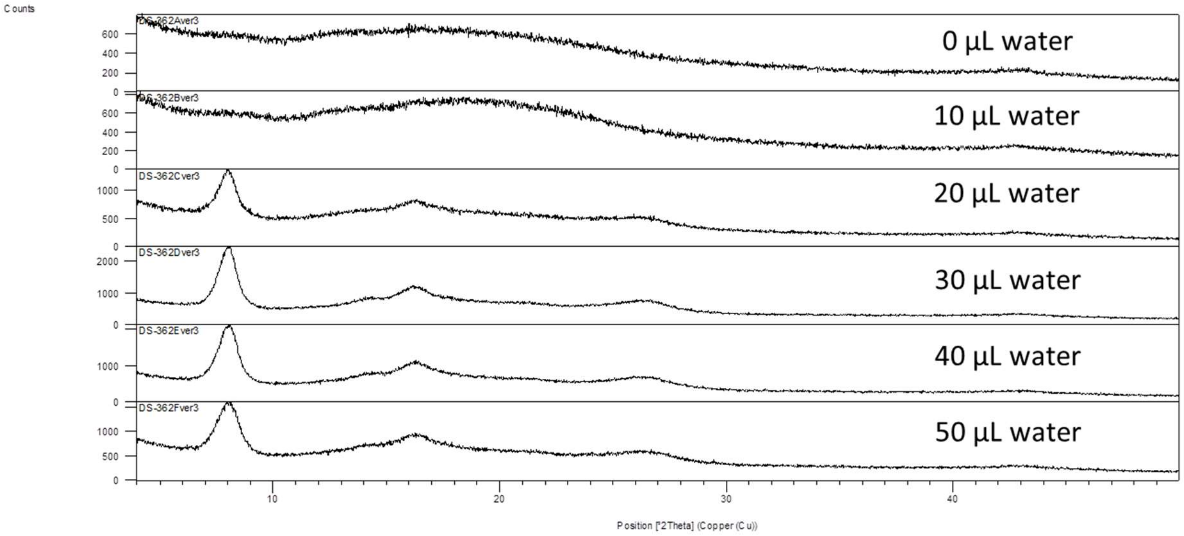

Supplementary Figure 5. PXRD patterns of variable water content series of CAF-1 synthesis. The patterns were collected using Cu Kα_1 radiation on a lab instrument in transmission foil geometry with a 1 hour collection time. Each sample was prepared using the standard **CAF-1** protocol from **PATnC** detailed in the methods section (Supplementary Methods) except that a 1 day reaction time was used and the volume of water added to the tubes was varied between 0 and 50 µL. There is no change in the PXRD pattern observed when between 0 and 10 µL of water are used however in cases when 20 µL or more of water were used **CAF-1** was formed.

Supplementary Note 1 Alternative synthesis of **CAF-1**

Having demonstrated a route to **CAF-1** using **PATnC** as the starting material, we considered other synthetic pathways. As it is postulated that the error-checking which enables the development of order in **CAF-1** is based around the breaking of amide linkages by water and their subsequent reforming via solid state polymerization (SSP), we considered whether it was possible to both form a polymer via SSP and then *in situ* devitrify it into **CAF-1**. Therefore a 1:1.5 molar ratio of TMA and CHDA was ground into a homogeneous powder and a salt of the same molar ratio was also prepared by refluxing a solution of TMA and CHDA in water/ethanol followed by precipitation with isopropyl alcohol (Supplementary Methods). Both were subjected to the same conditions as **PATnC** had been previously, taking care to account for the water of crystallization of the salt and the water which would be released by the salt and mixture upon amide formation so that the absolute molar quantity of free water inside the reaction vessel would be the same in all cases. In both cases **CAF-1** was formed as evidenced by PXRD (Supplementary Figure 8). The use of a physical mixture allows for the formation of a COF in one pot in a solvent free manner, other examples of solvent-free COF synthesis have been achieved by mechanochemical methods[22] based on imine bond-forming chemistry.

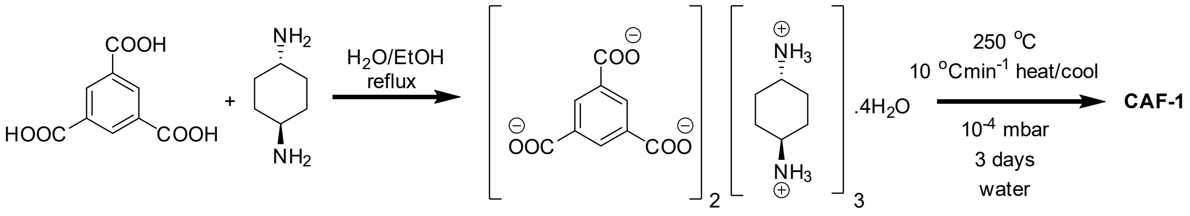

Supplementary Figures 6 Synthetic route to **CAF-1** from a salt

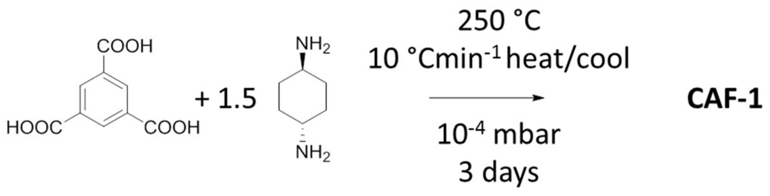

Supplementary Figure 7 Synthetic route to **CAF-1** directly from molecular starting materials

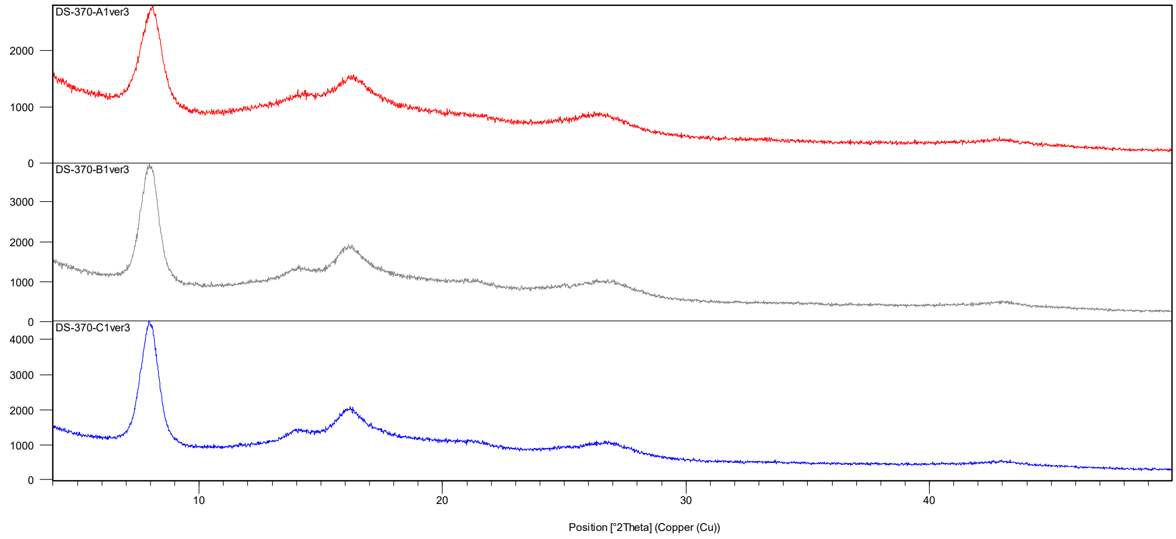

Supplementary Figure 8 PXRD patterns for **CAF-1** synthesis from different starting materials. The patterns were collected using Cu Kα_1 radiation on a lab instrument in transmission foil geometry with a 1 hour collection time. The top red pattern came from a material that was synthesized according to the procedure for the synthesis of **CAF-1** from **PATnC** as outlined in the Supplementary Methods section. The middle black pattern came from a material that was synthesized following the procedure for the synthesis of **CAF-1** from **1** (the salt) as outlined in the Supplementary Methods section. The bottom blue pattern came from a material that was synthesized following the procedure for the synthesis of **CAF-1** from a physical mixture of molecular starting materials as outlined in the Supplementary Methods section. In all cases regardless of starting material the **CAF-1** pattern is obtained indicating that all three routes successfully achieve **CAF-1**.

Supplementary Note 2 Optimization of **CAF-1** synthesis

The reaction parameters in the **PATnC** based route (Supplementary Methods) were optimized including the reaction time and temperature, and the amount of water added. Multiple series of reactions were prepared in which one of the parameters was systematically varied. The relative crystallinity of the samples was then assessed by using adamantane as an internal standard in PXRD experiments (Supplementary Figure 9). The various different routes to **CAF-1** were also compared in the same manner.

It was found that after 3 days a plateau is reached in terms of relative crystallinity after which no further improvement is observed and that at longer reaction times the amount of isolated product began to fall (Supplementary Figure 10). The optimum synthesis temperature was found to be 250 °C, where a maximum in relative crystallinity and in the amount of isolated product was achieved (Supplementary Figure 11).

Finally it was determined that synthesis from a physical mixture of the molecular starting materials affords the most crystalline CAFs, followed by the salt and finally **PATnC** (Supplementary Figure 12) with the amount of isolated product showing the opposite trend. A plausible explanation for this trend is that the monomers will be able to reorient more freely thus facilitating crystallization when using molecular or salt starting materials. The polymer starts with the correct molecular connectivity but this connectivity readily forms locally anyway in the other routes, and the components are much more constrained by the pre-formation of the amorphous network.

The effect of the amount of water added to each tube was investigated but no consistent trends were observed. It is likely that a combination of complex variables is at play, the exact internal volume of the tube and the precise amount of water which remains in the tube after sealing are difficult to control and may be the cause of the lack of any clear trend.

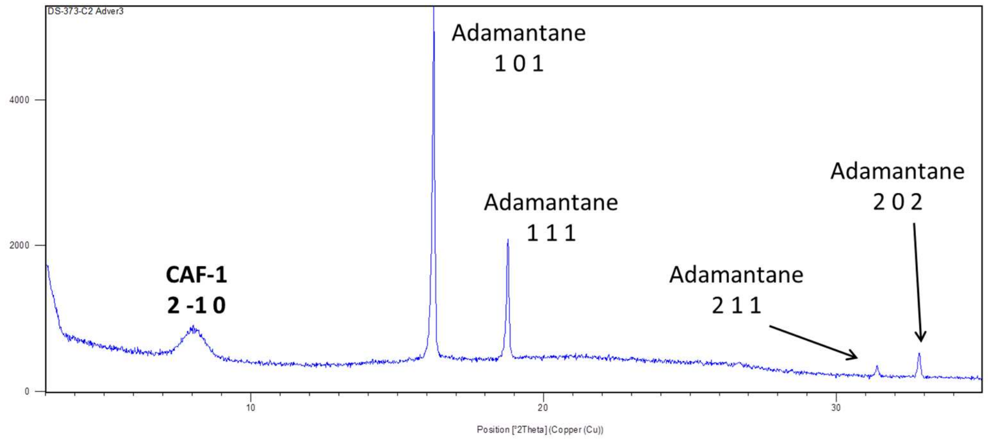

Supplementary Figure 9. PXRD pattern of a representative **CAF-1**/adamantane sample that was used for the quantification of the relative crystallinity.

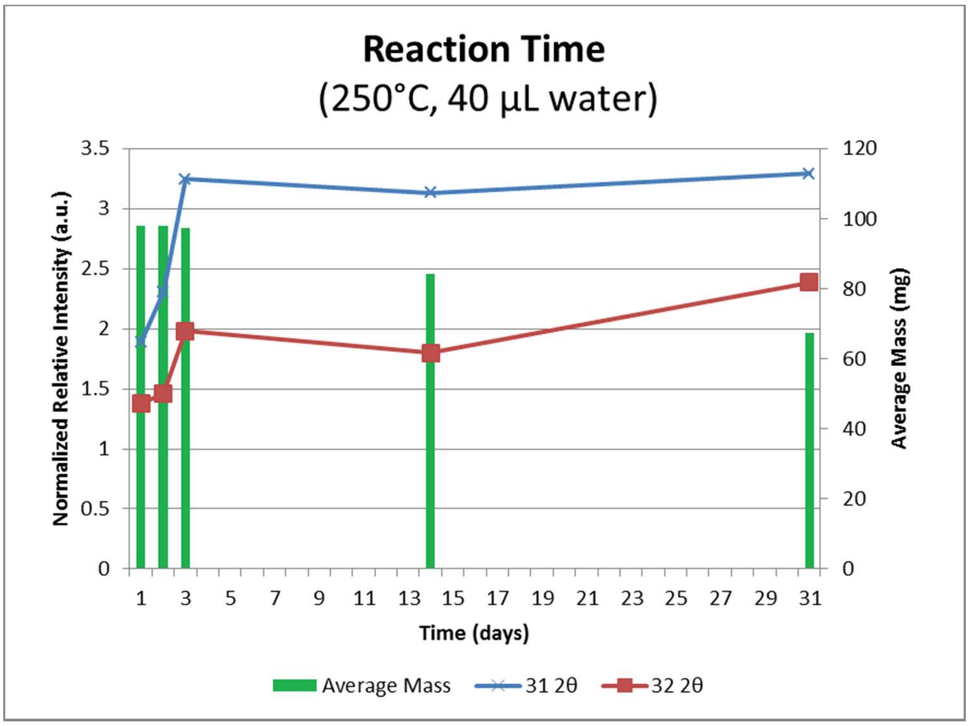

Supplementary Figure 10 The normalized relative crystallinity of **CAF-1** samples made with different reaction times. The blue line is a plot of the 211 series as outlined in the Supplementary Methods. The red line is a plot of the 202 series as outlined in the caption of Supplementary Figure 9. The green bars are the average mass of samples isolated from the various syntheses. The syntheses were carried out following the procedure for the synthesis of **CAF-1** from **PATnC** as describe in the Supplementary Methods section expect that 40 µL of water was used and the reaction time was varied.

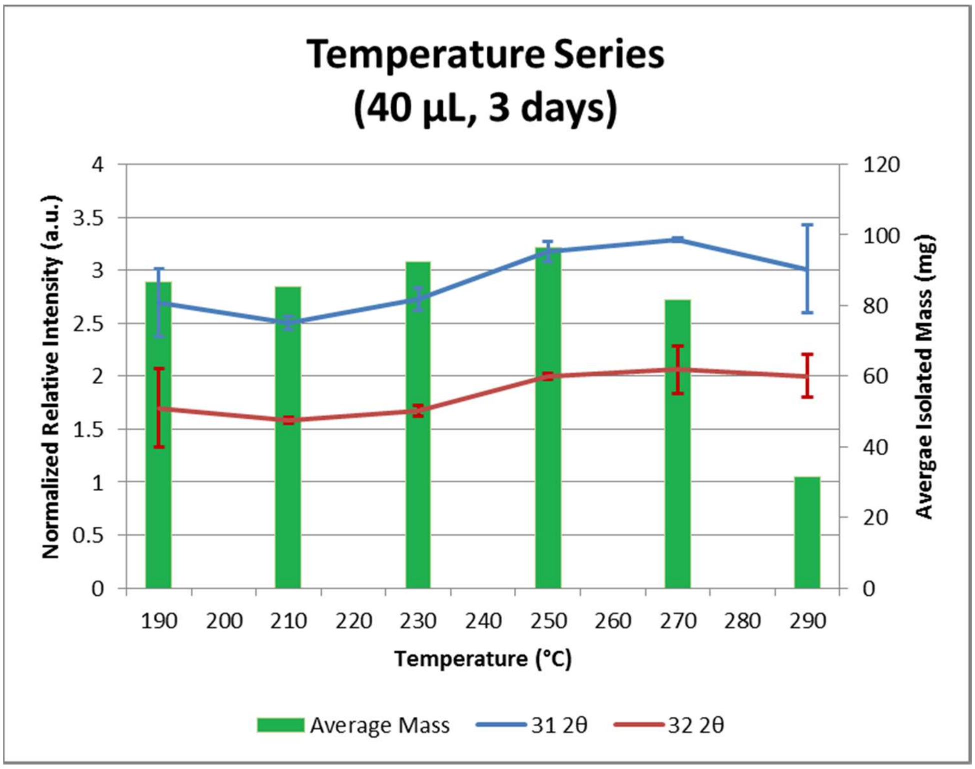

Supplementary Figure 11 The normalized relative crystallinity of **CAF-1** samples made at different reaction temperatures. The blue line is a plot of the 211 series as outlined in Supplementary Methods. The red line is a plot of the 202 series as outlined in Supplementary Methods. Error bars shown are 1 standard deviation for the mean average of relative crystallinities calculated from three repeats of each experiment. The green bars are the average mass of samples isolated from the various syntheses. The syntheses were carried out following the procedure for the synthesis of **CAF-1** from **PATnC** as describe in the Supplementary Methods section except that 40 µL of water was used and the reaction temperature was varied.

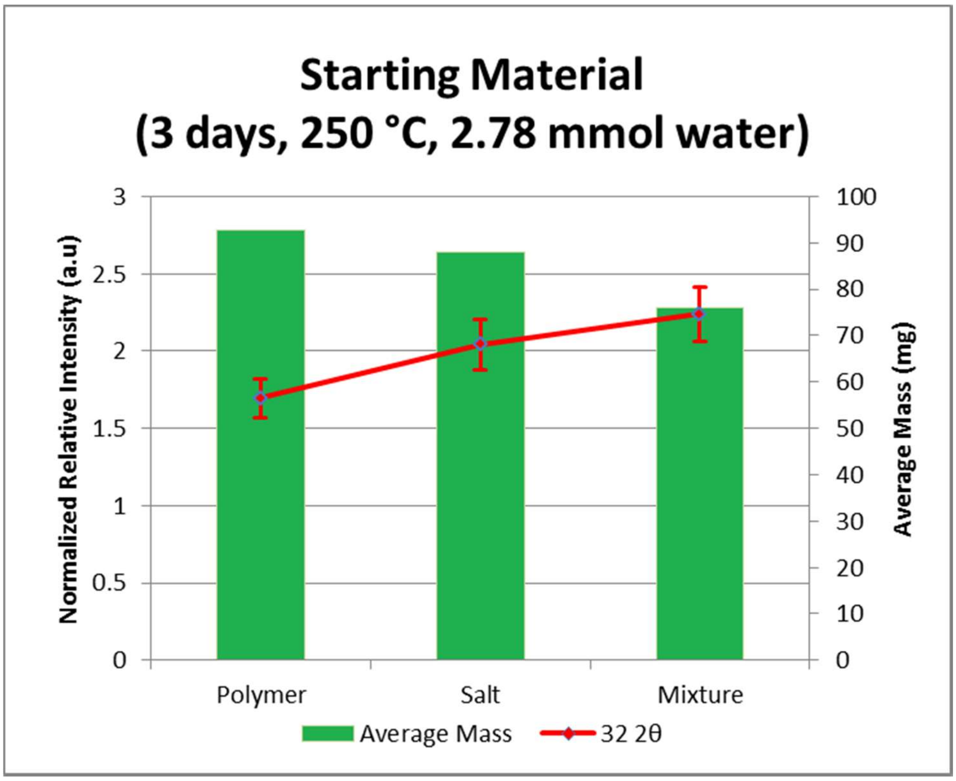

Supplementary Figure 12 The normalized relative crystallinity of **CAF-1** samples made from different starting materials. The red line is a plot of the 202 series as outlined in Supplementary Methods. Error bars shown are 1 standard deviation for the mean average of relative crystallinities calculated from three repeats of each experiment. The green bars are the average mass of samples isolated from the various syntheses. The syntheses were carried out following the procedures for the synthesis of **CAF-1** from **PATnC**, **1** (salt) and a physical mixture of starting materials as describe in the Supplementary Methods section with a total amount of free water (added, water of crystallization from the salt and water of reaction from salt and mixture) in all cases being 2.78 mmol

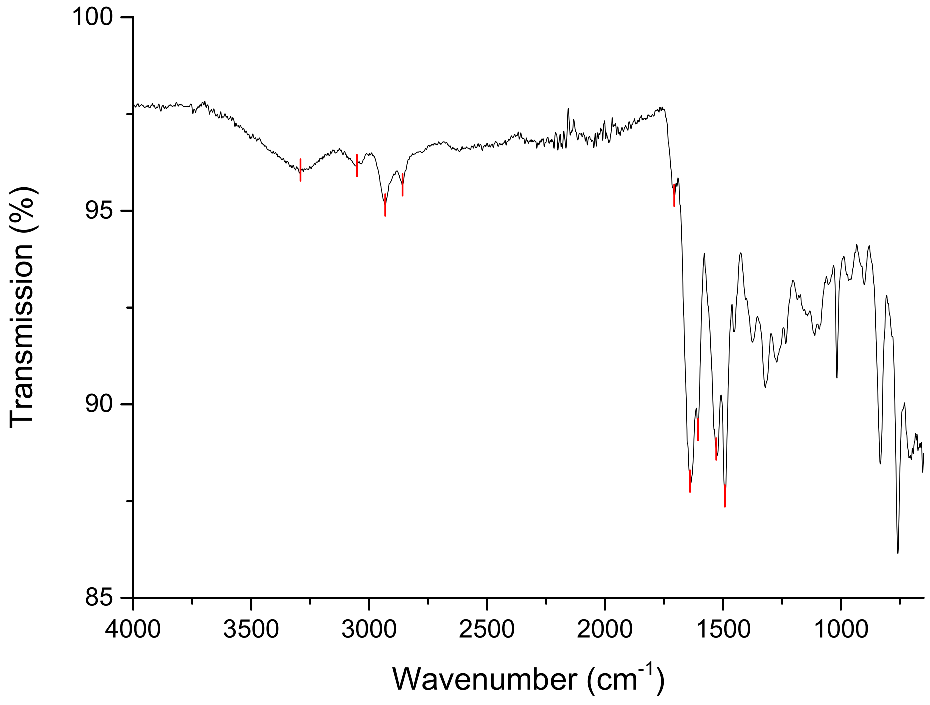

Supplementary Figure 13 FTIR spectrum of **PATCnC**

Supplementary Table 5 – FTIR peak table of **PATCnC**

Peak Centre (cm^{-1})	Transmission (%)	Assignment
**3291**	96.1	N-H stretch
**3051**	96.2	C-H stretch
**2932**	95.1	C-H stretch
**2858**	95.7	C-H stretch
**1707**	95.4	C=O (COOH) stretch
**1640**	88.0	C=O (amide) stretch
**1606**	89.3	
**1529**	88.8	N-H bend
**1492**	87.6	

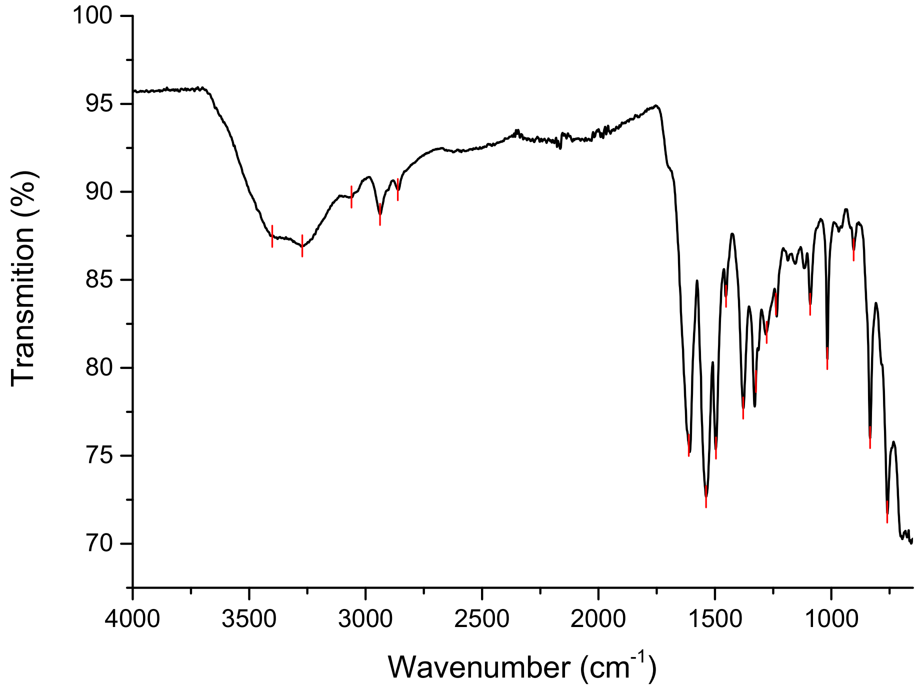

Supplementary Figure 14 FTIR spectrum of **CAF-2**

Supplementary Table 6 FTIR peak table of **CAF-2**

Peak Centre (cm^{-1})	Transmission (%)	Assignment
3401	87.5	N-H stretch
3272	86.9	N-H stretch
3060	89.7	C-H stretch
2938	88.7	C-H stretch
2862	90.1	C-H stretch
1612	75.6	C=O stretch
1538	72.7	C-N stretch
1496	75.4	
1452	84.1	
1379	77.7	
1324	79.2	
1278	82.0	
1238	83.6	
1091	83.6	
1017	80.5	
904	86.7	
384	76.0	
760	71.8	

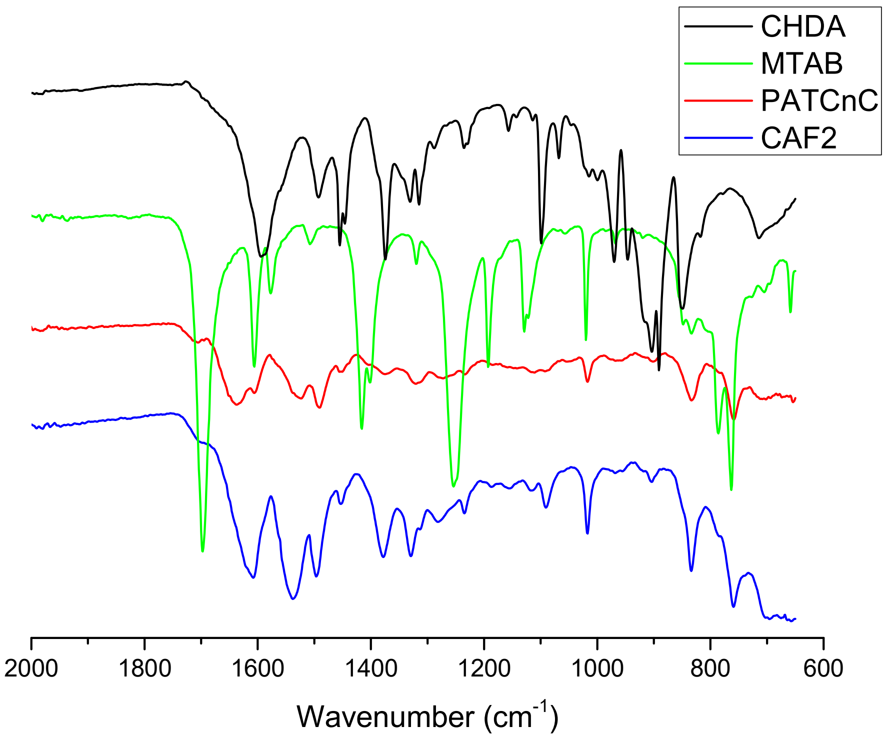

Supplementary Figure 15 Comparison of FTIR spectrums of C=O stretch region for **CAF-2** and precursors

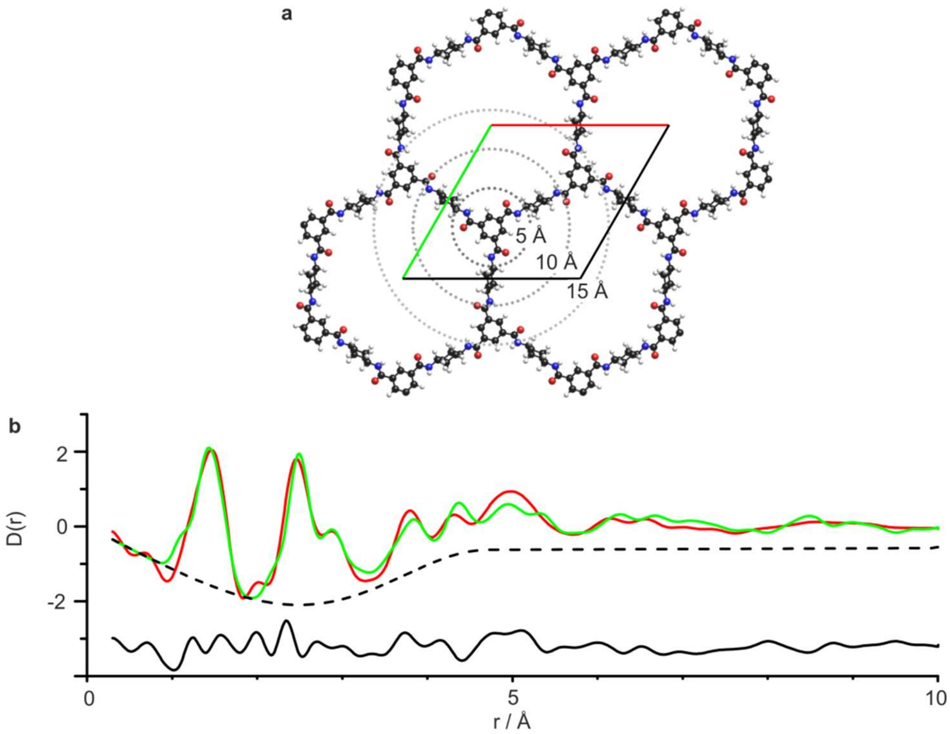

Supplementary Figure 16 (a) Structural model for **PATnC** consisting of a single hexagonal sheet that was used to fit the observed pair distribution function (PDF) data of **PATnC.** (b) PDF datasets for **PATnC**. Experimentally obtained data are shown in red, calculated in green and the difference in black. In order to account for the unphysical nature of a single layered model, a function equivalent to the contribution of uncorrelated atomic density was added to the data; this function is overlaid as the dashed line. The short coherence length of **PATnC** (12 Å, in a single hexagonal sheet the translation distance of one TMA to another is 13 Å) means that only contributions of the nearest monomers are observed (for example, from the centre of one TMA unit to the next via an intervening CHDA unit) with everything beyond this length-scale being structurally uncorrelated. Despite this, the fit shows a reasonable match, indicating that the short-range structure and stoichiometry of an amide network are consistent with the data.

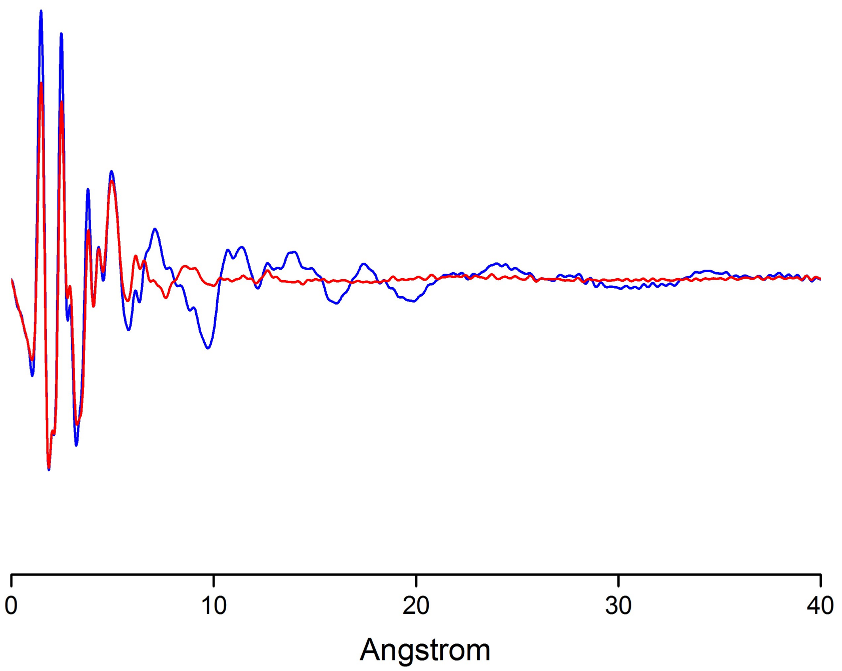

Supplementary Figure 17 Comparison between the PDFs of **PATnC** (red) and **CAF-1** (blue) show that in both phases at short-range (up to 5 Å) the structure is the same, this is to be expected as this length scale is dominated by the internal correlations of the different molecular units and their local connectivity. This confirms that the molecular connectivity of **PATnC** is not changed upon devitrification and it is only the long-range ordering which is significantly altered with the coherence length increasing from one TMA translation in **PATnC** to three translations in **CAF-1**.

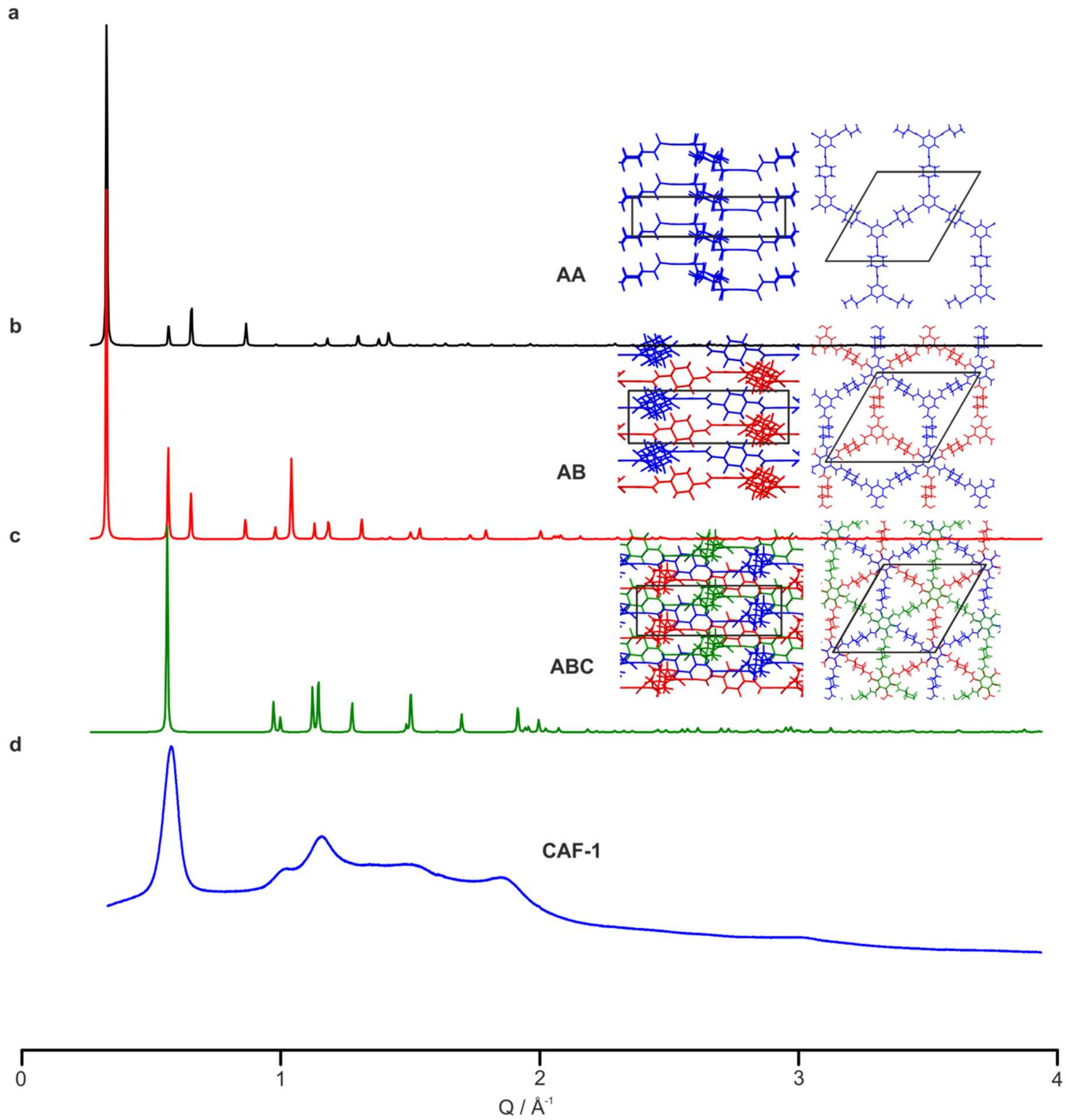

Supplementary Figure 18 DFT modelling of the potential different layer stackings of **CAF-1** and their simulated PXRD patterns. These models of **CAF-1** were produced with different stacking patterns of single hexagonal sheets, as shown above. (a) The AA stacked model has $P\overline{3}$ symmetry with a = 22.18 Å and c = 5.00 Å. TMA groups within the column are thus separated by 5.00 Å, which is too large to expect a significant π–π stacking contribution to interlayer interactions. (b) The model with AB stacking has $P6_3$ symmetry with a = b = 22.24 Å and c = 6.36 Å. (c) The ABC stacked model is the lowest in energy, and has $R3$ symmetry with a computed trigonal unit cell of a = b = 22.40 Å and c = 6.66 Å (a = b = c = 13.12 Å, α = β = γ = 117.20° in the rhombohedral setting). (d) Experimentally obtained PXRD pattern of **CAF-1**.

Supplementary Note 3 DFT modelling of **CAF-1**

The analysis of pore dimensions and size distribution performed using olex2[23] and a probe radius of 1.5 Å indicates that in both the *R*3 structure with identical TMA columns and in the lowest energy cell with mixed TMA columns there are two types of pores: large and small. The combined free volume of one small and one large pore is approximately 120 $Å^3$ in both cases, which is available to accommodate guest molecules. With the volume of a water molecule being close to 30 $Å^3$, this suggests the upper limit for water uptake to be (120 / 30) × 3 = 12 water molecules per unit cell containing 6 formula units in either structure. Experimental data indicate the presence of 7.2 to 9.9 water molecules in such a unit cell (Supplementary Table 4).

The preferential adsorption of water molecules via hydrogen bonding to the carbonyl oxygen of the amide bond was confirmed by DFT calculations using the rhombohedral unit cell containing two formula units (the primitive cell of the *R*3 structure) and a single water molecule. The water molecule with a random orientation was placed in the centre of the pore and then the structure of the flexible cell was optimised allowing the water molecule to adsorb at the nearest adsorption site. Three simulations were performed for both small and large pores and the adsorption energies were calculated with respect to gas phase as $E_{ads} = E_{MOF+water} – E_{MOF} – E_{water}$ (Supplementary Table 8). In all six calculations the guest water molecule formed a hydrogen bond with the carbonyl oxygen of the framework with the main difference being the orientation of the water molecule with respect to the framework. Interestingly, while the small pore of an empty structure was found to be too small to accommodate a water molecule, the structural optimisation with the water molecule present resulted in the widening of the small pore due to the rotation of the cyclohexane units and lead to the lowest energy configuration observed among the six simulations E_{ads} = -50.9 kJ/mol. Supplementary Figure 19 illustrates the lowest energy structures for water molecule placed into the large pore (a) and the small pore (b).

To take into account the flexibility of the framework and the mobility of guest molecules at finite temperature we performed molecular dynamics simulations using the tight binding DFT approach as detailed in the Supplementary Methods section. The total of nine water molecules were placed into the six pores present in the trigonal cells of either high symmetry *R*3 structure or the mixed TMA column structure (six formula units). In one series of calculations all water molecules were placed into the large pores, three in each pore. In the other series of calculations two water molecules were placed into each large pore and one into each small pore. The simulations were performed at 300 K and 200 K to assess whether entropic effects at higher temperature affect the energy ranking between the four systems studied. After the initial equilibration of 20 ps in each simulation, the total energy was averaged over a 50 ps production run. These MD simulations (Supplementary Tables 9 and 10) indicate that for the high symmetry structure it is advantageous to have water molecules located within the large pores: moving one of three water molecules into the small pore increases the energy per formula unit by 8.9±2.2 kJ/mol at 200K and 7.3±2.8 kJ/mol at 300K in the least favourable out of the four cases considered here. The small differences in energy between the other three structures indicate that they are all likely to be present in the crystal structure contributing to its disorder especially at room temperature. The analysis of the MD trajectories indicates that, when three water molecules are present in the large pore, the configurations in which each water molecule forms a hydrogen bond with a carbonyl oxygen are rare (Supplementary Figure 20, a), whereas the most common

configuration shows a single water molecule bound to the framework with two other water molecules bound to it (Supplementary Figure, b).

The possible disorder in the ABC-stacked **CAF-1** structure associated with different patterns of hydrogen bonds on neighbouring TMA columns was investigated using the same DFT method as above (Supplementary Tables 7-10 and Supplementary Figures 19-21) except the symmetry constraints were removed and all 270 atoms in the monoclinic unit cell were allowed to move independently. There are three distinct TMA columns in each unit cell as shown in Fig. 4 of the main text and in Supplementary Figure 22. The structure of two out of three TMA columns was kept unchanged (marked with the blue and red circles in Supplementary Figure 22) whereas the structure of the third TMA column (marked with the green circles) was manually altered before the energy optimisation was performed. The blue circular arrows indicate the helical direction of –NH groups acting as hydrogen bond donors (Figure 3). The blue cross indicates that all –NH groups in the given column point into the page, whereas a blue circle marks the TMA columns with –NH groups pointing out of the page. All four distinct orientations of amide bonds resulting in a triple helix of the third TMA column were considered with the lowest and the highest energy structures shown in the figure above. The energies of all unit cells measured relatively to the cell with three identical TMA columns are given in Supplementary Table 11. The change to the clockwise helical direction of the –NH groups in a single TMA stack is energetically unfavourable as the rotation of the amide bonds by 180° required to achieve this configuration results in the –NH proton being placed close to the proton on the cyclohexane (H-C-N-H adopts cis-like rather than trans like conformation). Interestingly, the smaller rotation of the amide bonds by 80˚ on one of the TMA columns in the cell such that –NH bonds switch from into the page to out of the page (shown in Supplementary Figure 22, c) resulted in 2.7 kJ/mol decrease in energy. The DFT data suggest that the neighbouring TMA columns in the experimental structure are likely to have a different pattern of hydrogen bonds due to the +40° or −40˚ tilt of the amide bonds with respect to the plane of the TMA cores while the columns have identical clockwise or anticlockwise structure. As Supplementary Figure 22 demonstrates, with more than one type of the TMA columns present in the structure, the preferable orientation of the cyclohexane-diamide linker is not unique. Thus a crystal structure with mixed TMA columns is expected to have multiple orientations of the cyclohexane ring contributing to the crystallographic disorder.

The comparison between the simulated and the experimental ^{13}C NMR spectra show that the lowest energy calculated structure with two types of TMA columns matches experimental data much better than the high symmetry calculated structure in which all TMA columns are identical. This further supports the idea that two types of TMA columns coexist within the structure of **CAF-1**.

Supplementary Table 7 DFT modelling of water in the ABC stacked **CAF-1** model

	Large pore			Small pore		
	Window Å	Cavity Å	**Volume Å^3**	Window Å	Cavity Å	**Volume Å^3**
High symmetry	3.3	3.6	**118**	2.6	2.9	**-**
Mixed structure	2.9	3.5	**85**	2.5	3.4	**36**

Supplementary Table 8 DFT calculated adsorption energies of water in **CAF-1**

	calculation 1	calculation 2	calculation 3
Large pore	-48.6 kJ/mol	-46.6 kJ/mol	-36.5 kJ/mol
Small pore	-50.9 kJ/mol	-48.5 kJ/mol	-45.0 kJ/mol

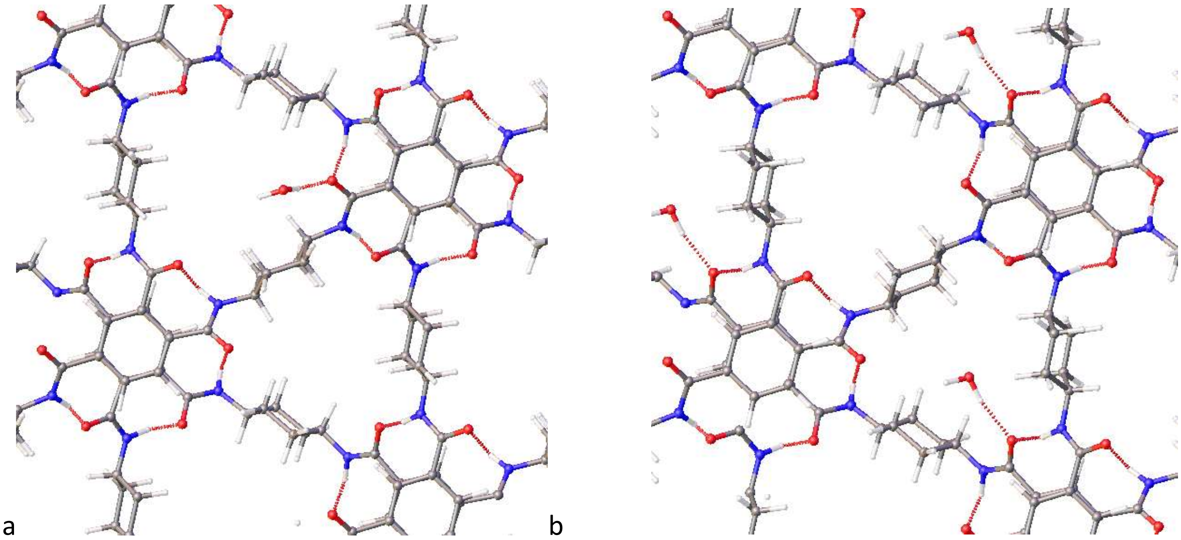

Supplementary Figure 19 Lowest energy configurations of water adsorbed in large (a) and small (b) pore of **CAF-1** from DFT calculations

Supplementary Table 9 Results of Molecular Dynamics calculations of water in **CAF-1** at 200 K

**T=200K**	3+0 water molecules	2+1 water molecules
High symmetry structure	Set to 0 kJ/mol	8.9 kJ/mol
Mixed structure	-2.6 kJ/mol	2.1 kJ/mol

The typical error corresponding to 95% confidence interval is 1.1 kJ/mol, 3+0 indicates 3 water molecules placed in each large pore and 0 in each small pore, 2.1 indicates 2 water molecules placed in each large pore and 1 in each small pore

Supplementary Table 10- Results of Molecular Dynamics calculations of water in CAF-1 at 300 K

**T=300K**	3+0 water molecules	2+1 water molecules
High symmetry structure	Set to 0 kJ/mol	7.3 kJ/mol
Mixed structure	0.9 kJ/mol	2.9 kJ/mol

The typical error corresponding to 95% confidence interval is 1.4 kJ/mol, 3+0 indicates 3 water molecules placed in each large pore and 0 in each small pore, 2.1 indicates 2 water molecules placed in each large pore and 1 in each small pore

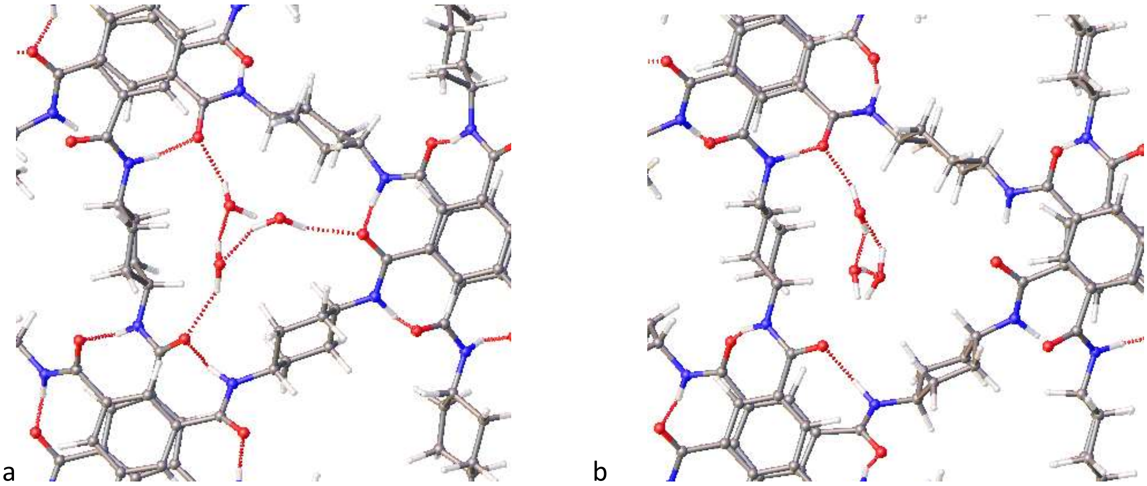

Supplementary Figure 20- Positions of water in the large pore of **CAF-1** based on MD calculations The MD simulations indicate that at room temperature the water molecules are expected to segregate in the large pore while the empty pore remains less occupied.

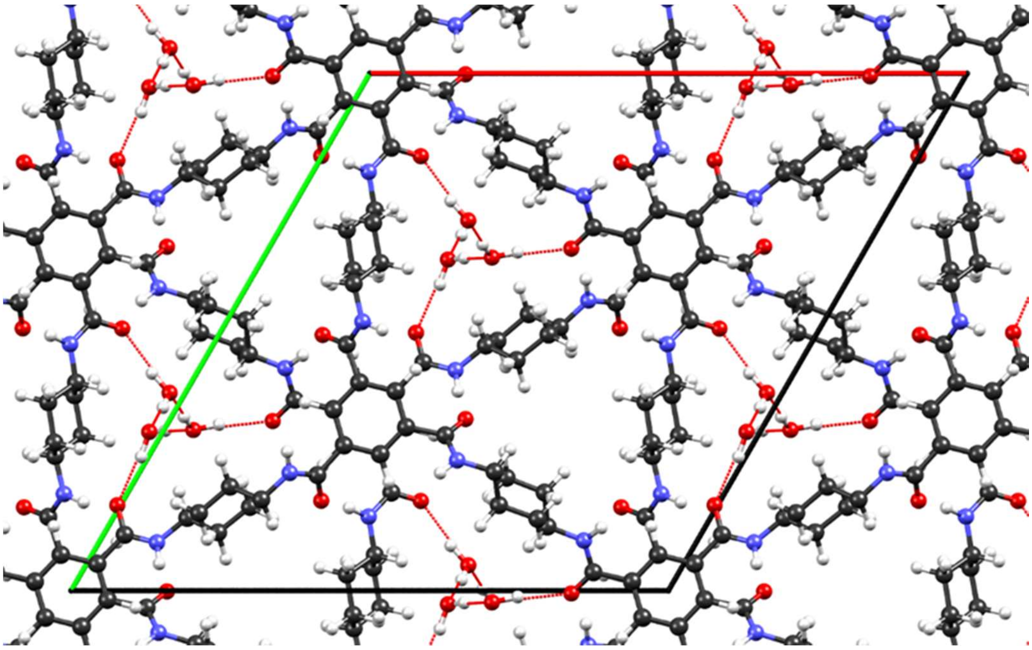

Supplementary Figure 21 Relaxed DFT Structural Model of **CAF-1** with *R*3 symmetry and three water molecules within each large pore was constructed and relaxed with DFT as a starting point for the analysis of X-ray diffraction and PDF data. The resulting model has the same H-bonding patterns in the TMA columns as the original empty structural model, and each water molecule donating H-bonds to a carbonyl oxygen and a neighbouring water molecule.

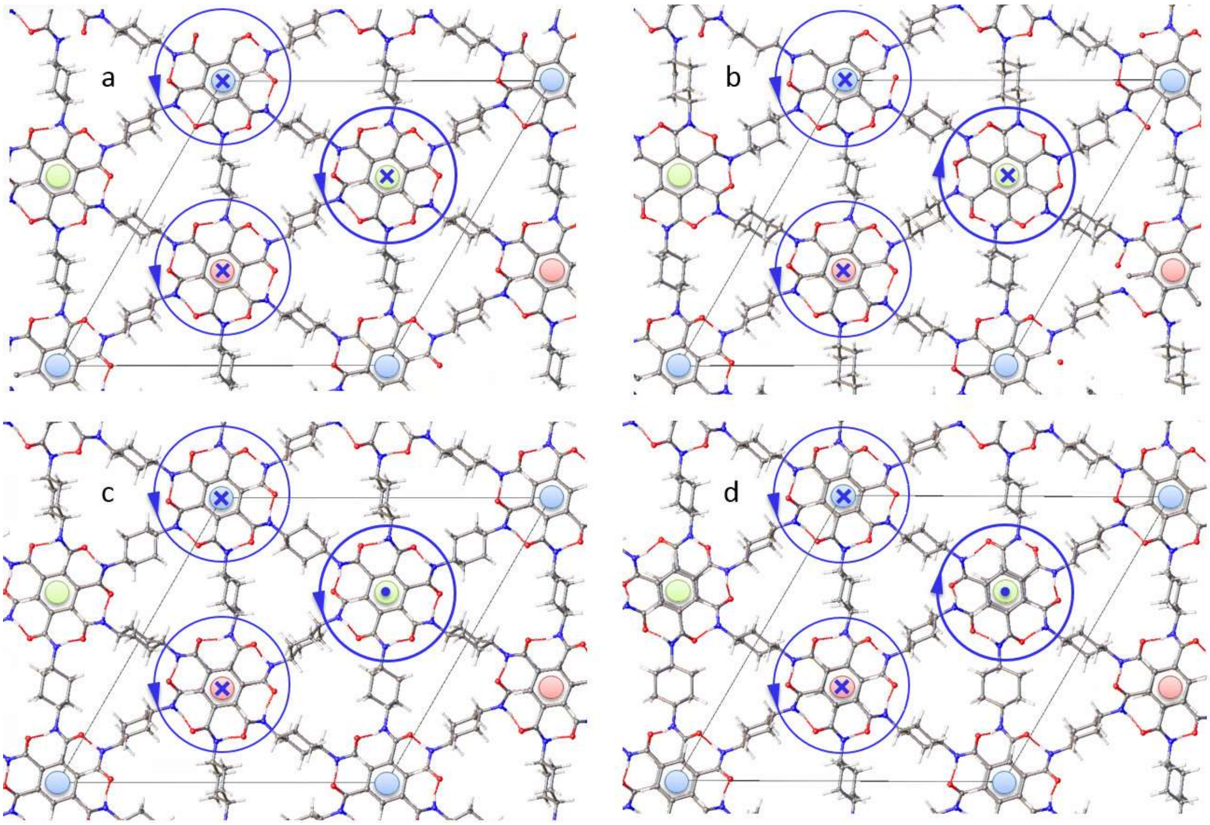

Supplementary Figure 22 DFT modelling of the potential different TMA stacks in ABC stacked **CAF-1** model. There are three distinct TMA columns in each unit cell and the two out of three TMA columns was kept unchanged (marked with the blue and red circles in) whereas the structure of the third TMA column (marked with the green circles) was manually altered before the energy optimisation was performed. The blue circular arrows indicate the helical direction of –NH groups acting as hydrogen bond donors. The blue cross indicates that all –NH groups in the given column point into the page, whereas a blue circle marks the TMA columns with –NH groups pointing out of the page.

Supplementary Table 11- Calculated Energies of Different TMA stack configurations

Energy difference per formula unit	-NH groups point down	-NH groups point up
Anticlockwise orientation of NH groups	Set to 0 kJ/mol	-2.7 kJ/mol
Clockwise orientation of NH groups	+4.8 kJ/mol	+14.7 kJ/mol

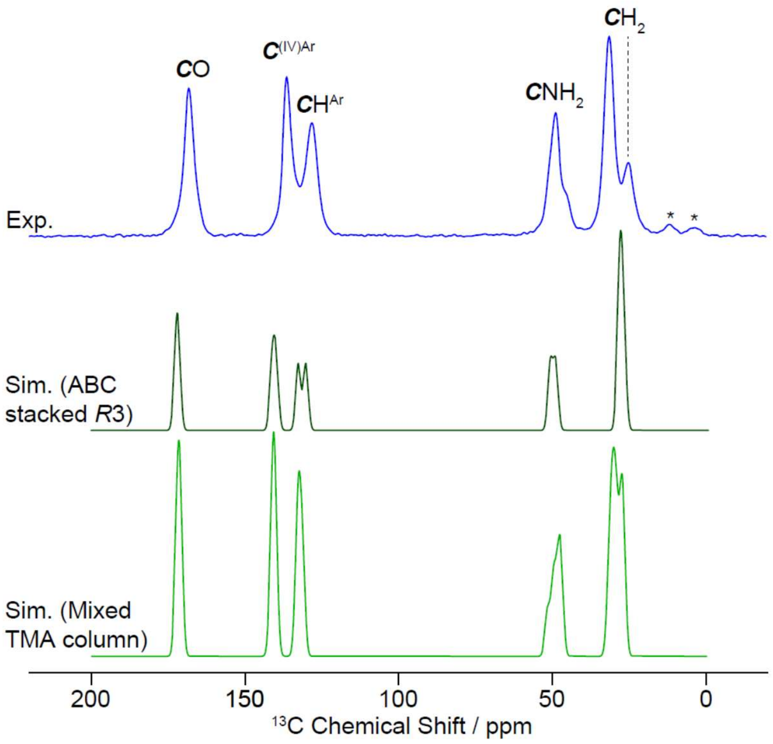

Supplementary Figure 23 Comparison of computed ^{13}C NMR parameters with experimental data. ^{13}C NMR parameters were calculated for the ABC stacked *R*3 structure described in Supplementary Figure 18 and Figures 4 and 5 with the corresponding simulated ^{13}C spectrum shown in dark green above. The same was done for the mixed TMA column structure in which the direction of –NH bonds in one TMA column is switched (light green). Comparison with experimental NMR data (blue), shows good agreement for both structures. However the structure with mixed TMA columns (Supplementary Figure 22, b) gives slightly better agreement, with the splitting of the CH_2 peak at ~30 ppm, a broader *C*HNH peak at ~50 ppm and a single CH^{Ar} peak at ~130 ppm being reproduced.

Supplementary Note 4 Structural solution and degree of interpenetration of **CAF-2**

The starting point for structural analysis was the consideration of potential 3D network topologies accessible to the system. There are two basic building units in the **CAF-2** structure which are present in a 1:2 molar ratio: a tetrahedral CTAB-derived unit, where the angles between the four branches are approximately 109° and the distance between the tertiary carbon atom and the four carbonyl carbon atoms at the extremities of each branch is approximately 6 Å; and a near-linear CHDA-derived unit, which is a cyclohexyl ring with amino-derived groups at the 1,4 carbons in *trans* configuration, where the intra-molecular N-N distance is also approximately 6 Å. The connection of tetrahedral base units by linear linkers in three dimensions is expected to produce a diamond net. A simple diamond net constructed from CTAB and CHDA building units would produce a very low density structure with approximately 18 Å between the central atoms of adjacent connected units: higher (more physical) densities can be accessed by forming interpenetrated diamond network structures, as observed for the imine based COF-300[24]. Attempts to index the PXRD pattern with the expected cell dimensions of interpenetrated diamondoid nets[24], led to the construction of structural models based upon 5-, 6-, 7- and 8-fold interpenetrated diamond nets, which were each relaxed by DFT. The space groups of the DFT relaxed structures were assigned using FINDSYM,[14] as $I4_1/a$, $P4_2/n$, $I4_1/a$ and $P4/n$ respectively. Simulated PXRD patterns were produced from these structures and compared qualitatively with the experimental PXRD pattern of **CAF-2**. The 7-fold interpenetrated diamond net (a tetragonal unit cell in space group $I4_1/a$) was found to best match the main features of the experimental diffraction pattern, and this was further improved by repeating the DFT relaxation of this structure with 12 water molecules per formula unit (as indicated by CHN microanalysis of **CAF-2**), which was found to suppress the dominant intensity of the (200) peak. This candidate was used as the basis for crystallographic analysis. The simulated PXRD patterns (λ = 0.826189 Å) are shown below.

A Pawley fit to the experimental PXRD data, with refinement of the lattice parameters of the tetragonal DFT-relaxed 7-fold interpenetrated diamond structure, produced values of $a = b = 26.118$ Å, $c = 7.581$ Å. This was found to fit the main features of the pattern but produced a mis-fit at 2θ = 21.5°. The powder pattern was therefore re-indexed using the auto-indexing routine in TOPAS 5, which resulted in the predominance of *I*-centred orthorhombic cells amongst the most highly ranked candidates. A new Pawley fit using an orthorhombic cell in *Imma* (the highest symmetry candidate) produced an excellent fit to the observed data with refined lattice parameters of $a = 26.531$, $b = 26.120$ and $c = 7.588$ Å. These dimensions correspond to a small orthorhombic distortion of the unit cell. Consequently, prior to structure solution, the tetragonal DFT cell in $I4_1/a$ was transformed into its sub-group $I2/a$ using the ISOTROPY suite[25] and set to be metrically orthorhombic (α = β = γ = 90°). An initial Rietveld refinement used the DFT model in $I2/a$, with all atomic positions fixed, lattice parameters fixed to values obtained by Pawley refinement ($a = 26.531$, $b = 26.120$, $c = 7.588$ Å, α=β=γ= 90°), and a fixed background and peak profile function. This produced a poor fit to the observed intensities (R_{wp} = 9.0, $\chi^2 = 3236$, Supplementary Figure 26).

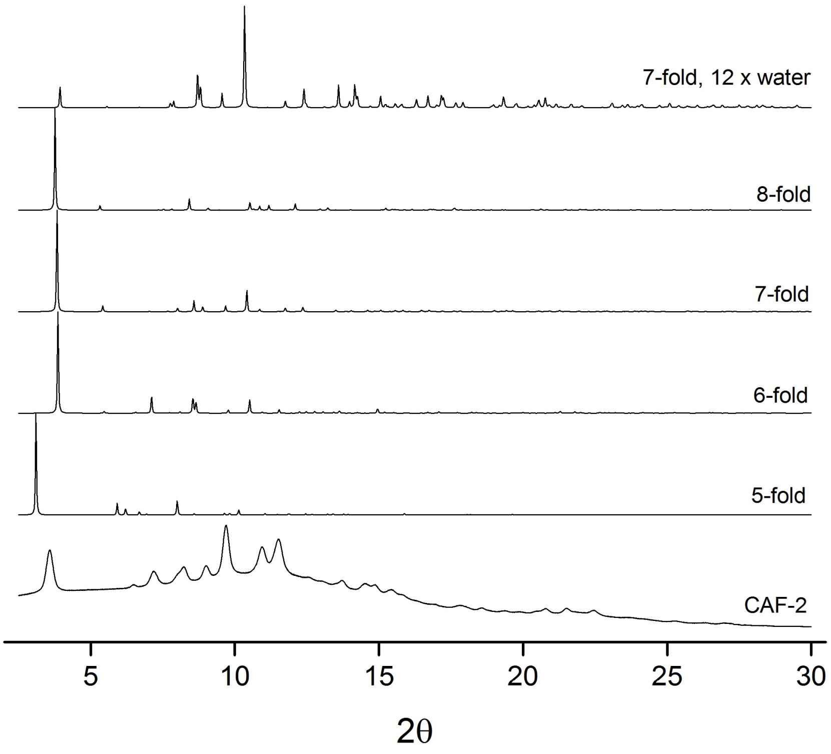

Supplementary Figure 24 Simulated PXRD patterns were produced from the structural models based upon 5-, 6-, 7- and 8-fold interpenetrated diamond nets structures and compared qualitatively with the experimental PXRD pattern of **CAF-2**.

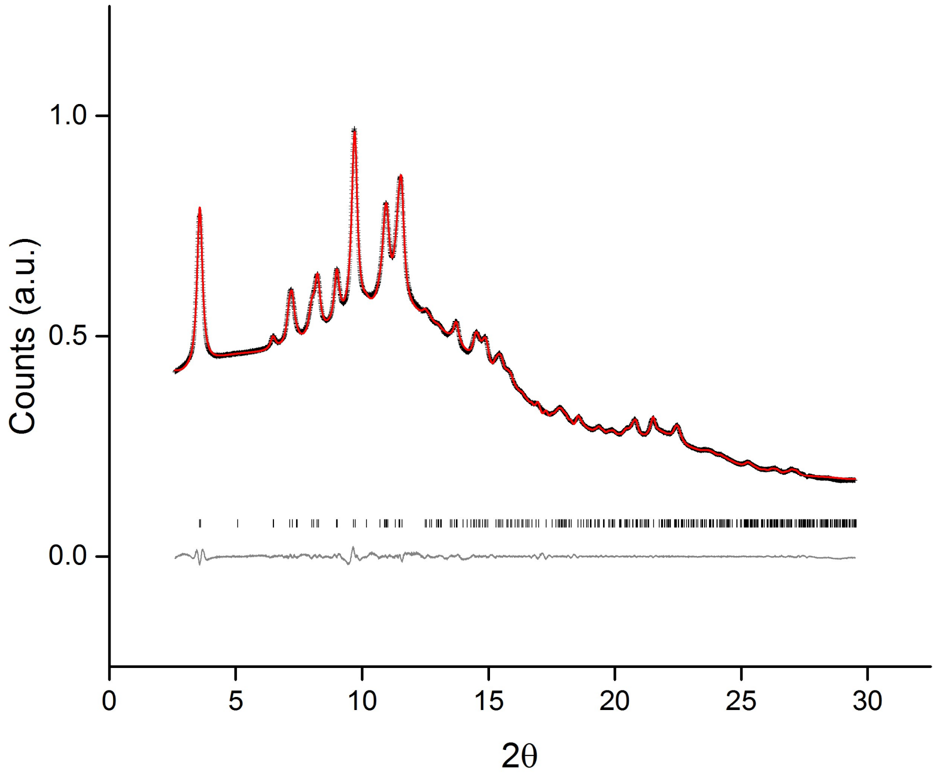

Supplementary Figure 25 – Initial Pawley fit of CAF-2 A new Pawley fit using an orthorhombic cell in *Imma* (the highest symmetry candidate) produced an excellent fit to the observed data with refined lattice parameters of *a* = 26.531, *b* = 26.120 and *c* = 7.588 Å.

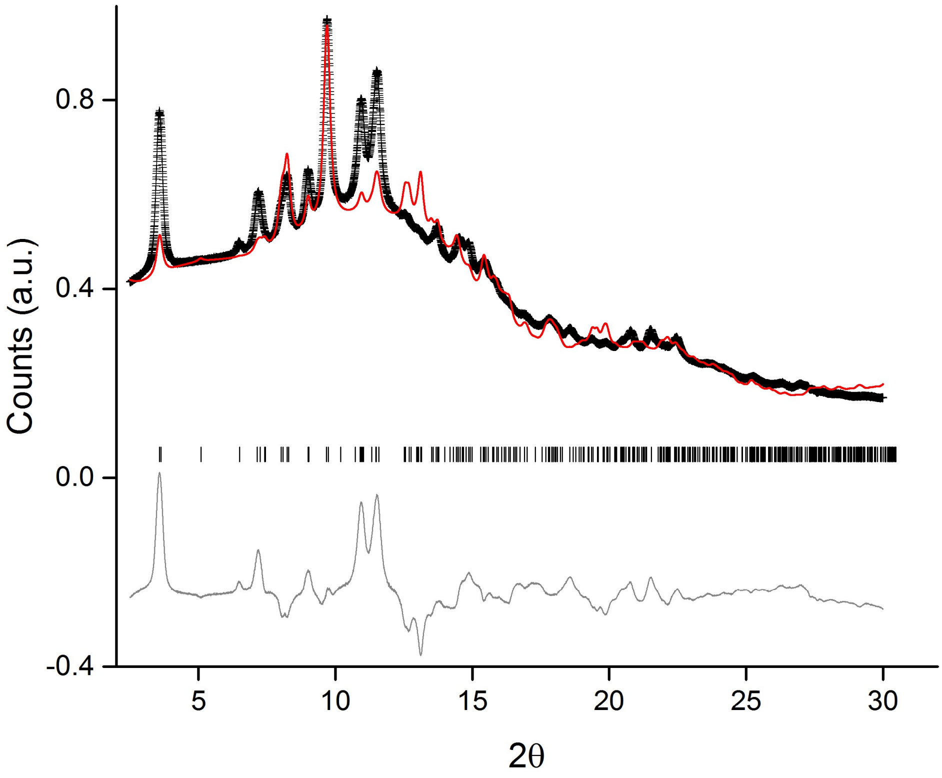

Supplementary Figure 26 Initial Rietveld Refinement of **CAF-2**

Supplementary Note 5 Simulated Annealing of the structural model of **CAF-2**

Initial attempts at simulated annealing of the H_2O coordinates within a fixed framework was found to substantially improve the fit, but tended to produce a number of water sites in non-physical positions within the framework, implying a need to refine the framework itself. To enable a full Rietveld refinement of the structure a rigid body model of the **CAF-2** framework was constructed. Three types of rigid body were defined using a z-matrix formulation: phenyl fragments (C_7H_4) with a refinable C-C distance; amide (CHNO) fragments with refinable C-N, C-O distances and N-C-O angle; and cyclohexyl (C_6H_{10}) fragments with refinable C-C distance and a refinable parameter controlling the puckering of the ring. The individual rigid bodies were linked together either by hard constraints (the amide-phenyl linkage) or by soft bond distance and angle restraints (the amide-cyclohexyl linkage). Hydrogen atoms were not included in the model. Ten independent oxide ions, used to model scattering by guest water molecules, were included with initial atomic positions corresponding to those in the DFT relaxed model and occupancies set to 0.5 (thus allowing a degree of disorder in positions of the guest species).

The rigid body model was then refined in two stages. First, it was fitted to the synchrotron PXRD data by simulated annealing of the oxide (water) coordinates, with simultaneous refinement of the rigid body orientations and bond distances/angles, whilst the lattice parameters, instrument parameters, isotropic thermal displacement parameters, peak profile, background function and scale factor were all fixed (either to values derived from the earlier Pawley fit, or to nominal values as appropriate). This produced an approximate fit to the data with oxides located in physically reasonable positions. Second, this model was used as the starting point for a Rietveld refinement: the atomic coordinates of the oxide ions were refined along with the rigid body rotations and bond distances/angles, and a single refined isotropic thermal displacement parameter was applied to all framework atoms. The scale factor was refined, with the background modelled by a refined 24-term Chebyschev polynomial, and peak shape by a pseudo-Voigt function. In the final round of refinement, one pair of oxide ions (half occupied) whose positions had refined to be equal within error were merged into a single fully-occupied site. This produced a very good fit to the major features of the diffraction pattern, with agreement factors R_{wp} = 1.44 and χ^2 = 82.3, which is plotted in the main manuscript in Figure 4.

In the refined structure (see supporting crystallographic information file, CAF2.cif), the tertiary carbon atoms of CTAB units from adjacent interpenetrated networks are separated by 7.58(6) Å, which is considerably shorter than the ~18 Å expected from a simple non-interpenetrated diamond net. The refined bond distances and angles were found to be within error of physically sensible values: the local geometry about the tertiary carbon of the CTAB units contains four C-C-C angles within 3 estimated standard deviations of ideal tetrahedral geometry, whilst the remaining two angles of 103.4(9)° and 112.9(5)° show that the unit is subjected to a small distortion. The cyclohexyl rings of the two crystallographically distinct CHDA units retain the chair conformation which implies that the orientations of the cyclohexyl groups are ordered (note that the cyclohexyl rigid body had the freedom to refine to a flat phenyl-type conformation, which would represent full orientational disorder of the cyclohexyl rings). Despite having the same cyclohexyl ring conformations, the two crystallographically distinct CHDA units still produce two different local conformations within the network, with amide groups adopting either axial/axial or equatorial/equatorial conformations.

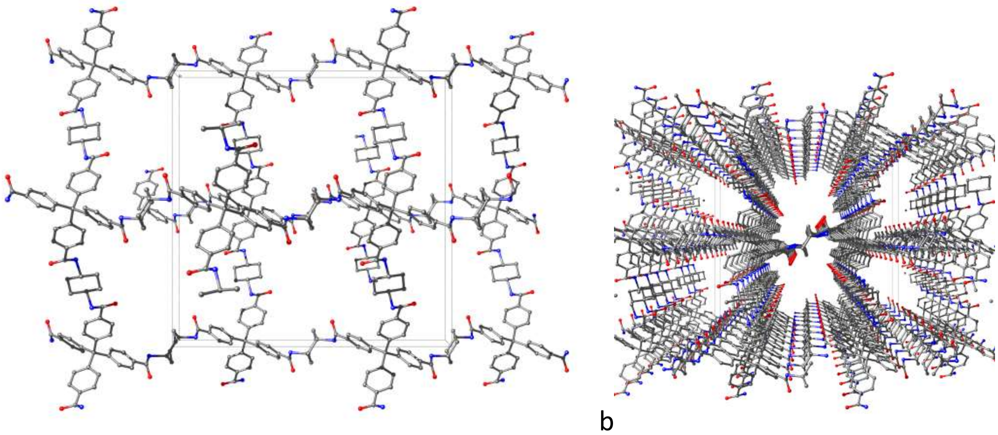

a b

Supplementary Figure 27 Views of CAF-2 structural model down the b-axis, (a) showing a single net, (b) showing 7-fold interpenetrated nets. A single diamondoid net has three-dimensional porosity, however the stacking of interpenetrated nets along the *b* crystallographic axis, reduces the porosity to one-dimensional channels along this stacking direction.

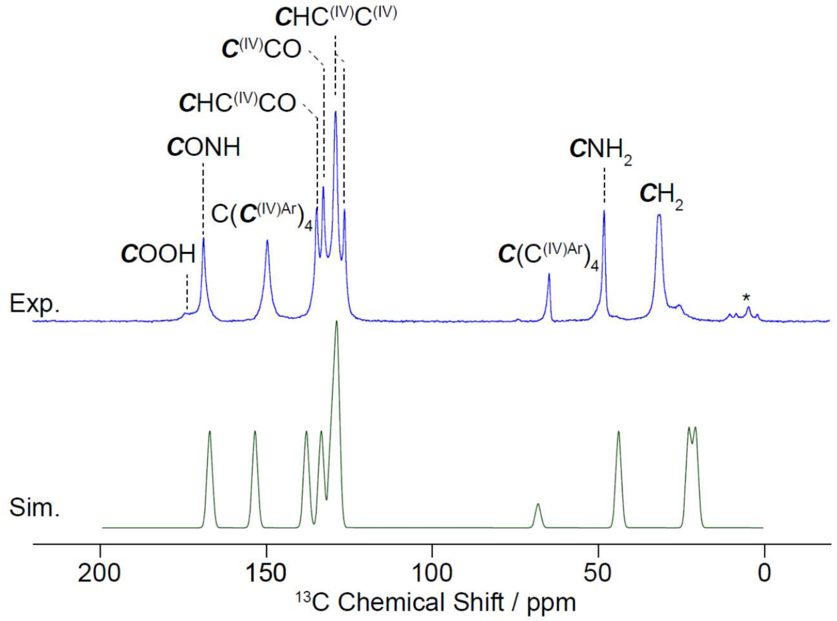

Supplementary Figure 28 –The simulated ^{13}C NMR spectrum of **CAF-2** (green) is in reasonable agreement with the experimental spectrum (blue), showing that the proposed 7-fold interpenetrated diamondoid net structure of **CAF-2** is consistent with all the available experimental data.

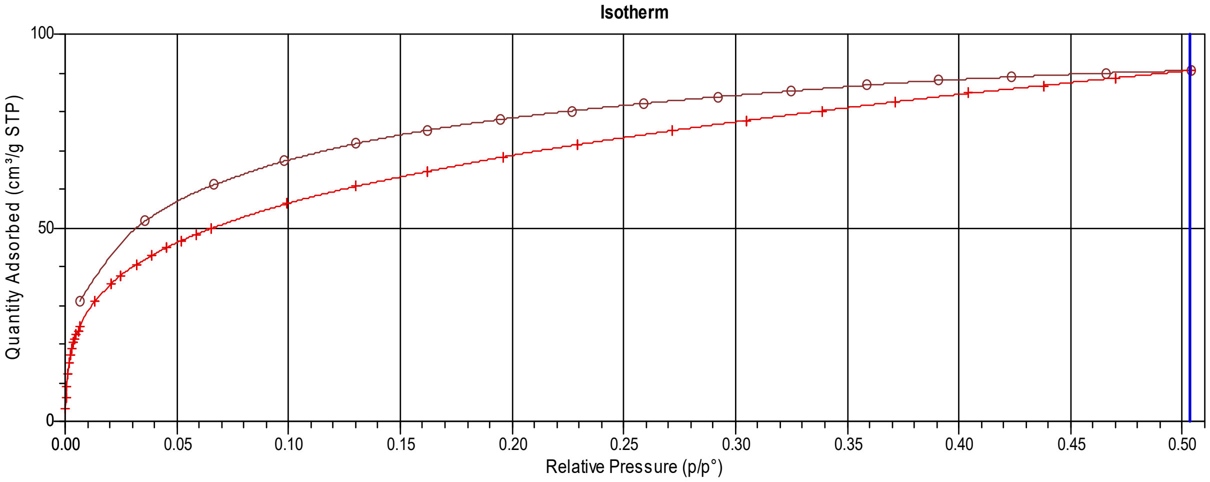

Supplementary Figure 29 CO_2 adsorption – desorption isotherm of **CAF-1** at 195 K

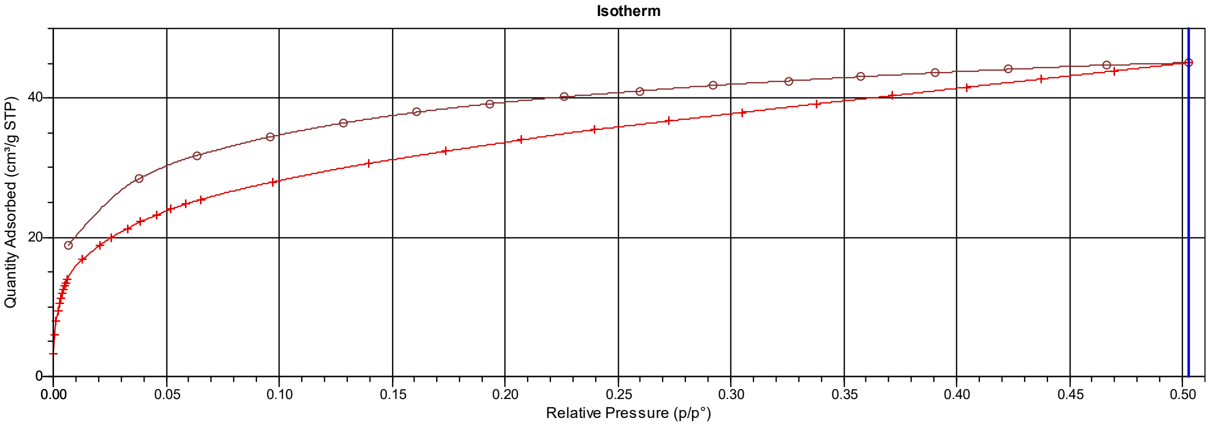

Supplementary Figure 30 CO_2 adsorption – desorption isotherm of **PATnC** at 195 K

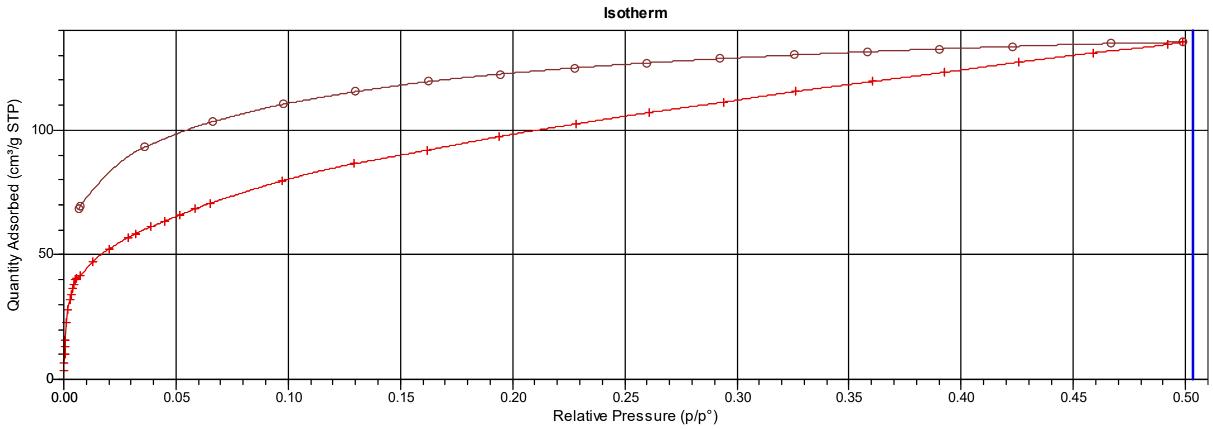

Supplementary Figure 31 CO_2 adsorption – desorption isotherm of **CAF-2** at 195 K

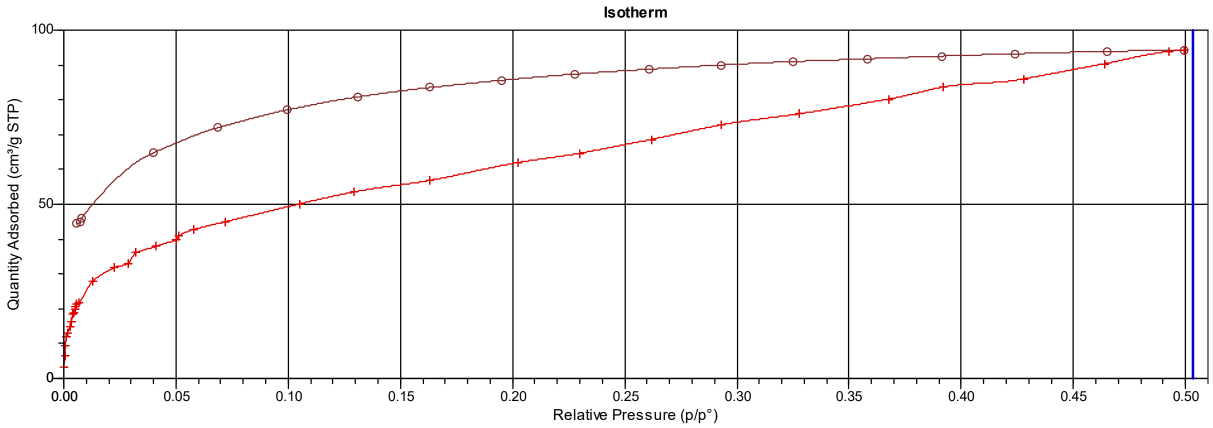

Supplementary Figure 32 CO_2 adsorption – desorption isotherm of **PATCnC** at 195 K

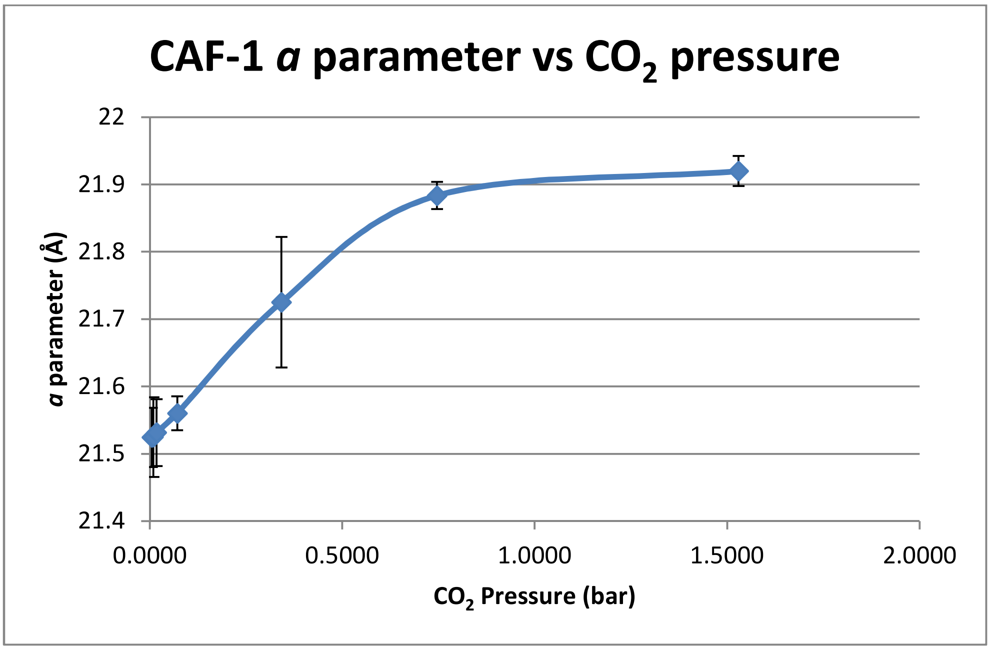

Supplementary Figure 33 Unit cell parameter *a* of **CAF-1** upon CO_2 adsorption with a plateau occurring at around 1 bar. Error bars shown on graphs are 1 SD.

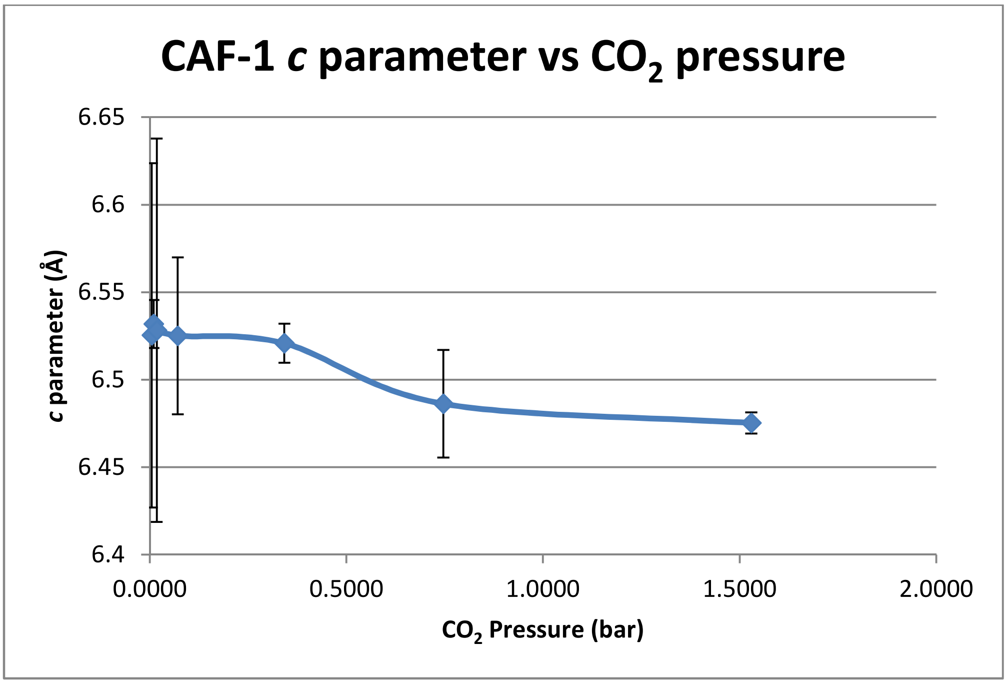

Supplementary Figure 34 Unit cell parameter *a* of **CAF-1** upon CO_2 adsorption with a plateau occurring at around 1 bar. Error bars shown on graphs are 1 SD.

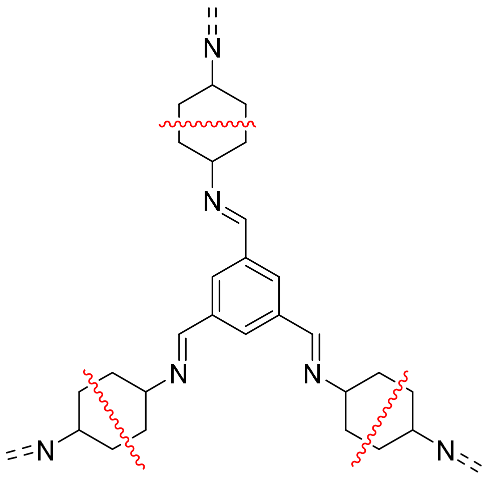

PITnC

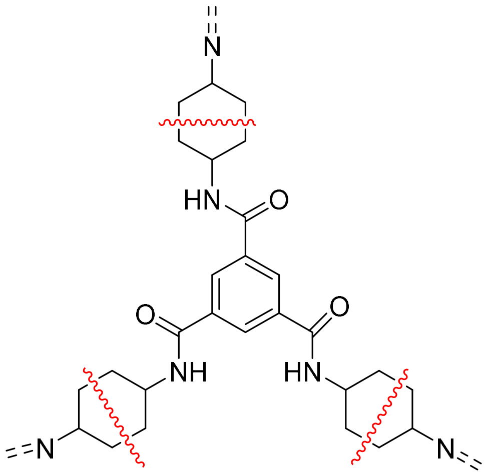

PATnC

Supplementary Figure 35 Molecular structures of PITnC and PATnC. In order to compare the hydrolytic stability properties of **CAF-1** and **PATnC** to that of a suitable like- for-like imine analogue Polyimine(Benzene-1,3,5-tricarboxaldehyde-net-trans-1,4-cyclohexyldiamine) (**PITnC**) was synthesised (Supplementary Methods).

Supplementary Note 6 100 °C Stability testing

In order to compare the hydrolytic stability of **CAF-1** and **CAF-2** to the state-of-the-art most stable imine COFs literature stability testing conditions were used[27]. Samples of **CAF-1, CAF-2**, **PATnC**, **PATCnC** and **PITnC** were suspended in either water, 1M $HCl_{(aq)}$ or 1M $NaOH_{(aq)}$ (~4 mL). These suspensions were tightly sealed in glass vials with Teflon caps and placed in an oven heating to 100 °C at 10 $°Cmin^{-1}$ and held at that temperature for 24 hours followed by cooling back to room temperature at the same rate. The suspensions were filtered onto pre-weighed filter tubes and then washed with water until the pH of the filtrate was neutral with a minimum volume of 100 mL. The samples were then further washed with DMF, MeOH and Acetone (100 mL each) in turn. The samples were dried overnight under vacuum in the pre-weighed filter tubes and then the masses recorded subtracting the mass of the filter tube to yield the residual masses of the remaining materials (Supplementary Table 12 and Figure 5). All samples were then analysed CHN microanalysis (Supplementary Figure 38). In addition **CAF-1** and **CAF-2** samples were also analysed for BET surface area (Figure 5) and PXRD (Supplementary Figures 36 and 37) to confirm that crystallinity was retained, this level of analysis goes beyond that used in the literature for these conditions.

In the case of NaOH suspensions it was found that the above washing procedure did not remove all NaOH from the networks of **CAF-1**, **PATnC** and **PITnC**. As such NaOH suspensions of these materials were filtered on paper after removing from the oven and the filtered paper folded into a parcel and then this was Soxhlet extracted with water for 3 days, with the water being replaced each day and the pH monitored. Once the pH remained neutral the Soxhlet extraction was stopped and the samples dried in a vacuum overnight. This was then followed by the same washing procedure as for the other samples.

Upon filtration of **CAF-2** samples it was found that the particle size after stability testing was too small for the filters used (0.2 micron) and that a large amount of the powder was lost. As such the work up of **CAF-2** samples was modified. After the test was completed the suspensions of **CAF-2** were transferred to pre-weighed centrifuge tubes and diluted up to 35 mL with the addition of a drop of either 1M HCl or 1M NaOH to the basic and acidic suspensions respectively. These were then centrifuged at 14K rpm for 20 minutes. Next the solution was decanted carefully and its pH checked. This process was repeated until the pH of all solutions was neutral (3 repeats). Next the powders were suspended in DMF, MeOH and Acetone in turn and the centrifuging/decanting steps repeated. Finally the centrifuge tubes were dried *in vacuo* and weighed. Subtraction of the weight of the pre-weighed tubes gave the residual mass of **CAF-2**. It is worth noting that despite our best efforts there were still suspended materials visible in the decanted solvent resulting in mechanical losses for **CAF-2** not observed for the other samples. Therefore the mass losses reported here for **CAF-2** should be considered as an upper limit. Because of this, entries for **CAF-2** in Supplementary Table 12 and in Figure 5 are formatted differently from the other data in order to highlight the different work-up methods used.

Supplementary Table 12 Residual masses of **CAF-1**, **PATnC**, **PITnC**, **CAF-2** and **PATCnC** after 100 °C stability testing

Sample	Conditions	Initial Mass (mg)	Residual Mass (mg)	Residual Mass (%)
**CAF-1**	Water	30.5	30	98
**CAF-1**	1M HCl	30.0	28.4	95
**CAF-1**	1M NaOH	30.6	28.8	94
**PATnC**	Water	20.6	20.6	100
**PATnC**	1M HCl	20.2	20.1	>99
**PATnC**	1M NaOH	69.8	53.7	77
**PITnC**	Water	19.8	18.3	92
**PITnC**	1M HCl	20.1	0	0
**PITnC**	1M NaOH	60.4	26.3	44
**CAF-2**	Water	30.7	25.1	82
**CAF-2**	1M HCl	30.3	21.5	71
**CAF-2**	1M NaOH	43.3	27.7	64
**PATCnC**	Water	30.3	30.3	100
**PATCnC**	1M HCl	30.5	30.0	98
**PATCnC**	1M NaOH	30.3	29.6	98

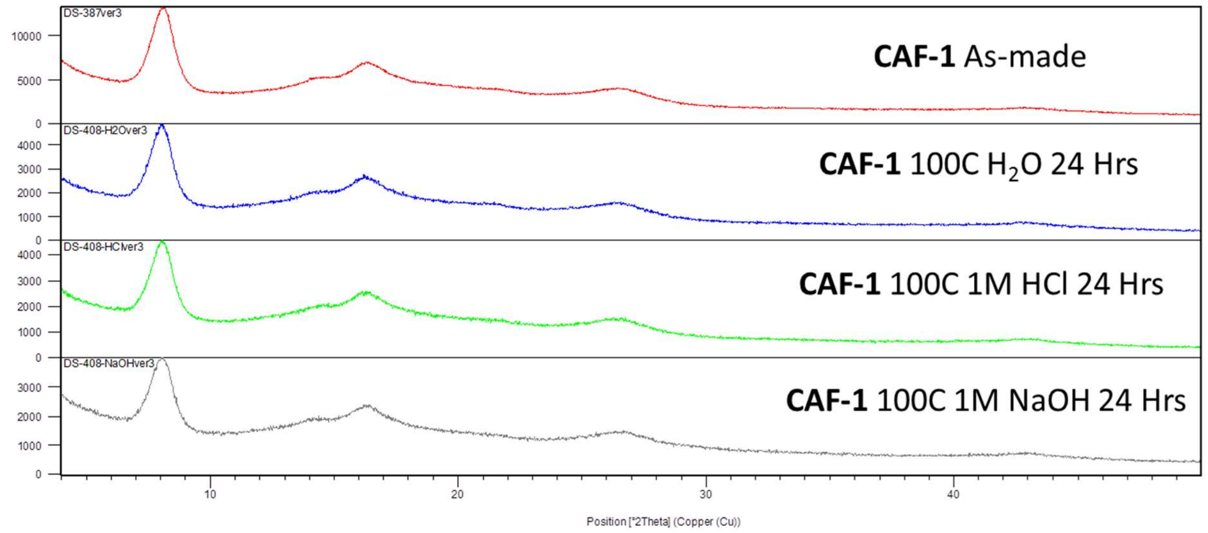

Supplementary Figure 36 – PXRD patterns of CAF-1 after 100 °C stability testing in H_2O (blue), 1M HCl aq. (green), 1M NaOH aq. (black)

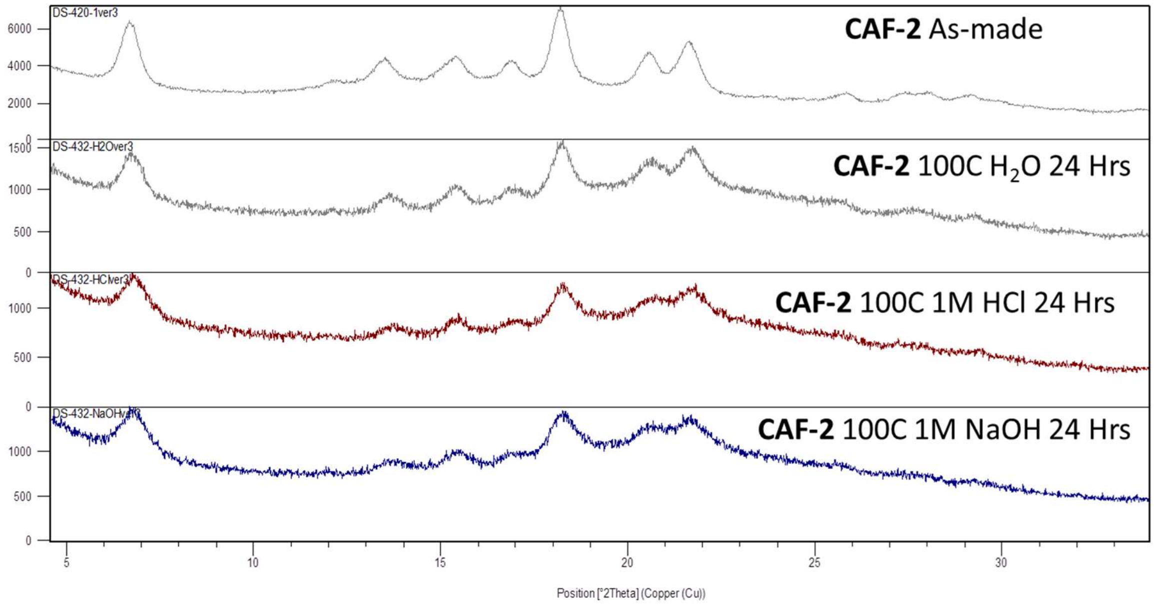

Supplementary Figure 37 - PXRD patterns of CAF-2 after 100 °C stability testing in H_2O (black), 1M HCl aq. (red), 1M NaOH aq. (blue)

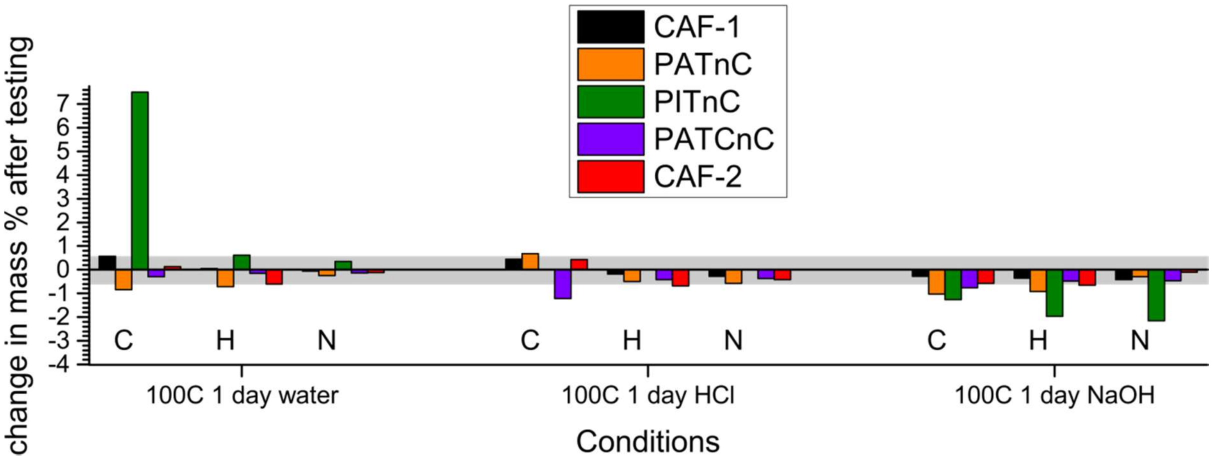

Supplementary Figure 38 Graphical representation of CHN analysis after 100 °C stability testing in H_2O , 1M HCl aq and 1M NaOH aq. Greyed out area corresponds to experimental error (0.56%). In the case of 1M HCl **PITnC** immediately dissolved and thus no data could be plotted for this sample.

Supplementary Note 7 – Room temperature stability testing with concentrated reagents

In order to compare **CAF-1** and **CAF-2** to a different set of state-of-the-art stable imine COFs than those tested at high temperatures (Supplementary Note 6), a further set of stability tests were carried out again literature conditions[27]. Samples of **CAF-1**, **CAF-2**, **PATnC**, **PATCnC** and **PITnC** were suspended in either 4mL of 12M $HCl_{(aq)}$ or 14M $NaOH_{(aq)}$ in 5 mL glass vials with Teflon caps. These were stored in the dark for 7 days followed by the same washing and analysis procedure used for the 100 °C hydrolytic stability testing (Supplementary Note 6). However, unlike for the high temperature stability testing, Soxhlet extraction was not required for the NaOH samples. This is likely because at high temperature the **CAF-1** and **PATnC** 2D networks swell allowing the NaOH to enter the networks but once cooled it becomes trapped, whereas at room temperature the networks do not swell as much thus not trapping excess NaOH in the swollen-then-contracted pores. The same problem of small particle size was also encountered for **CAF-2** in this test and thus the same centrifuge/decanting process was used to work up these samples (Supplementary Note 6). This also resulted in residual suspended material in the supernatant as discussed above, and so the values reported for **CAF-2** are an upper limit for the mass loss. The data from **CAF-2** has been formatted differently in the table below and in Figure 5 to highlight this difference in method.

It is worth noting that due to the highly viscous and dense nature of the 14M NaOH solution it proved very difficult if not almost impossible to suspend the samples. All the samples floated on this solution minimising the contact between the sample and the solution. However for the more hydrophobic imine network, **PITnC**, it proved impossible to even wet the powder with this solution. This observation calls into question the validity of this experiment as it may result in networks which appear highly stable to strong alkali conditions solely because the powders are not in contact with the solution for the duration of the test. In the case of **CAF-1**, **CAF-2**, **PATnC** and **PATCnC** which are less hydrophobic than **PITnC** it was possible to wet the powders with the 14M $NaOH_{(aq)}$ solution however they still floated rather than suspended. We have included this data here for the purposes of a direct comparison to literature materials.

Supplementary Table 13 – Residual masses of **CAF-1**, **PATnC**, **PITnC**, **CAF-2** and **PATCnC** after room temperature stability testing with concentrated reagents

Sample	Conditions	Initial Mass (mg)	Residual Mass (mg)	Residual Mass (%)
**CAF-1**	12M HCl	20.0	18.0	90
**CAF-1**	14M NaOH	20.0	18.3	92
**PATnC**	12M HCl	22.6	22.6	100
**PATnC**	14M NaOH	19.8	18.3	92
**PITnC**	12M HCl	20.0	0	0
**PITnC**	14M NaOH	19.9	18.4	92
**CAF-2**	*12M HCl*	*30.2*	*29.7*	*98*
CAF-2	*14M NaOH*	*29.8*	*23.8*	*79.9*
**PATCnC**	12M HCl	30.3	28.9	95.3
**PATCnC**	14M NaOH	31.4	31.2	94.4

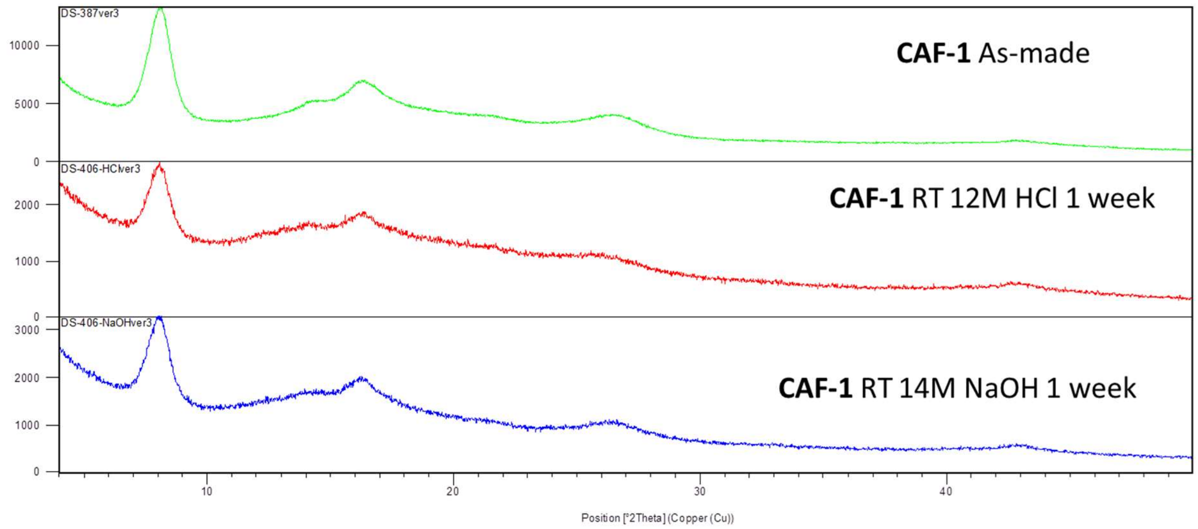

Supplementary Figure 39 PXRD patterns of **CAF-1** after room temperature stability testing with concentrated HCl (red) and concentrated NaOH (blue).

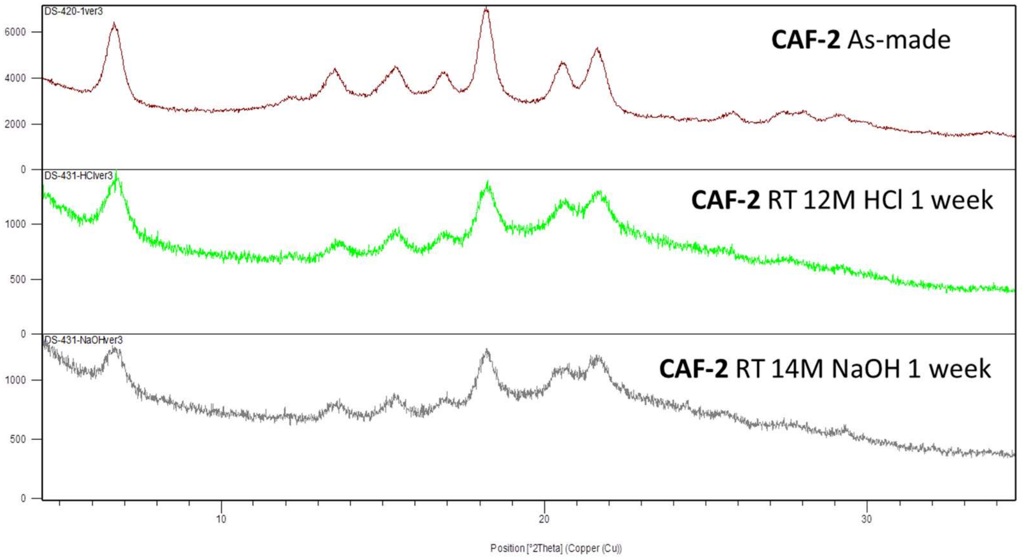

Supplementary Figure 40 PXRD patterns of **CAF-2** after room temperature stability testing with concentrated HCl (green) and concentrated NaOH (gray).

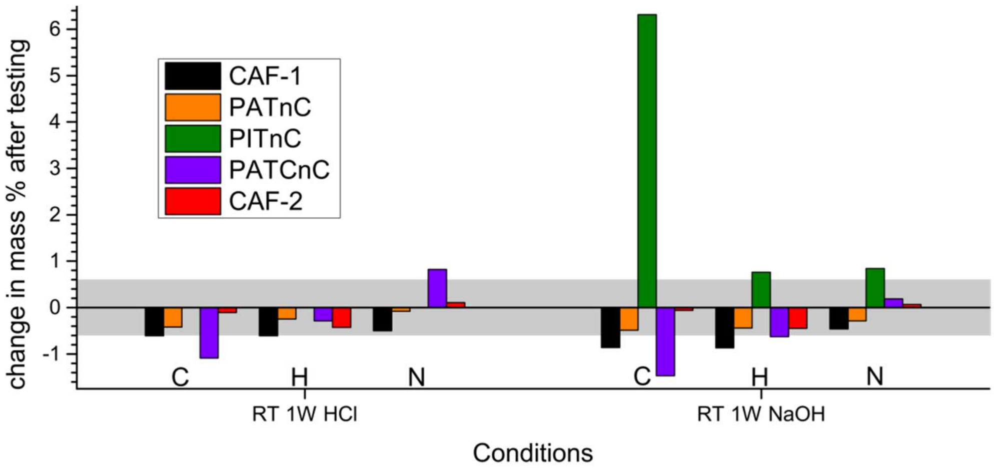

Supplementary Figure 41 Graphical representation of CHN analysis after room temperature stability testing in concentrated HCl and concentrated NaOH. Greyed out area corresponds to experimental error (0.56%). In the case of concentrated HCl **PITnC** immediately dissolved and thus no data could be plotted for this sample.

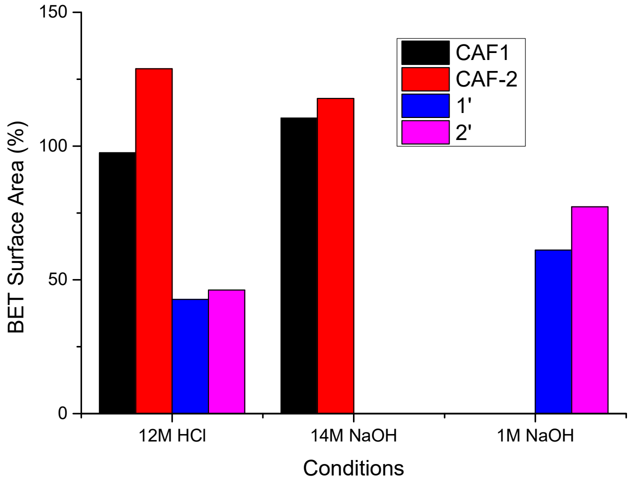

Supplementary Figure 42 Comparison of the residual BET surface area of **CAF-1** and **CAF-2** with those of **1’** and **2’** (See Supplementary Figure 43 for structures) , two-dimensional imine COFs subsequently post-synthetically modified to form amides, as reported in reference [28]. **CAF-1** and **CAF-2** were suspended in highly concentrated 14M NaOH for 1 week whereas the materials in reference 29 were suspended in 1M NaOH for 24 hours.

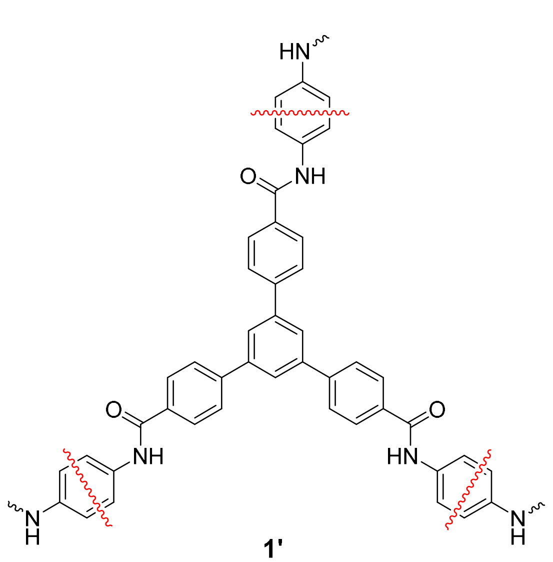

1'

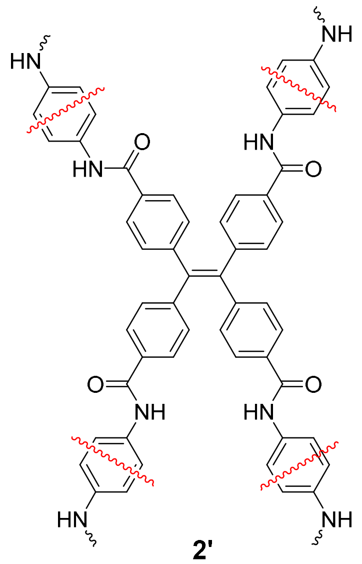

2'

Supplementary Figure 43 Molecular structures of the repeat units of **1’** and **2’** as reported in reference 29

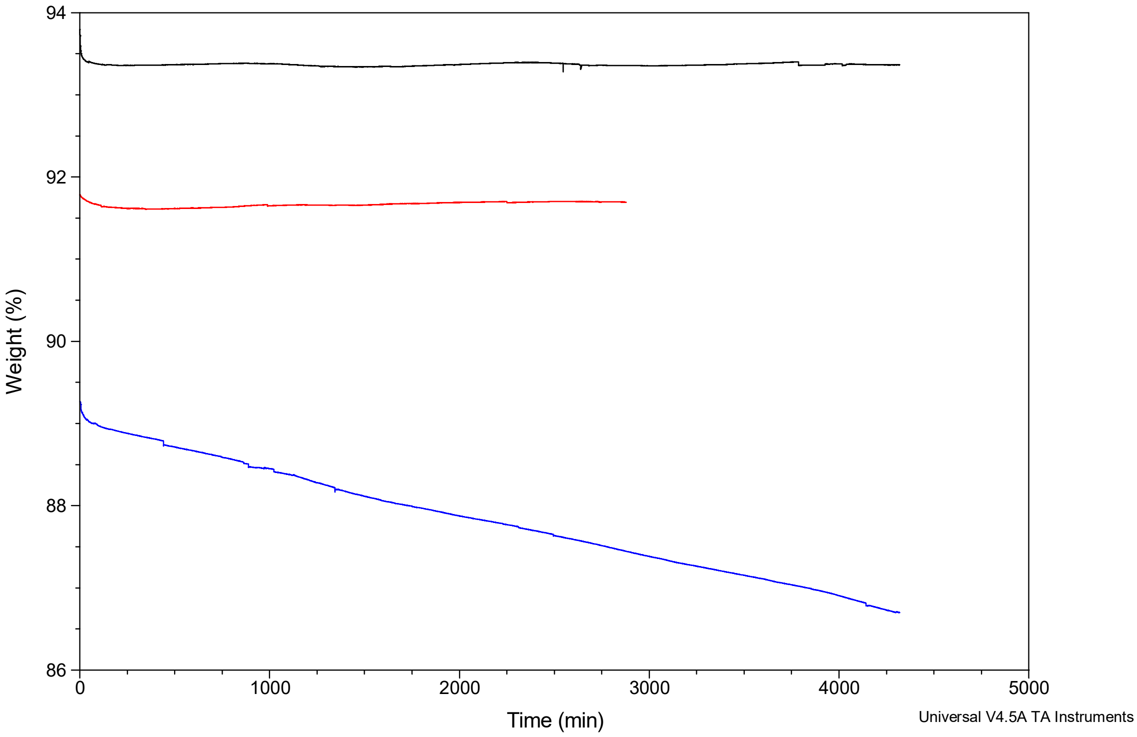

Supplementary Figure 44 Isothermal TGAs of **CAF-1** collected under flowing air (100 mL min^{-1}) at 100 °C (black), 150 °C (red) and 200 °C (blue). PXRD and CHN were then collected for the samples (Supplementary Table 14 and Supplementary Figure 45).

Supplementary Table 14 CHN analysis after thermal stability testing of **CAF-1**

	Difference from calculated								
**Thermal Stability**	**100 °C**			**150 °C**			**200 °C**		
	**C**	**H**	**N**	**C**	**H**	**N**	**C**	**H**	**N**
Initial	0.15	0.18	0.14	0.15	0.18	0.14	0.15	0.18	0.14
2 Days	-	-	-	0.06	-0.46	-0.47	-	-	-
3 Days	0.13	-0.1	-0.29	-	-	-	-1.6	-1.65	-0.59

Based on the empirical formula $C_{18}H_{21}N_3O_3.xH_2O$ where *x* is varied to achieve the best fit to the data. Numbers reported are the percentage difference between the measured and calculated values, experimental error is 0.4 %.

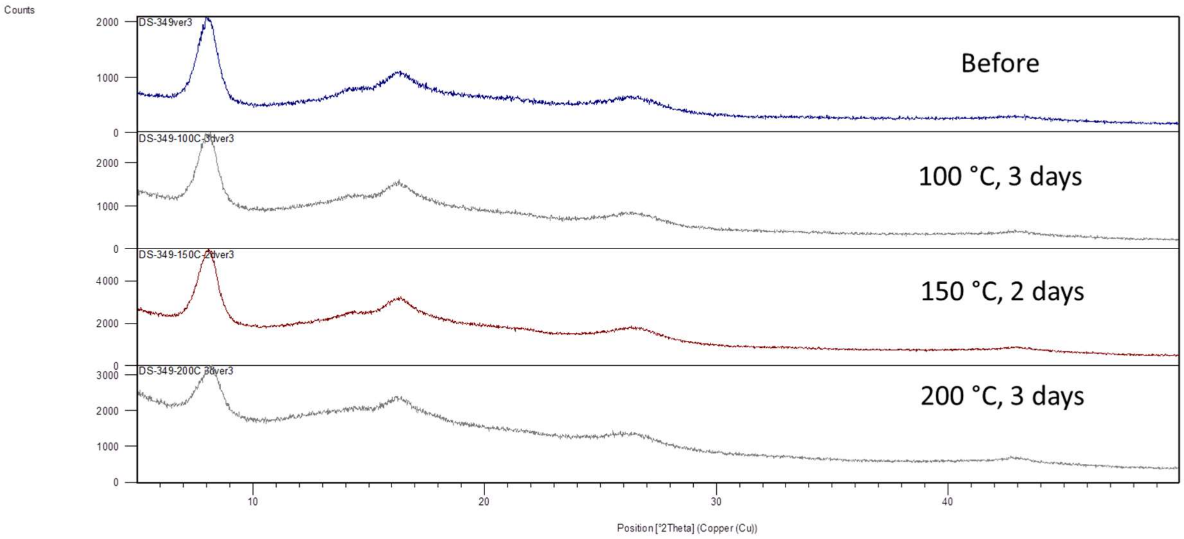

Supplementary Figure 45 PXRD patterns of **CAF-1** after thermal stability testing

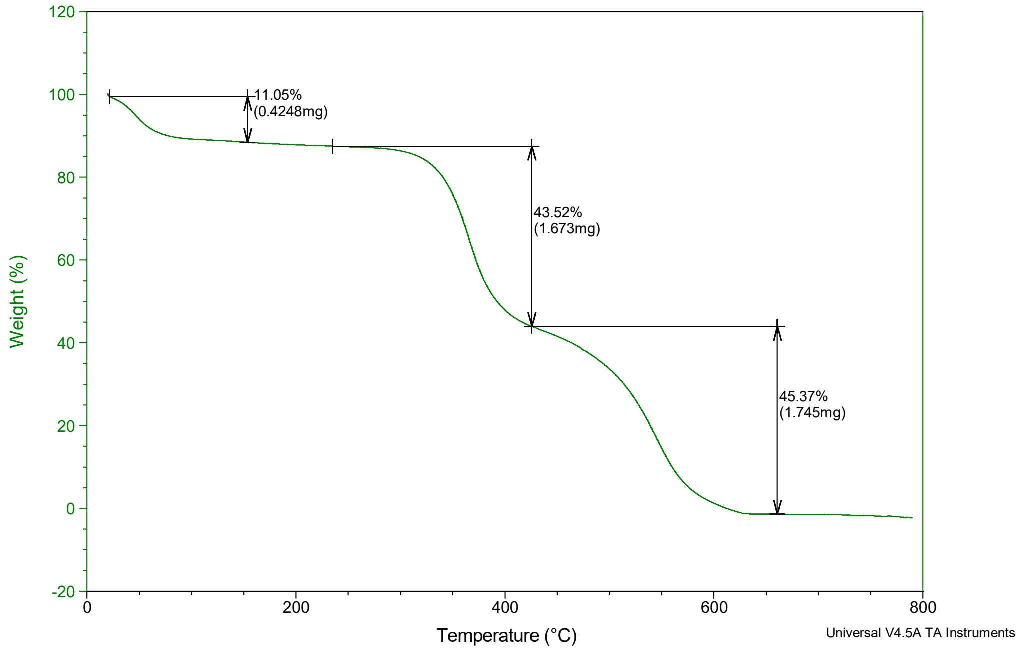

Supplementary Figure 46 TGA of **CAF-1** under flowing air (100 mL min^{-1}) with a ramp rate of 10 °C min^{-1}.

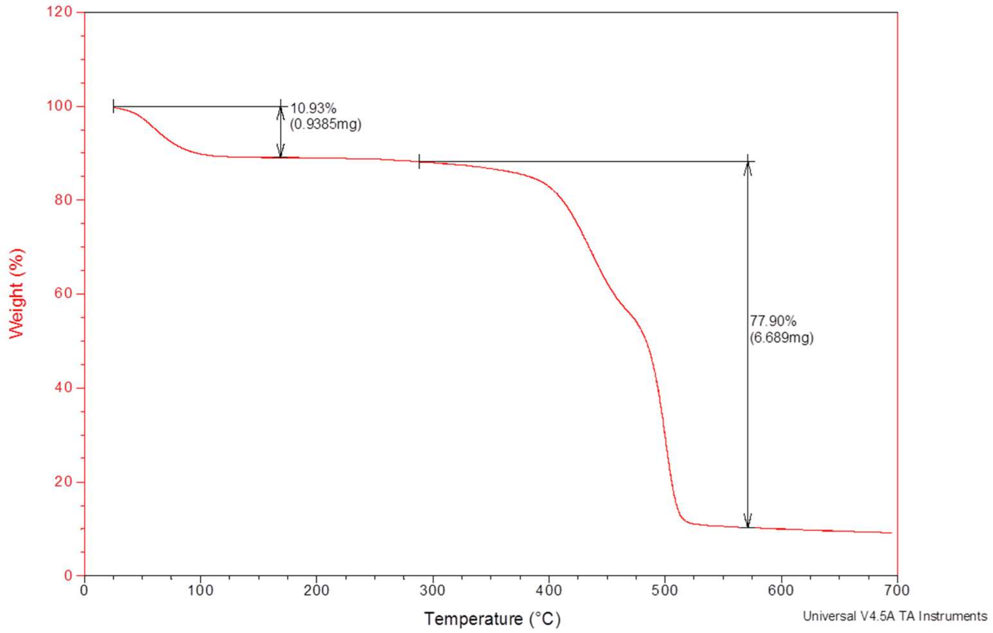

Supplementary Figure 47 TGA of **CAF-1** under flowing N_2 (100 mL min^{-1}) with a ramp rate of 10 °C min^{-1}.

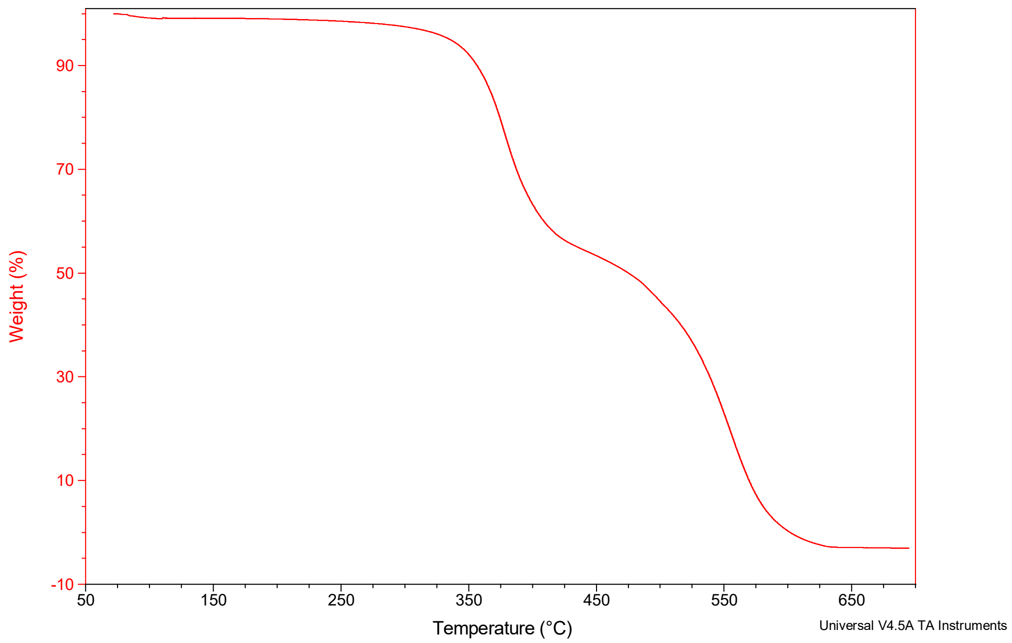

Supplementary Figure 48 TGA of **CAF-1** under flowing air (100 mL min^{-1}) with a ramp rate of 10 °C min^{-1} after activation at 120 °C under 10^{-3} mbar overnight.

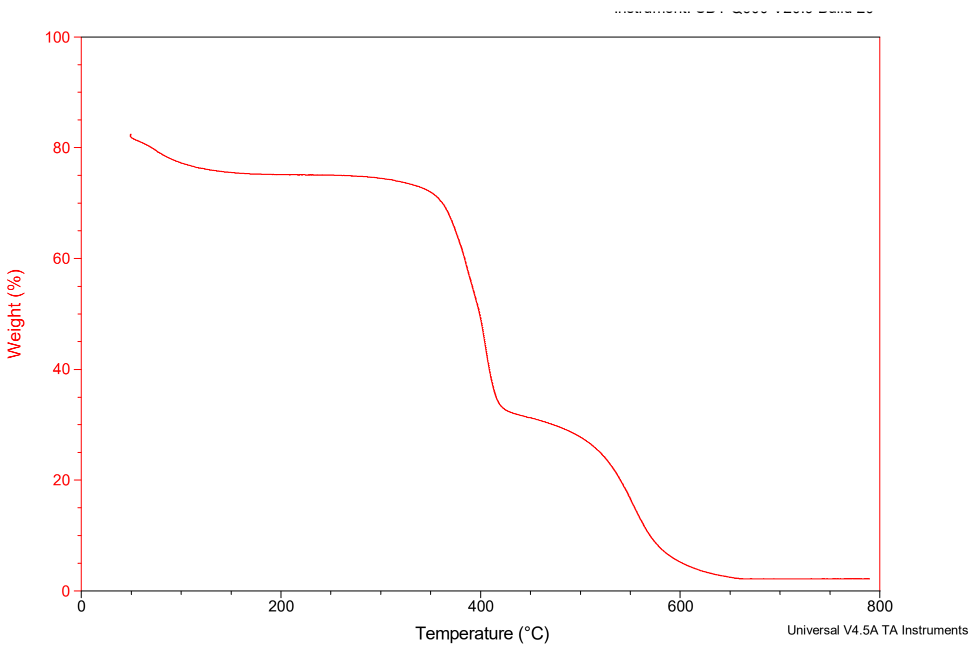

Supplementary Figure 49 TGA of **PATnC** under flowing air (100 mL min^{-1}) with a ramp rate of 10 °C min^{-1}.

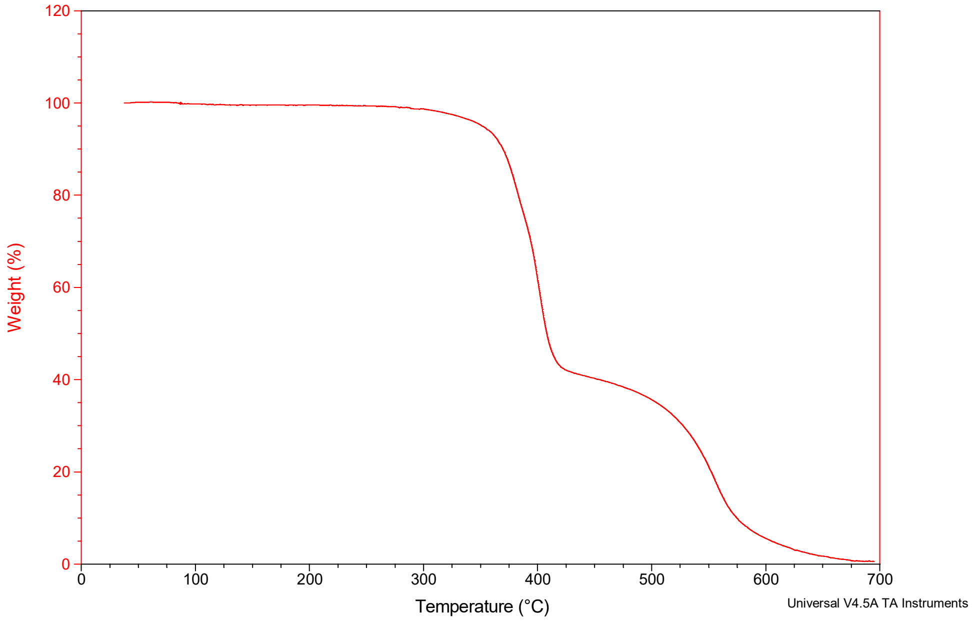

Supplementary Figure 50 TGA of **PATnC** under flowing air (100 mL min^{-1}) with a ramp rate of 10 °C min^{-1} after activation at 120 °C at 10^{-3} mbar overnight.

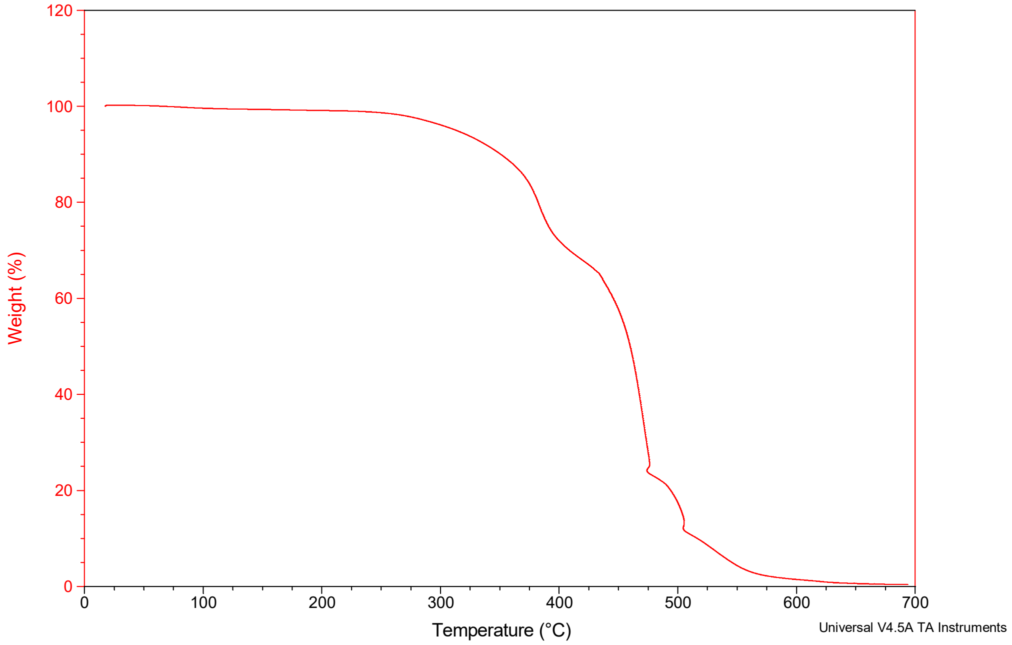

Supplementary Figure 51 TGA of **PATCnC** under flowing air (100 mL min^{-1}) with a ramp rate of 10 °C min^{-1} after activation at 120 °C at 10^{-3} mbar overnight.

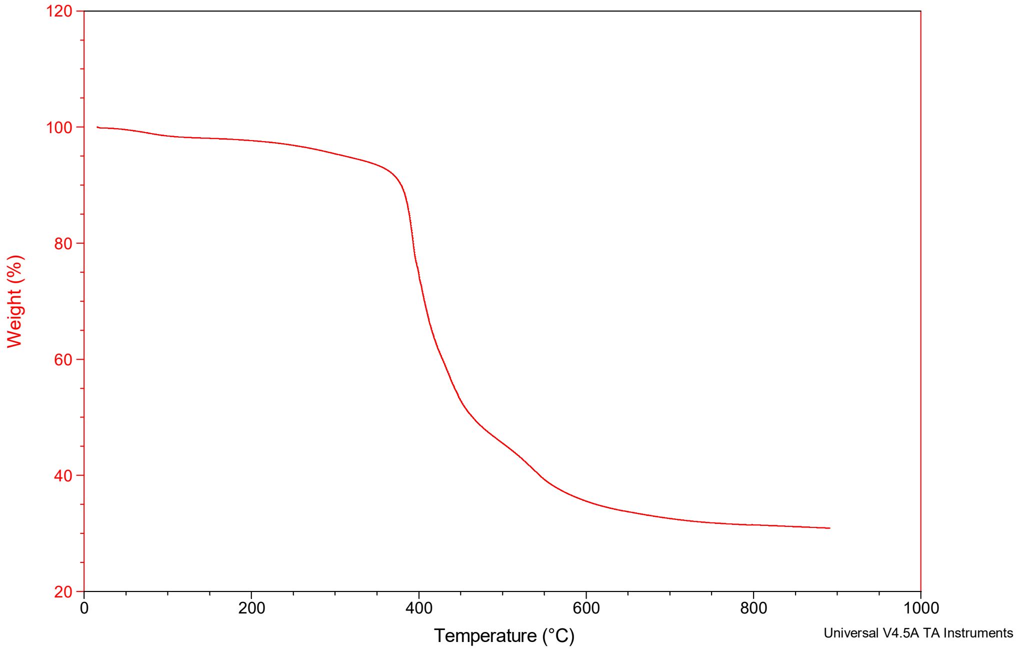

Supplementary Figure 52 TGA of **PATCnC** under flowing N_2 (100 mL min^{-1}) with a ramp rate of 10 °C min^{-1} after activation at 120 °C at 10^{-3} mbar overnight.

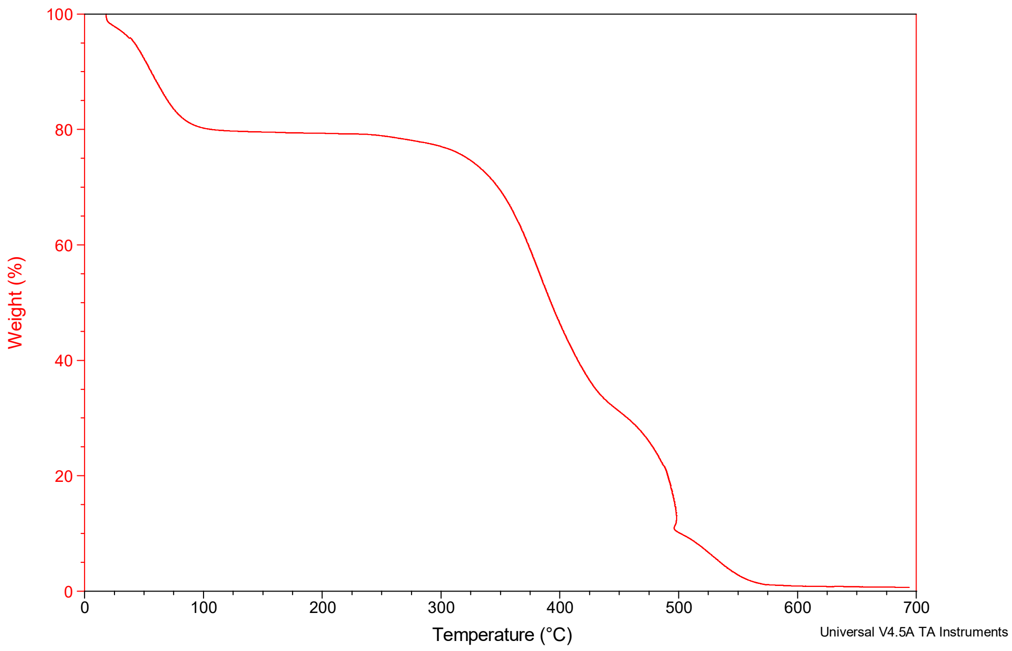

Supplementary Figure 53 TGA of **CAF-2** under flowing air (100 mL min^{-1}) with a ramp rate of 10 °C min^{-1}.

Supplementary Note 8 Imine COF stability literature review

Review based on Web of Science search using the terms:

imine AND

COF OR Covalent Organic Framework

Only COFs synthesised in the bulk were considered; this search was last updated on 14/12/2016.

One of the main methods used to improve the stability of imine COFs to date has been the utilization of an imine/enamine tautomerism[29] (shown in Supplementary Figure 54) which occurs after the network has formed reversibly. This tautomerism effectively eliminates the reversibility of the imine bond after synthesis thus making the networks more stable. An ***X*** in the **enamine** column in Supplementary Data 1 indicates that the material utilises the imine-enamine tautomerism as described above.

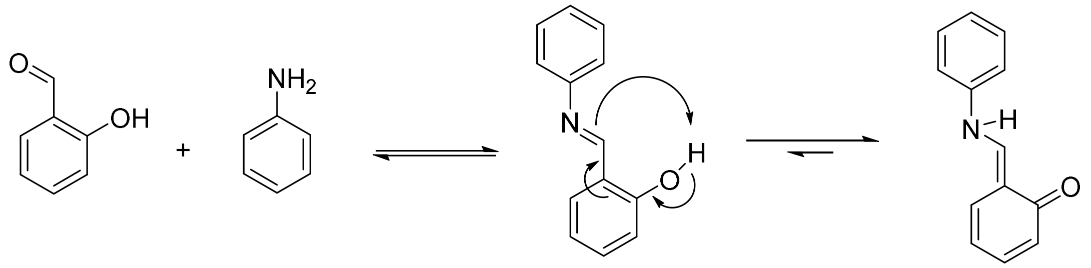

Supplementary Figure 54 Imine – enamine tautomerism

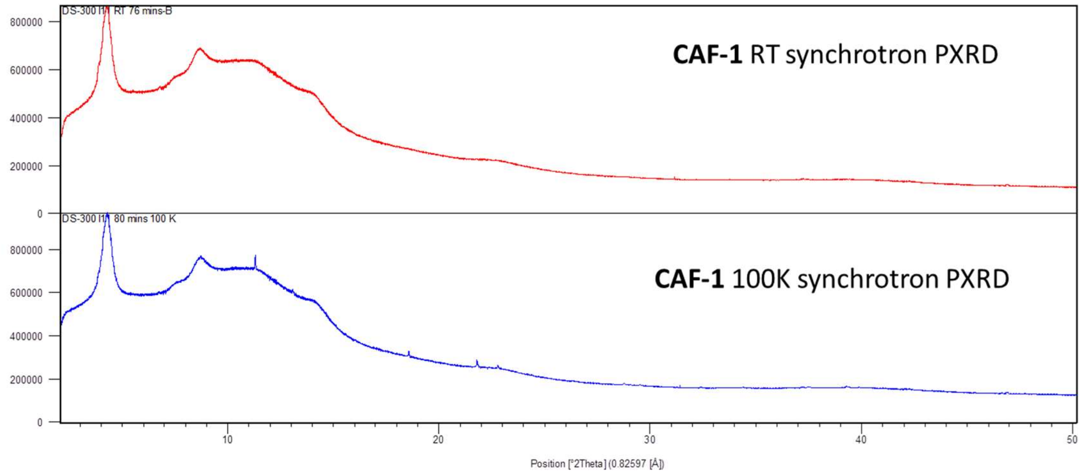

Supplementary Figure 55- There is no observable difference between the PXRD of **CAF-1** measured at 100K (blue) compared to that measured at RT (red). It is therefore unlikely that dynamical disorder within **CAF-1** contributes to the observed room temperature diffraction pattern.

Supplementary References

1. Fung, B.M., Khitrin, A.K. & Ermolaev, K. An improved broadband decoupling sequence for liquid crystals and solids. *J. Magn. Reson.* **142**(1), 97-101 (2000).

2. Morcombe, C.R. & Zilm, K.W. Chemical shift referencing in MAS solid state NMR. *J. Magn. Reson.* **162**(2), 479-486 (2003).

3. Bertani, P., Raya, J. & Bechinger, B. 15N chemical shift referencing in solid state NMR. *Solid State Nucl. Magn. Reson.* **61–62**, 15-18 (2014).

4. Thommes, M. & Cychosz, K.A. Physical adsorption characterization of nanoporous materials: Progress and challenges. *Adsorption* **20**(2-3), 233-250 (2014).

5. Thompson, S.P. *et al.* Beamline I11 at diamond: A new instrument for high resolution powder diffraction. *Rev. Sci. Instrum.* **80**(7), 075107 (2009).

6. Soper, A.K. & Barney, E.R. Extracting the pair distribution function from white-beam X-ray total scattering data. *J. Appl. Cryst.* **44**(4), 714-726 (2011).

7. Coelho, A.A., Chater, P.A. & Kern, A. Fast synthesis and refinement of the atomic pair distribution function. *J. Appl. Cryst.* **48**(3), 869-875 (2015).

8. Qiu, X., Bozin, E.S., Juhas, P., Proffen, T. & Billinge, S.J.L. Reciprocal-space instrumental effects on the real-space neutron atomic pair distribution function. *J. Appl. Cryst.* **37**(1), 110-116 (2004).

9. Kodama, K., Iikubo, S., Taguchi, T. & Shamoto, S.-i. Finite size effects of nanoparticles on the atomic pair distribution functions. *Acta Cryst. A* **62**, 444-453 (2006).

10. Kresse, G. & Furthmüller, J. Efficient iterative schemes for *ab initio* total-energy calculations using a plane-wave basis set. *Phys. Rev. B* **54**(16), 11169-11186 (1996).

11. Clark, S.J. *et al.* First principles methods using castep. *Z. Kristall.* **220**(5-6), 567-570 (2005).

12. Klimeš, J., Bowler, D.R. & Michaelides, A. Van der waals density functionals applied to solids. *Phys. Rev. B* **83**(19), 195131 (2011).

13. Kresse, G. & Joubert, D. From ultrasoft pseudopotentials to the projector augmented-wave method. *Phys. Rev. B* **59**(3), 1758-1775 (1999).

14. Stokes, H.T. & Hatch, D.M. Findsym: Program for identifying the space-group symmetry of a crystal. *J. Appl. Cryst.* **38**(1), 237-238 (2005).

15. Pickard, C.J. & Mauri, F. All-electron magnetic response with pseudopotentials: NMR chemical shifts. *Phys. Rev. B* **63**(24), 245101 (2001).

16. Perdew, J.P., Burke, K. & Ernzerhof, M. Generalized gradient approximation made simple. *Phys. Rev. Letts.* **77**(18), 3865-3868 (1996).

17. Johnston, J.C., Iuliucci, R.J., Facelli, J.C., Fitzgerald, G. & Mueller, K.T. Intermolecular shielding contributions studied by modeling the C13 chemical-shift tensors of organic single crystals with plane waves. *J. Chem. Phys.* **131**(14), 144503 (2009).

18. Aradi, B., Hourahine, B. & Frauenheim, T. DFTb+, a sparse matrix-based implementation of the DFTb method. *J. Phys. Chem. A* **111**(26), 5678-5684 (2007).

19. Elstner, M. *et al.* Self-consistent-charge density-functional tight-binding method for simulations of complex materials properties. *Phys. Rev. B* **58**(11), 7260-7268 (1998).

20. Elstner, M., Hobza, P., Frauenheim, T., Suhai, S. & Kaxiras, E. Hydrogen bonding and stacking interactions of nucleic acid base pairs: A density-functional-theory based treatment. *J. Chem. Phys.* **114**(12), 5149-5155 (2001).

21. Hexem, J.G., Frey, M.H. & Opella, S.J. Influence of N-14 on C-13 NMR-spectra of solids. *J. Am. Chem. Soc.* **103**(1), 224-226 (1981).

22. Biswal, B.P. *et al.* Mechanochemical synthesis of chemically stable isoreticular covalent organic frameworks. *J. Am. Chem. Soc.* **135**(14), 5328-5331 (2013).

23. Dolomanov, O.V., Bourhis, L.J., Gildea, R.J., Howard, J.A.K. & Puschmann, H. Olex2: A complete structure solution, refinement and analysis program. *J. Appl. Cryst.* **42**(2), 339-341 (2009).

24. Uribe-Romo, F.J. *et al.* A crystalline imine-linked 3-D porous covalent organic framework. *J. Am. Chem. Soc.* **131**(13), 4570-4571 (2009).

25. Campbell, B.J., Stokes, H.T., Tanner, D.E. & Hatch, D.M. Isodisplace: A web-based tool for exploring structural distortions. *J. Appl. Cryst.* **39**(4), 607-614 (2006).

26. Dalapati, S. *et al.* Rational design of crystalline supermicroporous covalent organic frameworks with triangular topologies. *Nat Commun.* **6**, (2015).

27. Xu, H., Gao, J. & Jiang, D.L. Stable, crystalline, porous, covalent organic frameworks as a platform for chiral organocatalysts. *Nat. Chem.* **7**(11), 905-912 (2015).

28. Waller, P.J. *et al.* Chemical conversion of linkages in covalent organic frameworks. *J. Am. Chem. Soc.* **138**(48), 15519-15522 (2016).

29. Kandambeth, S. *et al.* Construction of crystalline 2D covalent organic frameworks with remarkable chemical (acid/base) stability via a combined reversible and irreversible route. *J. Am. Chem. Soc.* **134**(48), 19524-19527 (2012).
