# Supplementary material for: Stable and ordered amide frameworks synthesised under reversible conditions which facilitate error checking
Source: Nat Commun. 2017 Oct 24;8:1102. doi: 10.1038/s41467-017-01423-5 (PMC5654755; doi:10.1038/s41467-017-01423-5)
Supplement: Supplementary file 2 — Description of Additional Supplementary Files [file 41467_2017_1423_MOESM2_ESM.pdf]

Description of Additional Supplementary Files

File Name: Supplementary Data 1
Description: Imine COF stability literature review. This data summarizes all the known imine based COF and the stability tests have been performed with them. The search has been done on the Web of Science using the terms imine AND COF OR Covalent Organic Framework. An X in the enamine column indicates that the material utilises the imine-enamine tautomerism as described in Supplementary Fig. 54.
